# A novel CD44-targeting aptamer recognizes chemoresistant mesenchymal stem-like TNBC cells and inhibits tumor growth

**DOI:** 10.1016/j.bioactmat.2025.04.027

**Published:** 2025-04-25

**Authors:** Alessandra Caliendo, Simona Camorani, Luis Exequiel Ibarra, Gabriella Pinto, Lisa Agnello, Sandra Albanese, Antonietta Caianiello, Anna Illiano, Rosaria Festa, Vincenzo Ambrosio, Giosuè Scognamiglio, Monica Cantile, Angela Amoresano, Monica Fedele, Antonella Zannetti, Laura Cerchia

**Affiliations:** a Institute of Endotypes in Oncology, Metabolism and Immunology "Gaetano Salvatore", National Research Council, 80131, Naples, Italy; b Institute of Environmental Biotechnology and Health (INBIAS), National University of Rio Cuarto (UNRC), National Council for Scientific and Technological Research (CONICET), Río Cuarto, X5800BIA, Argentina; c Dipartimento di Scienze Chimiche Università di Napoli Federico II, Consorzio Interuniversitario Istituto Nazionale Biostrutture e Biosistemi, Roma, Italy; d Institute of Biostructures and Bioimaging, National Research Council, 80145, Naples, Italy; e Institutional Biobank-Scientific Directorate, National Cancer Institute INT-IRCCS Fondazione G. Pascale, 80131, Naples, Italy

**Keywords:** Aptamer, CD44, Chemoresistant triple-negative breast cancer, Biomarker identification, Targeted cancer therapy

## Abstract

Triple-negative breast cancer (TNBC) represents a significant therapeutic challenge owing to the scarcity of targeted medicines and elevated recurrence rates. We previously reported the development of the nuclease-resistant RNA sTN58 aptamer, which selectively targets TNBC cells. Here, sTN58 aptamer was employed to capture and purify its binding target from the membrane protein fraction of cisplatin-resistant mesenchymal stem-like TNBC cells. Mass spectrometry in conjunction with aptamer binding assays across various cancer cell lines identified CD44 as the cellular target of sTN58. By binding to CD44, sTN58 inhibits the invasive growth and hyaluronic acid-dependent tube formation in chemoresistant TNBC cells, where CD44 serves as a key driver of tumor cell aggressiveness and stem-like plasticity. Moreover, in vivo studies demonstrated the aptamer's high tumor targeting efficacy and its capacity to significantly inhibit tumor growth and lung metastases following intravenous administration in mice with orthotopic TNBC. Overall, our findings reveal the striking potential of sTN58 as a targeting reagent for the recognition and therapy of cancers overexpressing CD44.

## Highlights

• sTN58 specifically targets CD44 expressed on chemoresistant and mesenchymal stem-like TNBC cells.
• sTN58 inhibits the hyaluronic acid-dependent invasive cell growth and tube formation in 3D cultures.
• sTN58 actively accumulates in CD44-positive orthotopic tumors following intravenous injection in mice.
• sTN58 efficiently hampers tumor growth and lung metastases by blocking CD44-mediated signaling, without inducing toxicity.
• This study introduces sTN58 as a novel tool for targeted diagnosis and therapy of chemoresistant TNBC.

## Graphical abstract

**Figure:**
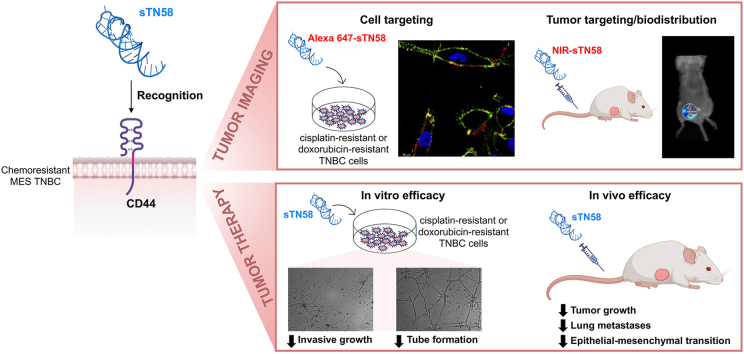

## 1 Introduction

Triple-negative breast cancer (TNBC) represents a distinct subtype of breast cancer (BC) characterized by the lack of expression of estrogen receptor (ER) and progesterone receptor (PR), and the absence of human epidermal growth factor receptor 2 (HER2) amplification [1]. It accounts for approximately 15–20 % of all diagnosed BCs and is associated with high rates of recurrence and poor prognosis [2,3]. The ineligibility for hormone receptor blockers or HER2-targeted agents, coupled with the high tumor heterogeneity and the limited availability of targeted therapies for TNBC patients, poses a significant clinical challenge. Conventional chemotherapy remains the mainstay of treatment for the majority of patients; however, approximately 50 % of tumors develop resistance following an initial response to chemotherapeutics. This resistance is often associated with processes such as epithelial-to-mesenchymal transition (EMT) and the acquisition of stem-like traits, which enhance the tumor's capacity to metastasize and evade therapy. Consequently, fewer than 30 % of patients with metastatic TNBC survive beyond 5 years after diagnosis [2,3]. These challenges underscore the urgent need to identify novel, actionable targets that can overcome drug resistance and to develop more effective targeted strategies with high efficacy against resistant cancer cells and minimal toxicity.

Oligonucleotide aptamers, generated by cell-SELEX (Systematic Evolution of Ligands by Exponential Enrichment) technology, serve as a reliable instrument for the identification of novel cancer biomarkers and their targeting with advanced aptamer-based treatment approaches [4,5]. The primary advantage of cell-SELEX is its ability to identify aptamers targeting a tumor cell type without prior knowledge of the protein targets expressed on the cell surface. By modifying selection procedures for target cells and using subtractive counter-selections for non-target cells, aptamers that precisely bind to the desired cells can be identified [5]. Following SELEX, aptamers may serve as bait in affinity chromatography for the identification of novel biomarkers associated with certain disease conditions [4].

We previously applied a SELEX approach on TNBC cells, resulting in the production of six nuclease-resistant 2′Fluoro-pyrimidines (2′F-Pys) RNA aptamers. These aptamers demonstrated remarkable efficacy in targeting a series of cultured cell lines and clinical patient samples across different TNBC subtypes, distinguishing them from both normal samples and triple-positive breast cancers (TPBC, ER^+^, PR^+^, HER2 over-expression) [6]. The optimization of these aptamers led to the development of variants ranging from 49 to 40 nucleotides (nt) in length, shorter than the original 84 nt. These shorter versions preserve the cellular targeting and uptake capabilities of the full-length aptamers [7], facilitating the delivery of drug-loaded nanoparticles specifically to TNBC cells [8,9]. These aptamers, which bind to cytomembrane proteins on mesenchymal-like (MES) chemoresistant TNBC cells, efficiently inhibit their proliferation and EMT capability [6,7]. This suggests their ability to target critical surface biomarkers specific to TNBC, underscoring their potential therapeutic relevance. One of these aptamers, namely sTN58, has an exquisite selectivity for TNBC cells that have developed resistance to cisplatin (Cis-Pt) and doxorubicin (Dox) due to prolonged exposure to these drugs. These resistant cells exhibit a high abundance of cancer stem cells (CSCs) and enhanced EMT characteristics compared to their parental counterparts [6,7,10].

CD44, the main surface receptor for hyaluronic acid (HA), has a crucial role in the aggressiveness and chemoresistance of TNBC and other human tumors [11,12]. It is recognized as a mesenchymal stem cell marker [13,14] and is abundantly expressed in CSCs, which are particularly enriched in TNBC, as well as in other aggressive tumors, playing a critical role in tumor initiation, progression and metastasis [15]. While chemotherapy efficiently targets most cancer cells, it fails to eradicate CSCs, which are the primary drivers of therapeutic resistance and recurrence. Directly targeting the tumor stem cell population represents a promising and potentially the most efficacious therapeutic approach [16].

To date, not all the aptamers generated by cell-SELEX have already progressed to the target identification, which indeed remains a very challenging task [4,17]. Here, by coupling aptamer-based affinity purification and subsequent mass spectrometry (MS)-based proteomics with a systematic approach that takes advantage of the different binding affinity of sTN58 to different cancer cell lines, we succeeded in identifying CD44 as the target of sTN58. Based on CD44’ roles in modulating stemness and epithelial-mesenchymal plasticity, the aptamer proficiently inhibits invasive growth and HA-induced tube formation in chemoresistant TNBC cells in three-dimensional (3D) culture conditions. Furthermore, in vivo and ex vivo studies demonstrated that sTN58 selectively targets TNBC implanted in mice, strongly interfering with tumor growth and lung metastases. These findings establish a robust foundation for the development of an aptamer-guided methodology for imaging detection and targeted therapy of aggressive and mesenchymal/stem-like TNBC, with potential applicability to other cancer types.

## 2 Materials and methods

### 2.1 Aptamers

2′F-Py-RNA sTN58 [6,7] and a non-related scrambled aptamer (SCR) used as a negative control [7,18,19], either unmodified or conjugated at 5′ extremity with biotin (biotin-sTN58 and biotin-SCR) or Amino-C6 group (5′-(C6-NH2)-sTN58 and 5′-(C6-NH2)-SCR), were synthesized by LGC Biosearch Technologies (Risskov, Denmark). The sequences of sTN58 and SCR are the following:

sTN58: 5′GGACAUAUGAUGCAACGUUGUGGUCCCGUUUGCACUUUGUUUACG3'.

SCR: 5′UUCGUACCGGGUAGGUUGGCUUGCACAUAGAACGUGUCA3'.

For cell imaging and flow cytometry analyses, sTN58 and SCR were internally labeled with Alexa Fluor 647 (Invitrogen, Carlsbad, CA, USA), as previously reported [6,7].

For in vivo imaging, amino-terminated aptamers were labeled with VivoTag-S 750 NIR-dye (PerkinElmer, Waltham, MA) as previously described [19].

### 2.2 Aptamer stability in serum

To assess the stability of sTN58 in human serum, 5 μM sTN58 was incubated in Dulbecco's phosphate-buffered saline solution (DPBS, Sigma-Aldrich, Milan, Italy) containing 80 % human serum (Sigma-Aldrich) at 37 °C up to 96 h and RNA integrity was checked as we previously reported [7].

### 2.3 Cell lines

All cell lines used in this study were purchased from the American Type Culture Collection (ATCC, Manassas, VA, USA). Human MES-TNBC MDA-MB-231 (HTB-26™) and BT-549 (HTB-122™) cells, murine TNBC 4T1 (CRL-2539™) cells, and human THP-1 (TIB-202™) monocytes (grown in suspension) were grown in Roswell Park Memorial Institute-1640 medium (RPMI-1640, Sigma-Aldrich) supplemented with 10 % fetal bovine serum (FBS). Human luminal B/HER2-positive BC BT-474 (HTB-20™) cells were grown in ATCC HybriCare Medium supplemented with 10 % FBS. Luminal A/ER and PR-positive BC MCF-7 (HTB-22™), epidermoid cancer A-431 (CRL-1555™) and fibroblast HS-5 (CRL-3611™) cell lines were grown in Dulbecco's modified Eagle's medium (DMEM, Sigma-Aldrich) supplemented with 10 % FBS. The human mammary epithelial MCF 10A (CRL-10317™) cells were grown in DMEM/Ham's Nutrient Mixture F-12 (DMEM/F-12, Sigma-Aldrich) supplemented with 5 % horse serum (ThermoFisher Scientific, Waltham, MA, USA), 0.5 μg/mL hydrocortisone, 20 ng/mL epidermal growth factor, 10 ng/mL cholera toxin and 10 μg/mL insulin (Sigma-Aldrich).

Cisplatin-resistant (Cis-Pt-R) and doxorubicin-resistant (Dox-R) cell lines were generated by a prolonged treatment of MDA-MB-231 with drugs and cultured as previously described [6,10]. Cells were treated with increasing doses of Cis-Pt (ranging from 100 nM to 200 μM) or Dox (ranging from 100 nM to 100 μM) for 48 h at 37 °C and verified for increased resistance to the chemotherapeutics respect to parental cells, by cell viability assays and EC50 value calculation, as previously reported [6,10]. All cells were maintained in 95 % air/5 % CO_2_ atmosphere at 37 °C.

### 2.4 Aptamer-mediated pull-down assay

Protein identification was performed with an adapted protocol as previously described [20]. Membrane proteins (500 μg) were isolated from 12 × 10^6^ Cis-Pt-R cells as described [10] and incubated with 400 nM non-targeting biotin-SCR in DPBS supplemented with proteases inhibitors (Roche Diagnostics, Indianapolis, USA) for 30 min at room temperature (RT) with rotation. The protein-RNA complex was captured by incubation with 150 μg Pierce™ Streptavidin Magnetic Beads (Thermo Fisher Scientific, Waltham, MA, USA) for 1 h at RT. Afterward, the unbound proteins were incubated with 400 nM biotin-sTN58 for 30 min at RT with rotation. Then, protein-sTN58 complex was captured by incubation with Streptavidin Magnetic Beads for 1 h at RT. The beads were washed five times with DPBS and the proteins were eluted by heating in 40 μL of loading buffer and analyzed by 10 % Sodium Dodecyl Sulphate – Polyacrylamide Gel Electrophoresis (SDS-PAGE) stained with Colloidal Blue.

### 2.5 Mass spectrometry

Horizontal slices of SDS-PAGE bands for proteins captured by sTN58 were excised from the gel lane. Gel destaining consisted of the three consecutive cycles of 0.1 M ammonium bicarbonate at pH 8.0 and acetonitrile, followed by reduction (10 mM dithiothreitol in 100 mM ammonium bicarbonate, 45 min at 56 °C) and alkylation (55 mM iodoacetamide in 100 mM ammonium bicarbonate, 30 min at RT). The gel pieces were washed with three further cycles of 100 mM ammonium bicarbonate of pH 8.0 and acetonitrile. Finally, the gel pieces were subjected to enzymatic hydrolysis by covering them with 40 μL sequencing grade modified trypsin (10 ng/μL trypsin; 10 mM ammonium bicarbonate) and incubated overnight at 37 °C. Peptide mixtures were eluted, vacuum-dried, and resuspended in 2 % acetonitrile acidified with 0.1 % formic acid before liquid chromatography tandem mass spectrometry (LC-MS/MS). The peptide mixtures were injected into a Linear Trap Quadrupole Orbitrap XL (Thermo Fisher Scientific) coupled to a nano-LC system (nanoEasy II). A volume of 3 μL of each sample was loaded onto a C18 capillary reverse-phase column (150 mm, 75 μm, 1.8 μm) and flow was set at a 0.3 μL/min flow rate. The linear gradient of eluent B (0.2 % formic acid in 95 % acetonitrile) in A (0.2 % formic acid and 2 % acetonitrile in MilliQ water) was run from 5 % to 80 % in 80 min. MS/MS analyses were performed by using Data-Dependent Acquisition mode: one MS scan (mass range from 300 to 1800 *m*/*z*) was followed by MS/MS scans of the five most abundant ions in each MS scan, applying a dynamic exclusion window of 45 s. Charge state properties included charges from 2 to 6. MS/MS spectra were acquired with Higher-energy Collisional Dissociation at 30 %. Mass tolerance was set at 10 ppm and intensity threshold at 1exp^4^. Raw data files were processed by using MaxQuant software (1.6.8.0 version) [21]. An experimental design template was used to specify individual or merged replicate experiments (each data set contained two technical replicates) and to combine all raw data from each lane into a single column containing all the proteins in every sample. The following parameters were used for raw data processing: trypsin enzyme specificity, 3 missed tryptic cleavages, oxidation of methionine, and formation of pyroGlu from N-terminal glutamine (Q) or glutamic acid (E), as variable modifications, and cysteine (C) carbamidomethylation as a fixed modification. Identification parameters included a minimum peptide length of 6 amino acids, minimum of 1 peptide (both razor and unique peptide). Peptide tolerance of 10 ppm, fragment mass tolerance of ±0.2 Da. All proteins were filtered according to a false discovery rate of 0.01 % applied both at peptide and protein levels and a maximum peptide posterior error probability of 1. The derived peak list generated by Quant.exe (the first part of MaxQuant) was searched using the Andromeda search engine integrated into the MaxQuant against the Fasta file of Homo sapiens downloaded from the UniProt web site. The MaxQuant file (protein.txt) was further uploaded on Perseus software [22] to perform the statistical analysis. Contaminants, reverse, and only identified by site hits were filtered out. Further filtering criteria consisted of extracting proteins identified with at least 2 peptides, score higher than 40 and sequence coverage higher than 10 %.

### 2.6 Immunoblot

Cell and tumor lysates preparation and immunoblot analyses were performed as previously reported [23,24]. Filters were incubated overnight at 4 °C with the following primary antibodies: CD44 (lysates from human cell lines), Zonula occludens-1 (ZO-1, D7D12), platelet-derived growth factor receptor β (PDGFRβ, 28E1), phospho-44/42 MAPK (extracellular signal-regulated kinase 1/2, ERK1/2, D13.14.4E, indicated as p-ERK1/2), phospho-Akt (Ser473, indicated as p-Akt), Akt, Met (25H2), vimentin (D21H3), E-Cadherin (24E10), vinculin (E1E9V), α-tubulin (DM1A) (Cell Signaling Technology Inc., Danvers, MA, USA); CD44 (lysates from murine 4T1 cells, ab157107), integrin β1 (ITGB1, ab179471) (Abcam, Cambridge, UK); liprin β1, Ephrin Type-A Receptor 2 (EphA2), ERK1 (C-16) (Santa Cruz Biotechnology, Santa Cruz, CA); myoferlin (MYOF, HPA014245, Sigma-Aldrich) and programmed cell death-ligand 1 (PD-L1)/CD274 (Proteintech Group, Inc.). Densitometric analysis was performed on at least two different exposures to assure the linearity of each acquisition using ImageJ software (v1.46r).

### 2.7 Cell transfection

Cis-Pt-R, BT-549 and MDA-MB-231 cells (1.8 × 10^5^) were seeded in 6-well plates and after 24 h were overlaid with the transfection mixtures containing 100 nM small interfering RNAs (siRNAs) targeting CD44 or integrin β1, and Lipofectamine RNAiMAX Reagent (Thermo Fisher Scientific) in Opti-MEM I reduced serum medium (Gibco), according to the manufacturer's instructions of transfection reagent. Human CD44-targeting siRNA (Hs_CD44_10, referred to as si-CD44) was purchased from Qiagen (Hilden, Germany). Human integrin β1 siRNA (si-ITGB1) [25] was purchased from IDT (Coralville, IA, USA). Scrambled non-targeting siRNA (siRNA ctrl, Qiagen) was used as a negative control. After 5 h incubation, complete culture medium was added to the cells and incubation was prolonged up to 48 h. Silencing efficiency was assessed by immunoblotting and flow cytometry.

### 2.8 Cell binding assay by flow cytometry

For cell binding of sTN58, Cis-Pt-R, MDA-MB-231, BT-549, BT-474, A-431, 4T1, MCF 10A, HS-5 and CD44- or integrin β1-interfered cells, harvested using non-enzymatic cell dissociation solution with 0.02 % EDTA (Invitrogen), and suspended THP-1 cells were washed once with DPBS. Then, 2.0 × 10^5^ cells were incubated for 10 min at RT with 150 nM Alexa 647-sTN58 in serum-free medium supplemented with 0.1 mg/mL yeast tRNA and 0.1 mg/mL ultrapure salmon sperm DNA (Invitrogen), as non-specific competitors. Prior incubation with Alexa-647 labeled sTN58, cells were pre-treated for 10 min at RT with a 30-fold excess of unlabeled SCR negative control aptamer. Cells were washed three times with 500 μL DPBS, suspended in 500 μL DPBS and analyzed by flow cytometry (BD Accuri™ C6 or Attune™ NxT Flow Cytometer, Invitrogen). Data analysis was performed using FlowJo software (version 10.0.7).

Binding of 150 nM Alexa 647-sTN58 to Cis-Pt-R, BT-549 and MDA-MB-231 cell lines transfected with si-CD44 or si-ITGB1 was performed after 48 h from transfection using flow cytometry, as described above.

For cell binding of anti-CD44 antibody, 2.0 × 10^5^ cells were left untreated or incubated for 20 min at RT with CD44 phycoerythrin (PE)-conjugated antibody (CD44-PE Ab, IM7, 0.25 μg/test, Santa Cruz Biotechnology) in DPBS supplemented with 0.1 % bovine serum albumin. For cell binding of anti-integrin β1 antibody, 2.0 × 10^5^ cells were left untreated or blocked with 10 % fetal bovine serum (FBS, Sigma-Aldrich) in DPBS for 20 min at RT to minimize nonspecific binding. Then, cells were incubated for 20 min at RT with APC-Cy7 Mouse Anti-Human CD29 antibody (Integrin β1 APC-Cy7 Ab, dilution 1:16000, BD Biosciences, Allschwil, Switzerland) diluted in DPBS, 10 % FBS. Following antibodies incubation, cells were washed three times in DPBS, suspended in 500 μL DPBS and analyzed by flow cytometry, as described above.

### 2.9 Confocal microscopy analyses

For colocalization experiments of sTN58 aptamer and antibodies against cell surface proteins on Cis-Pt-R cells, 8.0 × 10^4^ cells were seeded onto glass coverslips and, following cell adhesion, were incubated with 2 μM Alexa Fluor 647-labeled sTN58 or SCR, used as negative control, in serum-free RPMI-1640 medium for 5 min at RT. After three washes in DPBS, cells were fixed with 4 % paraformaldehyde in DPBS for 20 min and blocked with 5 % FBS in DPBS for 20 min at RT to minimize nonspecific binding. Then, cells were incubated for 1 h at 37 °C with primary antibodies diluted in DPBS, 2 % FBS. Antibodies used were: CD44 (DF1485, Santa Cruz Biotechnology), integrin β1 (R&D system), kinectin 1 (KTN1, A-12, Santa Cruz Biotechnology) and myoferlin (Sigma-Aldrich). For kinectin 1 and myoferlin antibody staining, cells were permeabilized with 0.1 % Triton X-100/DPBS for 5 min prior antibody incubation. Following primary antibody incubation, cells were washed three times with DPBS and incubated with Alexa Fluor 488-labeled secondary antibodies (Invitrogen, dilution 1:200 in DPBS, 2 % FBS) for 30 min at 37 °C. Finally, after three washes with DPBS, cells were subjected to nuclear staining with 1.5 μM of 4′,6-Diamidino-2-phenylindole (DAPI, D9542, Sigma-Aldrich) and mounted with glycerol/DPBS. 8.0 × 10^4^ BT-474 and A431 cells, incubated with sTN58 and CD44 or integrin β1 antibodies as above, were used as negative control cell lines.

For dual immunofluorescence staining of CD44 and integrin β1, 8.0 × 10^4^ Cis-Pt-R or A431 cells, were fixed and blocked as above, and incubated with CD44-PE (IM7, Santa Cruz Biotechnology) and integrin β1 (R&D system) antibodies, as above. After three washes in DPBS, cells were incubated with Alexa Fluor 488 Anti-Mouse for integrin β1 staining, washed three times with DPBS, stained with DAPI and mounted. Samples were visualized by Zeiss LSM 700 META confocal microscopy equipped with a Plan-Apochromat 63x/1.4 Oil DIC objective. Mander's Overlap Coefficients were calculated by using Zeiss LSM Image Browser Software.

### 2.10 3D cell cultures

Cell growth in 3D culture was conducted as described [19]. Briefly, Cis-Pt-R cells (7 × 10^4^ cells/well), in RPMI-1640 containing 2 % FBS and 2 % Matrigel Basement Membrane Matrix Growth Factor Reduced (Corning Incorporate, Corning, NY), were seeded on a 6-well plate pre-coated with a thin layer of Matrigel, in the presence of 500 nM sTN58 or SCR. The aptamers treatment was renewed every 48 h. The culture was maintained for 5 days and cells were analyzed under a phase-contrast microscope. Colonies having two or more invasive protrusions and number of protrusions for each colony were counted as a measure of invasive behavior in at least 10 fields per condition. For tube formation assays, Cis-Pt-R and Dox-R cell lines (7 × 10^4^ cells/well) were treated as above but in the presence of 50 μg/mL low molecular weight HA (180 kDa). At 24 h photographs were taken using a phase-contrast microscope. Complete loops and junctions were quantified by a macro made with the ImageJ software, as previously reported [26].

### 2.11 Cell invasion assay

The cell invasion assay was performed using 24-well Boyden chambers (Corning Incorporate, Corning, NY) with microporous-8 μm membrane coated with 50 μL of diluted Matrigel (1:5 in serum-free medium), as we previously reported [19]. Briefly, after one-night serum starvation, Cis-Pt-R and Dox-R cells (1 × 10^5^ in 100 μL serum-free medium per well) were placed in the top chamber with or without 500 nM sTN58 or SCR, and exposed to medium containing 10 % FBS (lower chamber). The aptamers treatment was renewed at 48 h. After 72 h of incubation at 37 °C in humidified 5 % CO_2_, cells were visualized by staining with 0.1 % crystal violet in 25 % methanol and photographed. Eight randomly selected fields were imaged using a phase contrast microscope, and the cells were counted.

### 2.12 Cell viability assay

Viability of Cis-Pt-R and Dox-R cells (4.0 × 10^3^ cells/well, 96-well plates) was assessed with Thiazolyl Blue Tetrazolium Bromide (MTT, AppliChem GmbH, Darmstadt, Germany), according to the manufacturer's protocol, and expressed as percent of viable treated cells with respect to untreated cells. Data about cell viability were plotted in GraphPad Prism v.8.4.3 to draw a dose–response curve and to determine the EC50, as we previously reported [6,10].

### 2.13 Animal experiments and fluorescent imaging

All experimental procedures complied with the European Communities Council directives (2010/63/EU) and the present study was approved by the Italian Ministry of Health (authorization number 932/2018-PR). To minimize animal suffering all the experimental procedures described were performed under general anesthesia with 2 % isoflurane in 100 % oxygen at 0.8 L/min. Mice were maintained on a diet with a purified, alfalfa-free rodent chow for 15 days before fluorescence imaging to minimize fluorescence in the gut. For tumor targeting with NIR-aptamers, orthotopic 4T1 xenografts in syngeneic mice were realized as previously described [23]. Briefly, 2 × 10^5^ 4T1 cells were re-suspended in 0.1 mL of 1:1 mix of physiological saline and Matrigel and orthotopically injected into the mammary fat pads of five-week-old Female Balb/c mice, which weighed about 20–22 g (Charles River, Milan, Italy). Once tumors became approximately 150 mm^3^ [volume = 0.5 × long diameter × (short diameter)^2^], mice were randomized into two groups (three animals per group). NIR-sTN58 or NIR-SCR, used as a negative control, were injected (0.75 nmol/100 μL) into the lateral tail vein of mice maintained under isoflurane anesthesia. Before and at specified time points, i.e., 15 min, 1 h, 3 h and 24 h after injection of NIR-sTN58 or NIR-SCR, isoflurane anesthetized mice were subjected to fluorescence molecular tomography (FMT) scanning using an FMT 4000 imaging system on the 750 nm channel (PerkinElmer, Waltham, MA) as previously reported [19]. The position and intensity of fluorescence sources were reconstructed in 3D (tomography) using the TrueQuant software package (PerkinElmer), supplied with the FMT4000. Three-dimensional volumes-of interest (VOIs) were drawn around tumor and adjoining background non-tumor area, and the total amount of fluorescence (pmol) was automatically calculated relative to internal standards generated with known concentrations of the appropriate probe (NIR-sTN58 or NIR-SCR). At 24 h, animals were euthanized and the following organs were harvested and acquired in fluorescence reflectance imaging (FRI) on the 750 nm channel: tumor, liver, spleen, kidneys, lungs, heart and the gastrocnemius muscle.

### 2.14 In vivo cancer therapy and immunohistochemistry

To assess the therapeutic effect of sTN58 in vivo, orthotopic 4T1 xenografts in mice were obtained as described above and they were randomized into two groups (five animals per group) that received intravenous injection of 1 nmol negative control SCR or sTN58 at day 0, 3, 5, 7 and 10, based on reference protocols [23,27]. The long and short diameters of the tumors were measured using slide calipers up to day 13 and the body weight was also measured. At day 13, mice were euthanized and tumors from each animal were excised and in part stored in 10 % neutral buffered formalin for immunohistochemistry analyses and in part frozen, using liquid nitrogen, for protein lysis and immunoblot analyses. Formalin-fixed tumors were paraffin embedded and sectioned (4 μm) and two samples per group were stained with hematoxylin and eosin (H&E) or immunostained with anti-Ki-67 antibody (D3B5, Cell Signaling; dilution 1:400; incubated overnight at 4 °C) as reported [23]. Results were interpreted using Nikon Eclipse Ni light microscope (Nikon, Tokyo, Japan). Each slide was reviewed blinded and, to ensure accuracy, the number of Ki-67 positive cells was determined by two independent counts. Lungs from each animal were harvested and stored in 10 % neutral buffered formalin for immunohistochemistry analyses. The evaluation of metastatic involvement in the five lung samples per group was assessed in percentage terms considering the totality of the sampled fragments and evaluating the ratio between the tumor area and the entire lung tissue section.

### 2.15 In vivo pharmacokinetic

sTN58 (1 nmol/100 μL) was administered intravenously through the tail vein of three healthy Balb/c nude mice. Blood was harvested from the orbital sinus before administration, and at 5, 15, 30, 60, 180, 300 min and 24 h post-administration. Samples were centrifuged for 10 min at 1000×*g*, and plasma was stored at −80 °C until the assay. sTN58 concentration was then determined by quantitative RT-PCR, as previously described [19].

### 2.16 Statistical analysis

Statistics were analyzed using GraphPad Prism version 6.00 by student's t-test (two variables) or one-way analysis of variance (ANOVA) followed by Tukey's multiple comparison test (more than two variables). A p-value <0.05 was considered significant for all analyses.

## 3 Results

### 3.1 sTN58 aptamer-mediated affinity purification for biomarker discovery

To identify TNBC-specific cell membrane markers, we screened a 2′F-Py-containing RNA library on cultured MDA-MB-231 cells, which exemplify the highly malignant and invasive MES subtype. This process yielded a panel of aptamers that specifically bind to the target cells while distinguishing them from BT-474 epithelial TPBC and A431 epidermoid carcinoma cell lines, used in the counterselection steps [6]. Among these, the TN58 aptamer, displayed the highest affinity and specificity for doxorubicin- and cisplatin-resistant MDA-MB-231 derivative cell lines, Dox-R and Cis-Pt-R, respectively [6,7,9], indicating its binding to a protein enriched in chemoresistant cells. By harnessing different binding assays, we demonstrated that the target of the aptamer is a cell membrane protein [6,7,9] and efforts were carried out to identify this target.

**Fig. 1:**
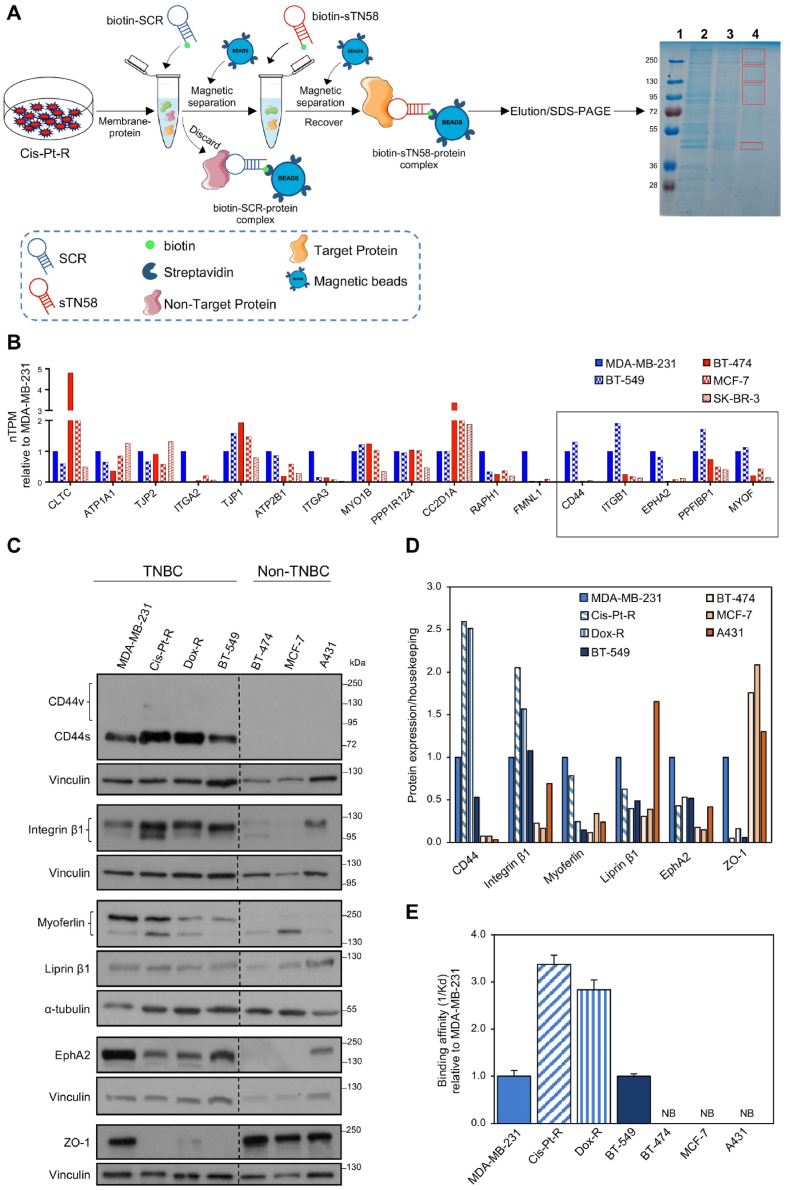
Identification of the target of sTN58 aptamer on TNBC cell surface. (A) Schematic representation of biotin-sTN58-mediated affinity purification. Membrane-protein fraction from Cis-Pt-R cells were subjected to a preclearing step to remove non-specific components prior to the sTN58-mediated precipitation. The colloidal Blue-stained SDS-PAGE (10 %) displayed is utilized for the analysis of target purification mediated by the sTN58 aptamer. The molecular weights of protein markers are reported. Lane 1, molecular markers; lane 2, membrane extracts; lane 3, 15 μg aliquot of unbound proteins from SCR-mediated purification; lane 4, proteins captured with sTN58. Red boxes indicate the regions excised for MS analyses. (B) Comparison of transcript expression values of best candidates in different BC cell lines. The normalized transcript expression values (nTPM), according to HPA, are reported relative to MDA-MB-231 target cells, whose expression level is arbitrarily set to 1. Box indicates the 5 candidates chosen for experimental validation. (C) Immunoblot analysis of EphA2, CD44, integrin β1, myoferlin, liprin β1 and ZO-1, and of the housekeeping proteins α-tubulin and vinculin. The molecular weights of protein markers are reported. Black dashed lines delineate the boundary between non-contiguous lanes of the same gel. (D) The histogram shows the relative fold-change in expression levels of the indicated proteins compared to the housekeeping protein α-tubulin or vinculin, normalized to MDA-MB-231 target cells, whose expression level is arbitrarily set to 1. (E) Binding affinity (1/Kd) of TN58 aptamer to the indicated cell lines expressed relative to MDA-MB-231 target cells. Dose response curves and binding affinity calculations for MDA-MB-231 and Cis-Pt-R and Dox-R chemoresistant cells, as well as non-TNBC BT-474, MCF-7 and A431 cells were previously reported [6,7]. The dose response curve used for Kd calculation in relation to BT-549 is shown in Fig. S2. "NB", no binding.

To this aim, Cis-Pt-R cells were lysed, and the membrane content was subjected to aptamer-mediated affinity purification by adapting a previously described protocol [20]. Initially, the membrane fraction was incubated with a biotinylated 2′F-Py-RNA scrambled aptamer (biotin-SCR), which lacks affinity for target cells [7,18,19], during a preclearing step designed to remove potentially reactive, non-specific components before the TN58-mediated precipitation. Consequently, the binding complex was loaded onto streptavidin-coated magnetic beads and the unbound fraction was recovered and incubated with the truncated 2′F-Py-RNA sequence of TN58, which possesses a biotin tag at the 5′-end (biotin-sTN58); it exhibits binding characteristics identical to the parental moiety, with a Kd value of approximately 10 nM on Cis-Pt-R cells [7]. The binding complex, consisting of biotin-sTN58 and its target, was subsequently isolated utilizing streptavidin-coated magnetic beads. Following extensive washing, the streptavidin beads containing the captured proteins, were subjected to heating in the SDS-PAGE loading buffer, and the eluted proteins were analyzed by SDS-PAGE and visualized by colloidal Coomassie staining (Fig. 1A). The regions of the gel marked by the red boxes, which contain distinct protein bands identified by sTN58, were subjected to digestion and analyzed by LC-MS/MS.

A total of 245 proteins were identified with a minimum of 2 peptides in the sTN58 sample (Table S1). The most suitable protein candidates for further analyses were selected based on the following criteria: 1) a score greater than 40 and sequence coverage exceeding 10 %; 2) a size consistent with the excised gel regions; and 3) classification as cell membrane proteins according to Human Protein Atlas (HPA) and UniProt databases (released February 2024). Seventeen proteins that fulfilled these selection criteria are listed in Table S2. The table lists the gene name, protein name, number of identified peptides, sequence coverage percentage, molecular weight, and score value, with the latter indicating the quality of the peptide spectrum match for each protein [28]. The remaining candidates, being mostly ribonucleoproteins, DNA binding proteins and keratins, were discarded.

After consulting the HPA database, 5 proteins (ephrin type-A receptor 2, CD44, integrin β1, myoferlin, and liprin β1; gene names, EPHA2, CD44, ITGB1, MYOF and PPFIBP1, respectively) were chosen for experimental validation among the 17 candidates (Fig. 1B). The remaining proteins were excluded due to their overexpression in non-TNBC BC cell lines (BT-474, MCF-7 and/or SK-BR-3) or because they exhibited comparable expression levels in both non-TNBC cells and TNBC MES cell lines (BT-549 and MDA-MB-231) (Fig. 1B). In fact, these proteins are improbable candidates for the target of sTN58 [6,7].

### 3.2 Identification of CD44 as the target protein of sTN58 aptamer

To identify the potential target of sTN58 among the five protein candidates, we first conducted a combination of protein expression analysis through immunoblotting and aptamer binding assays across various cancer cell lines. To this aim, we selected TNBC MES BT-549 and MDA-MB-231, along with chemoresistant Dox-R and Cis-Pt-R, as representatives for "positive" cells, which are effectively recognized by sTN58. EC50 calculation of Dox-R and Cis-Pt-R cells exposed for 48 h to Dox and Cis-Pt, respectively, confirmed their increased resistance with respect to parental MDA-MB-231 cells (Fig. S1). Conversely, we used non-TNBC BC BT-474 and MCF-7, as well as epidermoid cancer A431, as "negative" cells, which are not bound by the aptamer [6,7]. Consistently with the HPA database (Fig. 1B) and previous studies, CD44 [29], integrin β1 [30], EphA2 [31], myoferlin [32] and liprin β1 [33] were found expressed at higher levels in TNBC cells compared to non-TNBC BC cells, with CD44 and integrin β1 strongly enriched in chemoresistant cells [10] (Fig. 1C and D). Moreover, in agreement with previous findings [34], MES BT-549 and MDA-MB-231 cells, along with their chemoresistant derivatives, predominantly expressed the mesenchymal standard form of CD44 (CD44s, typically 85–95 kDa) [11,14,35,36]. This form is notably upregulated in advanced human breast tumors exhibiting EMT traits and resistance to therapy [13]. In contrast, these cell lines weakly expressed the CD44 variant (CD44v) isoforms (>100 kDa). This differential expression pattern reinforces the role of CD44s in the progression and treatment resistance of mesenchymal TNBC cells, making it a compelling candidate for further investigation as the target of sTN58.

As an independent validation of our methodology, one protein from the seventeen listed in Table S2, but not among the 5 candidates (Fig. 1B), specifically ZO-1 (also known as TJP1) epithelial cell marker, was expressed in "negative" non-TNBC cells (Fig. 1C and D). Consistent with our previous findings [10], this protein was downregulated in Dox-R and Cis-Pt-R cells (Fig. 1C and D), aligning with their mesenchymal/stem-like phenotype. This observation further supports the robustness of our selection criteria and underscores the potential of the identified proteins as relevant targets in mesenchymal and chemoresistant BC phenotypes.

Among the five proteins analyzed, the expression pattern of CD44 aligns with the cell binding profile of sTN58, as indicated by the apparent Kd values of aptamer-cell interaction (Fig. 1E and Fig. S2). This compatibility supports the hypothesis that CD44 is the target protein for the aptamer.

**Fig. 2:**
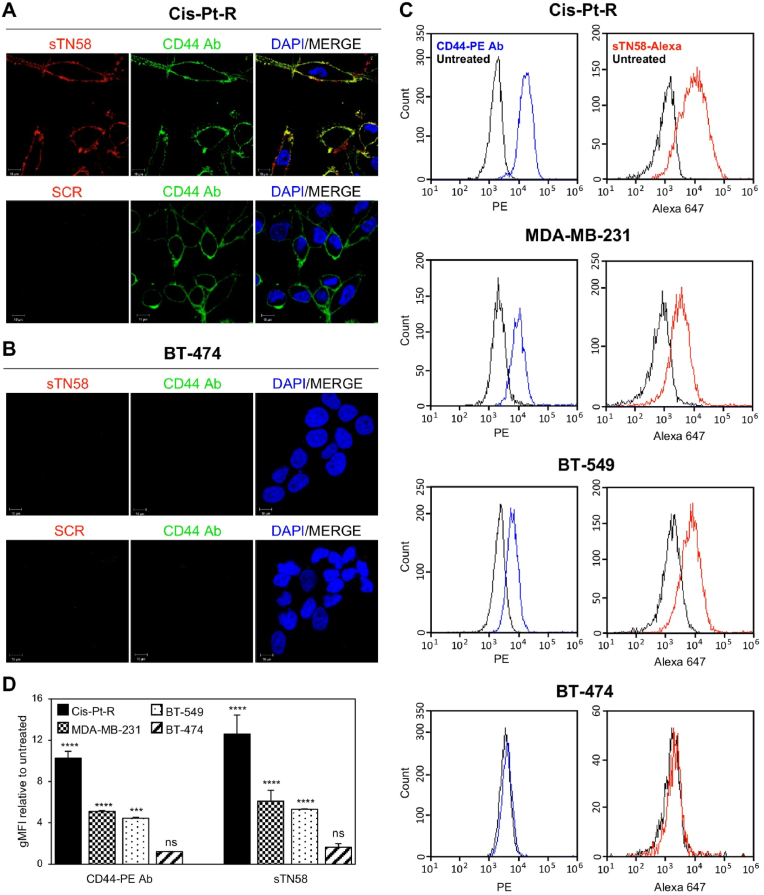
sTN58 binds to CD44-positive TNBC cell lines. Following 5 min incubation at RT with 2 μM Alexa 647-sTN58, Cis-Pt-R (A) or BT-474 (B) cells were stained with CD44 Ab, visualized by confocal microscopy, and photographed. Alexa 647-SCR was used as a negative control. Alexa 647-sTN58, CD44 Ab and nuclei are visualized in red, green, and blue, respectively. Magnification 63×, 1.0× digital zoom, scale bar = 10 μm. Co-localization results appear yellow in the merged images (Overlap Coefficient 0.74). All digital images were captured under identical settings to allow direct comparison of staining patterns. (C) Flow cytometry analyses of Cis-Pt-R, MDA-MB-231, BT-549 and BT-474 cells treated with CD44-PE Ab or Alexa 647-sTN58. (D) Quantification of the geometric mean fluorescence intensity (gMFI) of Alexa 647-sTN58- or CD44-PE Ab-treated cells normalized to the gMFI of the untreated cells. Bars depict mean ± SD of at least two independent experiments. ∗∗∗*P* < 0.001, ∗∗∗∗*P* < 0.0001 relative to untreated cells; ns, no significant.

Thus, as a first step to validate whether sTN58 targets CD44, 5 min-treatment of Cis-Pt-R cells with Alexa 647-labeled sTN58 was combined with immunofluorescence using an antibody raised against CD44 (CD44 Ab), and cells were imaged by confocal microscopy. As shown in Fig. 2A, the fluorescent signals from sTN58 (red) and CD44 Ab (green) colocalized on the cell membrane (yellow in merged images), suggesting that sTN58 targets TNBC cells through CD44. An undetectable signal was obtained with the non-targeting SCR aptamer. Moreover, as expected, neither Alexa 647-sTN58 nor the CD44 Ab bound to CD44-negative BT-474 cells (Fig. 2B). Flow cytometry, including fluorescently labeled antibodies as a control and the use of knockdown of the target protein, is an essential technique for validating the target specificity of cell-targeting aptamers [37]. Importantly, flow cytometry analyses further confirmed the binding of both the aptamer and the CD44-PE Ab to Cis-Pt-R, MDA-MB-231 and BT-549 cells, while no binding was observed to BT-474 cells (Fig. 2C and D). These results align closely with the cell targeting specificity of sTN58 (Fig. 1E) [6,7] and the immunoblotting data for CD44 expression (Fig. 1C and D), providing robust evidence that sTN58 specifically targets CD44-expressing TNBC cells.

**Fig. 3:**
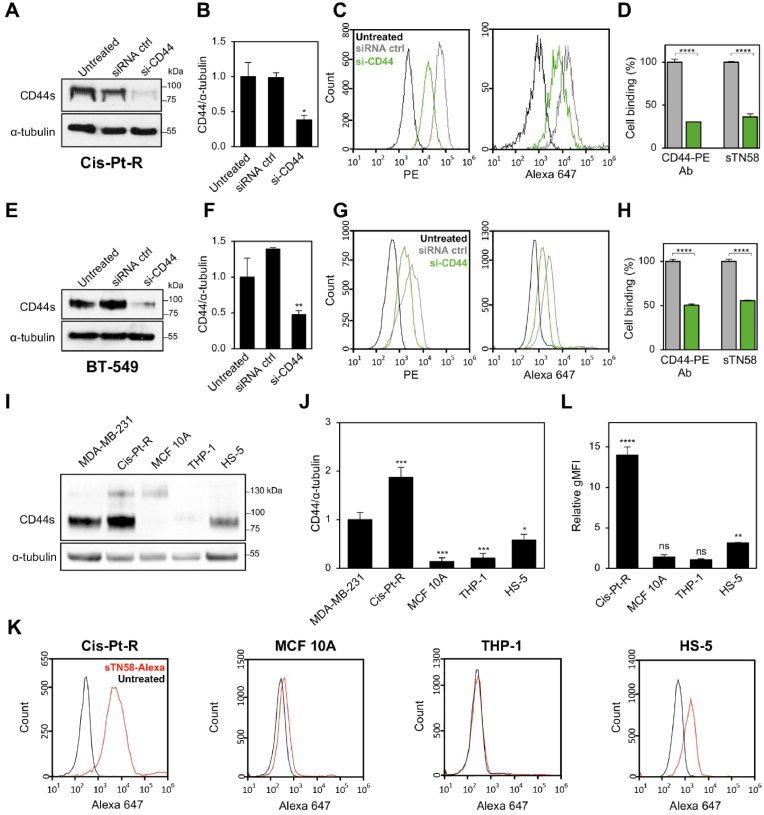
CD44 silencing results in reduced sTN58 binding. (A, E) Cis-Pt-R (A) and BT-549 (E) cells were left untreated or transfected with si-CD44 or siRNA ctrl. At 48 h post-transfection, cells were harvested, and cell lysates prepared and immunoblotted with CD44 Ab. Equal loading was confirmed by immunoblot with anti-α-tubulin antibody. Molecular weights of protein markers are reported. (B, F) The histogram depicts the densitometric ratio of CD44 expression to α-tubulin. Values are shown relative to the untreated control, arbitrarily set to 1. ∗*P* < 0.05, ∗∗*P* < 0.01 relative to siRNA ctrl. (C, G) Binding of CD44-PE Ab (*left*) and Alexa 647-sTN58 (*right*) to Cis-Pt-R (C) and BT-549 (G) cells following 48 h transfection with si-CD44 (green) and siRNA ctrl (gray). (D, H) The histogram shows gMFI of si-CD44-transfected cells treated with sTN58 aptamer or CD44 Ab, normalized to the gMFI of untreated cells, and expressed as percentage with respect to siRNA ctrl-transfected cells. ∗∗∗∗*P* < 0.0001 relative to siRNA ctrl. (I) Immunoblot analysis of CD44 and the housekeeping protein α-tubulin. The molecular weights of protein markers are reported. (J) The histogram shows the relative fold-change in CD44 expression levels compared to α-tubulin, normalized to MDA-MB-231 target cells, whose expression level is arbitrarily set to 1. ∗*P* < 0.05, ∗∗∗*P* < 0.001 relative to MDA-MB-231. (K) Flow cytometry analyses of Cis-Pt-R, MCF 10A, THP-1 and HS-5 cells treated with Alexa 647-sTN58. (L) Quantification of the gMFI of Alexa 647-sTN58-treated cells normalized to the gMFI of the untreated cells. ∗∗*P* < 0.01, ∗∗∗∗*P* < 0.0001 relative to untreated cells; ns, no significant. In B, D, F, H, J, L, bars depict mean ± SD of at least two independent experiments.

Thus, as supplementary evidence, we evaluated the binding of sTN58 to Cis-Pt-R cells that were interfered for CD44 expression by specific siRNA molecules (Fig. 3A and B) and compared it to that of CD44-specific antibody. Notably, CD44 knockdown through si-CD44 treatment led to a significant reduction in sTN58 binding to the cells (approximately 55 % compared to siRNA control cells), a decrease comparable to that observed with CD44-PE (Fig. 3C and D). To further confirm our finding, we examined if CD44 knockdown by siRNA in BT-549 cells (Fig. 3E and F) would decrease sTN58's cell affinity. As expected, a decreased level of cell binding was observed that correlated with the reduced expression of CD44 on the cell surface, as reflected by CD44-PE staining (Fig. 3G and H).

CD44s is overexpressed in mesenchymal stem-like cancer cells, and expressed at various levels in other non-tumorigenic cell types, including stromal fibroblasts and immune cell populations [11]. Therefore, to assess the selectivity of sTN58, we performed a flow cytometry experiment under the same condition previously used (Fig. 2C and D), but in normal breast epithelial MCF 10A cells, HS-5 fibroblasts and THP-1 monocytes (Fig. 3I–L). As shown, sTN58 had no capacity to bind to MCF 10A cells, thus confirming our previous data with the full-length TN58 aptamer [6], and THP-1 cells (Fig. 3K and L), reflecting the absent/low expression of CD44 in these cell lines (Fig. 3I, J and Fig. S3). Conversely, it also binds to HS-5 fibroblasts - albeit to a lesser extent than both parental MDA-MB-231 cells and their chemoresistant derivatives (Fig. 3K and L), - which, consistent with their mesenchymal phenotype, express CD44 (Fig. 3I and J and Fig. S3). Taken together, these findings demonstrate that CD44 is the specific target of sTN58 aptamer.

### 3.3 Alexa-sTN58-based cell imaging reveals CD44/integrin β1 association on chemoresistant TNBC cells

To further validate our exclusion criteria, we performed colocalization analyses by confocal microscopy considering two proteins: kinectin 1, excluded based on cellular localization, and myoferlin, excluded due to the lack of association between sTN58 binding and protein expression. As shown (Fig. S4), distinct staining patterns were observed between Alexa-sTN58 and antibodies against kinectin 1 and myoferlin in Cis-Pt-R cells, indicating that these two proteins are not targets of the aptamer.

**Fig. 4:**
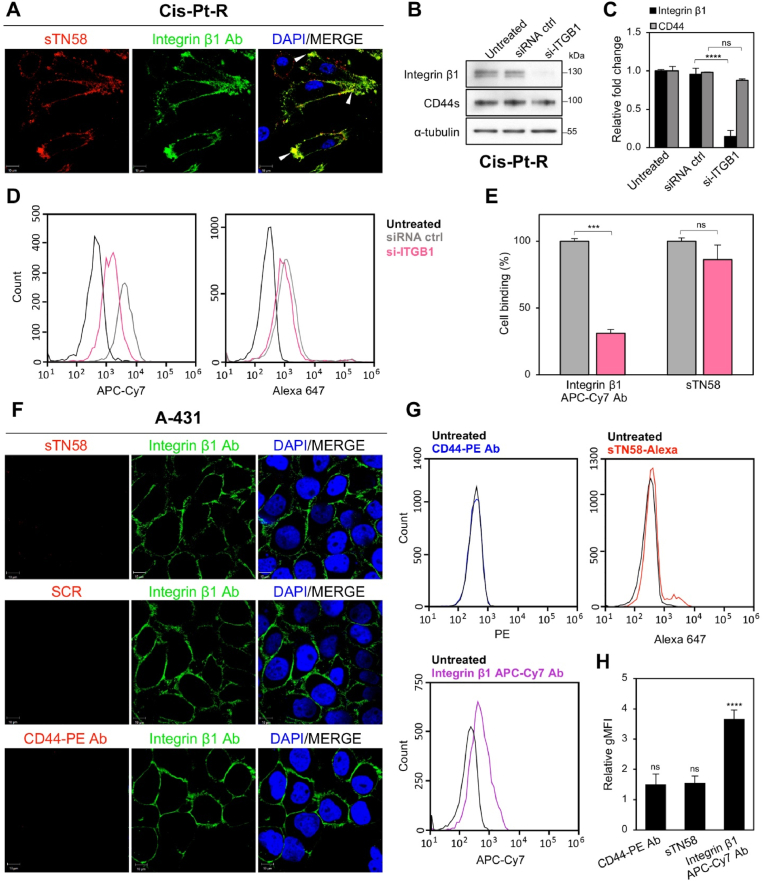
sTN58 aptamer and CD44 Ab colocalize with integrin β1 Ab on Cis-Pt-R cells. (A) Following 5 min incubation at RT with 2 μM Alexa 647-sTN58, Cis-Pt-R cells were stained with integrin β1 Ab, visualized by confocal microscopy, and photographed. (B) Cell lysates from Cis-Pt-R cells left untreated or treated for 48 h with 100 nM siRNA ctrl or si-ITGB1 were analyzed by immunoblotting with integrin β1, CD44 and anti-α-tubulin antibodies. Molecular weights of protein markers are reported. (C) The histogram shows the protein expression/α-tubulin ratio based on the densitometric signals. Values are shown relative to untreated samples, arbitrarily set to 1. (D) Binding of integrin β1-APC-Cy7 Ab (*left*) and Alexa 647-sTN58 (*right*) to Cis-Pt-R cells following 48 h transfection with siRNA ctrl (gray) and si-ITGB1 (pink). (E) The histogram shows gMFI of si-ITGB1-transfected cells treated with Alexa 647-sTN58 or integrin β1-APC-Cy7 Ab, normalized to the gMFI of untreated cells, and expressed as percentage with respect to siRNA ctrl-transfected cells. Bars depict mean ± SD of two independent experiments. ∗∗∗*P* < 0.001; ns, no significant. (F) Confocal microscopy analyses of A431 cells treated with sTN58 and stained with integrin β1 Ab, as in A, or stained with CD44-PE and integrin β1 antibodies. Alexa 647-SCR was used as a negative control. In A, F, aptamers and CD44-PE Ab are visualized in red, integrin β1 Ab in green and nuclei in blue. All digital images were captured at the same setting to allow direct comparison of staining patterns. Magnification 63×, 1.0× digital zoom, scale bar = 10 μm. Co-localization results appear yellow in the merged images. Arrowheads indicate some co-localization points between sTN58 and integrin β1 Ab (Overlap Coefficient, 0.80). (G) Flow cytometry analyses of A431 cells treated with CD44-PE Ab, Alexa 647-sTN58 and integrin β1-APC-Cy7 Ab. (H) Quantification of the gMFI of sTN58-, CD44 PE- and integrin β1-APC-Cy7-treated cells normalized to the gMFI of the untreated cells. Bars depict mean ± SD of three independent experiments. ∗∗∗∗*P* < 0.0001 relative to untreated cells; ns, no significant.

Conversely, a clear colocalization was observed when Cis-Pt-R cells were incubated with Alexa 647-sTN58 aptamer and integrin β1 antibody (integrin β1 Ab) (Fig. 4A, see arrowheads in merged image), indicating that CD44 and integrin β1 are closely associated on the cell surface. Indeed, colocalization spots that closely resemble those observed with sTN58 were identified on Cis-Pt-R cells using CD44-PE Ab and integrin β1 Ab (Fig. S5). Consistently, previous studies have demonstrated that CD44 and integrin β1 are co-enriched and colocalized in plasma membrane microdomains where they cooperate in regulating cancer cell motility [[38], [39], [40], [41]]. Importantly, the binding of sTN58 to Cis-Pt-R cells was unaffected by integrin β1 knockdown (Fig. 4B–E), indicating that sTN58 interacts with CD44 independently of integrin β1. Similar results were observed in MDA-MB-231 cells, where siRNA-mediated integrin β1 knockdown reduced integrin β1 Ab binding but had no effect on sTN58 binding (Fig. S6). We verified that silencing integrin β1 (Fig. 4B and C; Fig. S6A and B; Fig. S7E and F) or CD44 (Fig. S7A–D) does not alter CD44 or integrin β1 expression, respectively. In agreement with these results, sTN58 does not bind to CD44-negative and integrin β1-positive A431 cells (Fig. 1C–E) [6], as evidenced by confocal microscopy (Fig. 4F) and flow cytometry (Fig. 4G and H) analyses, thereby confirming that sTN58 binding to cell surface is dependent upon the presence of CD44.

Overall, these data show the ability of the sTN58 CD44-targeting aptamer to detect the CD44/integrin β1 co-expression, which is associated with the mesenchymal, stem-like, and chemoresistant traits of TNBC cells.

### 3.4 sTN58 aptamer inhibits the invasion capability and HA-dependent tube formation of chemoresistant TNBC cells

**Fig. 5:**
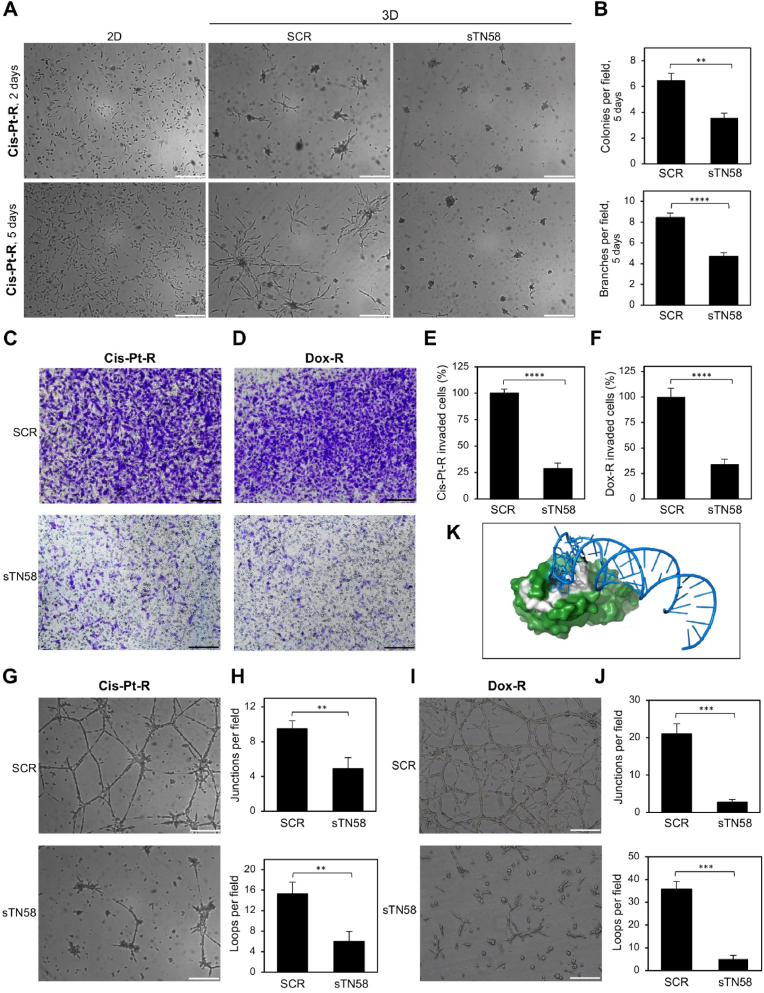
sTN58 inhibits invasive growth and vessel like-structures formation in 3D cell cultures. (A) Representative phase-contrast images of Cis-Pt-R cells grown in 2D or in Matrigel (3D) in the presence of 500 nM sTN58 or SCR for the indicated time points. (B) The invasive ability of Cis-Pt-R cells at 5 days is expressed as the number of colonies and branches per field. (C, D) Invasion of Cis-Pt-R (C) and Dox-R (D) cells toward 10 % FBS was analyzed by transwell invasion assay in the presence of 500 nM sTN58 or SCR for 72 h. Photographs of a representative experiment are shown. (E, F) Data are presented as percentage of invaded cells in the presence of sTN58 compared with SCR control. (G, I) Representative phase-contrast images of Cis-Pt-R cells (G) and Dox-R (I) grown on Matrigel in the presence of HA and treated with 500 nM sTN58 or SCR for 24 h. (H, J) Tube formation ability is expressed as the number of junctions and loops per field. (A, C, D, G, I) Magnification 10×, scale bar = 200 μm. (B, E, F, H, J) Bars depict means ± SEM of at least two independent experiments. ∗∗*P* < 0.01, ∗∗∗*P* < 0.001, ∗∗∗∗*P* < 0.0001 relative to SCR-treated cells. (K) AF3 molecular docking model of sTN58-CD44 binding complex; sTN58, HA-binding site and CD44 folded domain are shown in blue, white and green, respectively.

We have previously shown that sTN58 aptamer drastically inhibits the mammosphere-forming ability of MES-TNBC MDA-MB-231 and BT-549 cells and counteracts the up-regulation of CD44 expression in both cell lines grown in stem-permissive conditions [6,7]. CD44 is known to promote TNBC cell migration and invasion [42], and is associated with tumor metastasis and chemoresistance in TNBC patients [29,43]. To get deeper insight into the anti-tumor functionality of the aptamer, we evaluated its effect on the invasive growth of chemoresistant Cis-Pt-R cells under 3D conditions. To this aim, cells were cultured in Matrigel for up to 5 days in the presence of 500 nM sTN58 or the negative control SCR. As shown in Fig. 5A, after 2 days SCR-treated cells formed stellate structures with branches extending into the Matrigel, a typical mesenchymal phenotype which reflects diminished cell-cell interactions and enhanced cell invasiveness [19,44]. The number and length of the branches increased over time, and by day 5, spider-like projections had formed, bridging multiple cell colonies. Notably, sTN58 treatment resulted in approximately a 50 % reduction in both the number of invasive colony (*P* = 0.0034) and branches (*P* < 0.0001) compared to the SCR negative control (Fig. 5A and B). Accordingly, we found that sTN58 treatment can increase E-cadherin and decrease vimentin levels with respect to SCR treatment, as assessed by immunoblotting analysis on lysates from Cis-Pt cells harvested from Matrigel following 2 days of culture (Fig. S8). We then examined the effect of sTN58 on cell invasion using a transwell invasion assay, which measures the chemotactic ability of invasive cancer cells to migrate through a Matrigel-coated membrane, simulating the extracellular matrix [45]. Consistent with the aptamer's ability to inhibit invasive cell growth in Matrigel, we observed that the invasion rate of Cis-Pt-R cells in the presence of sTN58 treatment was reduced by approximately 70 % at 72 h, compared to SCR-treated cells (Fig. 5C–E). Importantly, similar results were obtained with Dox-R cells, whose invasion was inhibited by approximately 65 % in the presence of sTN58 compared to negative control (Fig. 5D–F), thus confirming the broad applicability of sTN58. These results were not influenced by differences in cell number, as shown by cell viability assays with Cis-Pt-R and Dox-R cells (Fig. S9). Moreover, according to the CD44 role in enhancing tumor cell aggressiveness by promoting stem-like plasticity [46], 24 h treatment of Cis-Pt-R cells with HA (50 μg/mL, 180 kDa) induced the formation of tubule-like structures (Fig. 5G). These structures were significantly affected by sTN58, which caused approximately 50 % (*P* = 0.0088) and 60 % (*P* = 0.0065) reductions in the number of junctions and loops, respectively, compared to SCR (Fig. 5H). Similarly, the inhibitory effect of sTN58 on vessel-like-structures formation was also observed in Dox-R cells (Fig. 5I), where it caused more than 85 % reduction of junctions and loops, compared to SCR (Fig. 5J). This finding suggests that sTN58 and HA share an overlapping binding site on CD44.

To have a better understanding of the interaction between sTN58 and CD44, we led molecular docking analysis using the AlphaFold 3 (AF3) artificial intelligence-based system. AF3 can model protein–nucleic acid interactions at high accuracy [47]. Molecular docking was performed using the AF3 server, where the nucleotide sequence of sTN58 and the amino acid sequence of CD44, adopting a folded conformation based on the Protein Data Bank (PDB) crystallographic structure (PDB ID: 1poz), were run. Interestingly, as shown in Fig. 5K, the potential binding site of sTN58 (blue) on CD44 (green) overlaps with the HA-binding site (white) described by Mahoney DJ et al. [48], suggesting that sTN58 may interfere with HA binding to CD44.

These results confirm that CD44 is a functional binding target of sTN58 in chemoresistant TNBC cells.

### 3.5 In vivo tumor targeting by sTN58 aptamer

**Fig. 6:**
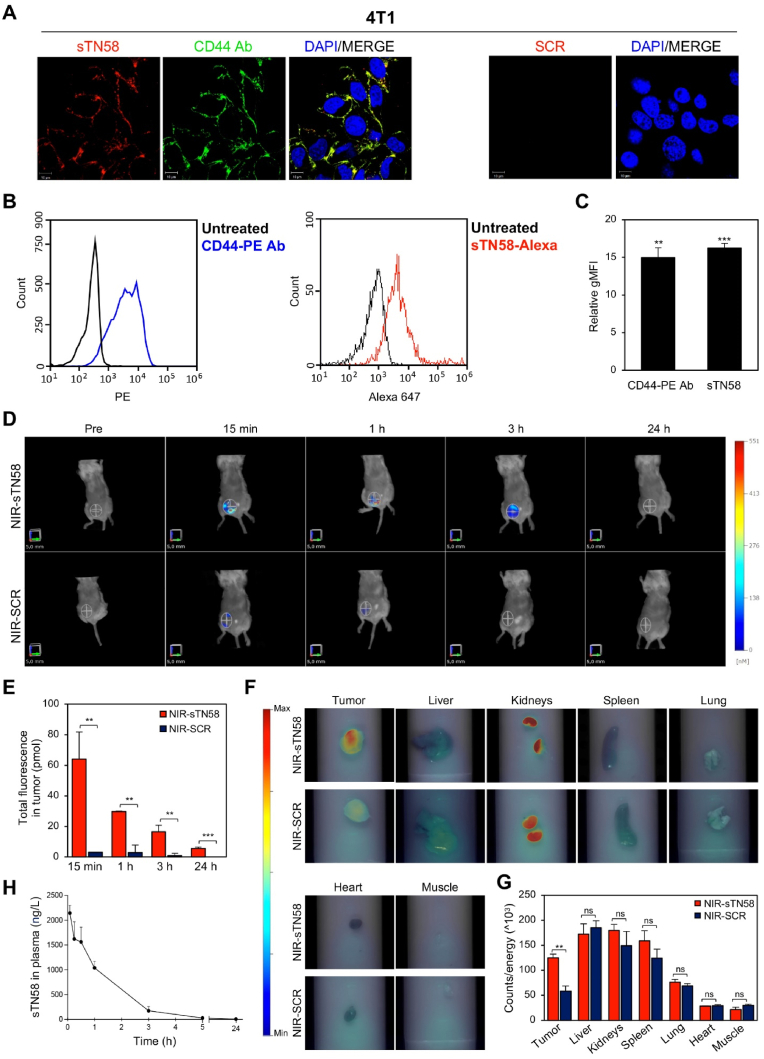
sTN58 selectively targets CD44-positive 4T1 xenografts. (A) Representative confocal images of 4T1 (8.0 × 10^4^ cells) incubated with 2 μM Alexa 647-sTN58 or Alexa 647-SCR and then fixed and stained with CD44 Ab. Aptamers, CD44 Ab and nuclei are visualized in red, green and blue, respectively. Co-localization results appear yellow in the merged images (Overlap Coefficient, 0.76). All digital images were captured at the same setting to allow direct comparison of staining patterns. Magnification 63× , 1.0× digital zoom, scale bar = 10 μm. (B) Binding of CD44-PE Ab (*left*) and Alexa 647-sTN58 (*right*) to 4T1 cells using flow cytometry. (C) Quantification of the gMFI of sTN58 aptamer- or CD44 Ab-treated cells normalized to the gMFI of the untreated cells. Bars depict mean ± SD of two independent experiments. ∗∗*P* < 0.01, ∗∗∗*P* < 0.001 relative to untreated cells. (D) Mice bearing mammary fat pad orthotopic 4T1 tumors were *i.v.* injected with 0.75 nmol of either NIR-sTN58 or NIR-SCR and analyzed with FMT at the indicated time points; Pre, before injection. Representative volume renderings taken at the same color gating for NIR-sTN58 and NIR-SCR injected mice are shown. (E) The amount of fluorescence (pmol) was quantified in specific VOIs encompassing the tumor in the animal. (F) Representative ex vivo FRI imaging of tumor and major organs (liver, kidneys, spleen, lung, heart and muscle) harvested from mice at 24 h post-injection of NIR-sTN58 and NIR-SCR. (G) The histogram indicates the mean FRI Signal Intensity of tumors and organs in the two groups. (E, G) Bars depict mean ± SD. ∗∗*P* < 0.01; ∗∗∗*P* < 0.001 relative to NIR-SCR; ns, no significant. (H) Plasma pharmacokinetic profile of sTN58. Concentration of aptamer is shown as a function of time following a single i.v. injection in Balb/c mice. Data are presented as the mean ± SEM.

In order to test the efficacy of sTN58 in BALB/c mice bearing syngeneic, orthotopic 4T1 murine TNBC, we first assessed the capability of sTN58 to bind to murine 4T1 cells that are positive for CD44 expression [49]. As shown by confocal microscopy (Fig. 6A), an extensive overlap of CD44-PE Ab and Alexa 647-labeled sTN58 fluorescent signals was observed, thus indicating a clear co-localization of the aptamer and the antibody on the receptor expressed on 4T1 cell surface. No binding of SCR to the cells was observed. Accordingly, flow cytometry experiments confirmed the efficient binding of sTN58 to 4T1 cells (Fig. 6B and C).

Before moving to the in vivo experiments, we verified the high nuclease resistance of sTN58, due to the presence of 2′F-Pys in the RNA sequence, by incubating it at 37 °C in 80 % human serum for increasing times. Consistent with our previous findings [7], sTN58 was stable at up to 8 h and then slowly degraded, with an estimated half-life of approximately 47 h (Fig. S10), thus allowing further investigation in mice. To elucidate the sTN58 tumor-targeting and biodistribution characteristics, sTN58 and the negative control SCR, each containing an amino group at the 5′end, were labeled with the amine-reactive fluorescent NIR dye VivoTag-S 750. As we previously reported, NIR-VivoTag fluorescent probes, which are suitable for deep tissue imaging with minimal autofluorescence interference, failed to visualize tumors and lung metastases when administrated alone to mice bearing xenografts [19,50]. NIR-sTN58 or NIR-SCR (0.75 nmol, approximately 0.55 mg aptamer/kg mean body-weight) were administered intravenously through the tail vein into mice bearing orthotopic 4T1 tumors. Non-invasive imaging was performed over 24 h by using FMT (Fig. 6D), which allowed quantification of the total fluorescence in the tumor VOI. Notably, 15 min post-injection, the fluorescence signal of NIR-sTN58, but not NIR-SCR, increased at the tumor sites, indicating rapid accumulation of the aptamer in the tumors (∼6 % of the injected dose at 15 min) (Fig. 6D and E). Subsequently, the amount of NIR-sTN58 signal steadily decreased over time, reaching its lowest point at 24 h (Fig. 6D and E). However, ex vivo FRI imaging of tumors harvested 24 h post-injections allowed to detect a stronger signal enhancement in tumors from mice injected with NIR-sTN58 compared to those treated with NIR-SCR, indicating durable tumor retention of sTN58 (Fig. 6F and G). As shown, a similar pattern of biodistribution of NIR-sTN58 and NIR-SCR aptamers was observed in major tissues, with greater accumulation in kidneys, liver and spleen and less in heart and muscle, as expected [19,51].

Further, the plasma pharmacokinetic profile of sTN58 (Fig. 6H) following a single i.v. injection in non-tumor-bearing Balb/c mice revealed an estimated circulating half-life of approximately 50 min, as expected from the absence of bulky moiety conjugated to the small-size aptamer [52,53].

These results demonstrate that the sTN58 aptamer has the ability to specifically target TNBC in vivo.

### 3.6 In vivo antitumor efficacy of sTN58 aptamer

**Fig. 7:**
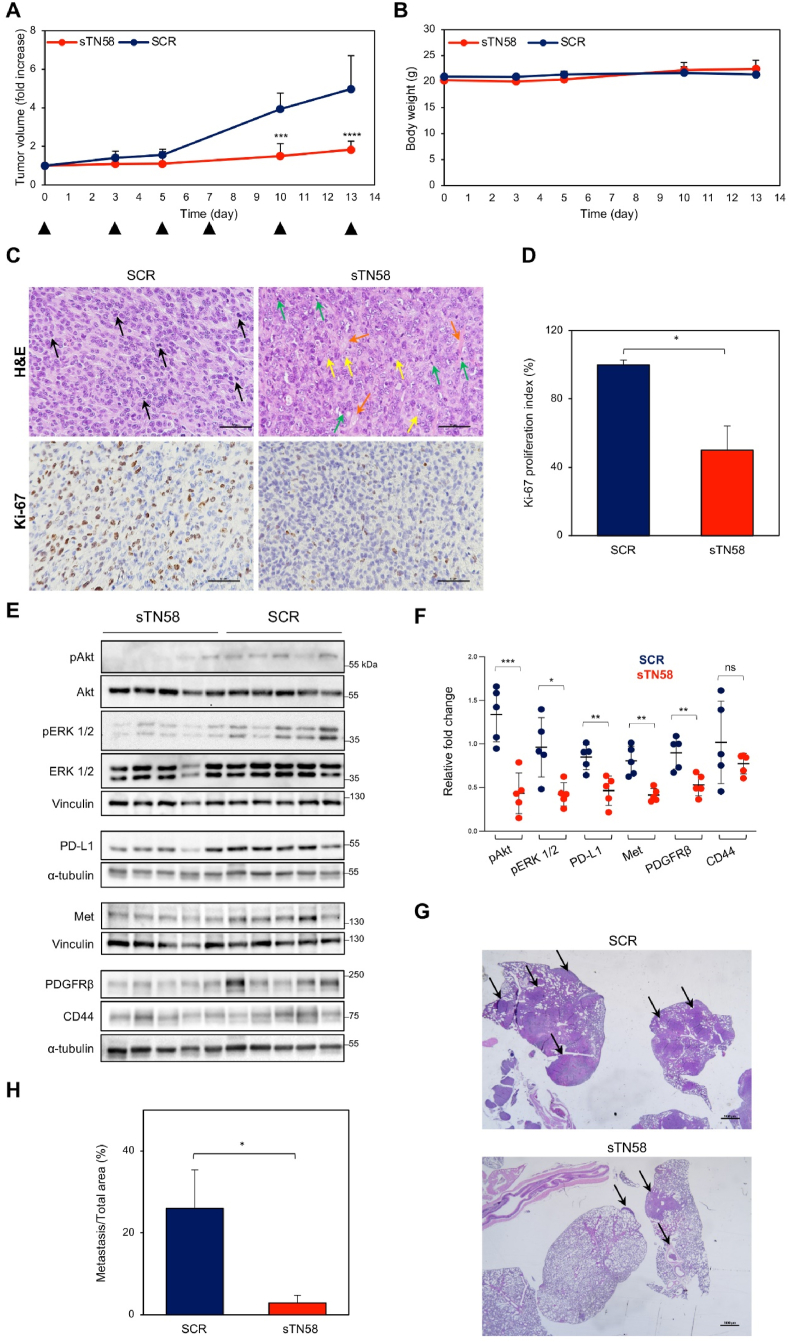
Effect of sTN58 treatment on tumor growth and lung metastases formation. (A) Mice bearing mammary fat pad orthotopic 4T1 tumors were *i.v.* injected with sTN58 or SCR aptamer (at day 0, 3, 5, 10 and 13, indicated by arrowheads). Tumor growth was monitored by calipers over time and experimental raw data (expressed as fold increase) were interpolated with no curve fitting or regression analysis. Day 0 marks the start of treatments. (B) Mice body weight was measured at the indicated days and the mean weight of each group is shown. (A, B) The mean ± SD (*n* = 5) was calculated for all the groups. ∗∗∗*P* < 0.001, ∗∗∗∗*P* < 0.0001 relative to SCR. (C) Shown are images from one representative tumor sample for each treatment group stained for H&E (*upper panels)* or with Ki-67 antibody (*lower panels*). Arrows identify features of cancer cells, as described in the text. Magnification 40×, scale bar = 50 μm. (D) Ki-67 proliferation index was calculated as percentage of Ki-67 positive cells/total cell count for randomly selected 40× microscopic fields considering the SCR-group as 100 %. Bars depict mean ± SD. (E) Lysates from recovered tumors were immunoblotted with the indicated antibodies. Equal loading was confirmed by immunoblot with anti-vinculin or anti-α-tubulin antibody. Molecular weights of protein markers are reported. (F) The histogram shows the relative fold of expression of the indicated proteins against the housekeeping protein α-tubulin or vinculin. Each data point represents the sample from an individual mouse (n = 5). (G) Shown are images from one representative lung sample for each treatment group stained for H&E. Magnification 2×; scale bar = 1000 μm. Arrows point to metastasis of breast cancer in the lung. (H) The histogram shows the ratio between the metastasis area and the entire lung tissue section, expressed in percentage. Bars depict mean ± SEM (n = 5). (A, D, F, H) ∗*P* < 0.05, ∗∗*P* < 0.01, ∗∗∗*P* < 0.001, ∗∗∗∗*P* < 0.0001 relative to SCR.

The therapeutic efficacy of the sTN58 aptamer was evaluated in a 4T1-Balb/c orthotopic BC mouse model 17 days after 4T1 cell inoculation, and tumor growth was monitored by a caliper measuring tumor size for further 13 days. Animals were divided into 2 groups (n = 5), receiving intravenous administration of SCR, used as control, or sTN58 at a dose of 1 nmol (approximately 0.74 mg aptamer/kg mean body-weight), at day 0, 3, 5, 7 and 10. From day 10 onward, the difference in tumor growth between SCR and sTN58 treatments reached statistical significance (412.3 ± 153 vs 1134.5 ± 208 mm^3^, sTN58 vs SCR, ∗∗∗∗*P* < 0.0001) with a marked inhibition of tumor growth observed in mice treated with sTN58 compared to the SCR control group (Fig. 7A). Importantly, body weight remained unaffected by the treatments, indicating the absence of systemic toxicity from the aptamer (Fig. 7B). Histopathological features of the tumors, as revealed by H&E staining (Fig. 7C, upper panels), indicated that in the SCR group, tumors were composed predominantly of small to medium-sized neoplastic cells, often arranged in rows with scant eosinophilic cytoplasm. Additionally, there was a frequent occurrence of multinucleated cells exhibiting eccentric nuclei and vacuolated cytoplasm, along with many prominent nucleoli (black arrows), features that correlate with a high proliferative index as confirmed by the Ki-67 staining (Fig. 7C, left lower panel, and 7D). In contrast, neoplastic cells in the sTN58 group displayed a morphological shift toward medium to large size, distinguished by abundant and polymorphic eosinophilic cytoplasm (Fig. 7C, right upper panel, yellow arrows). The nuclei may appear less crowded and, in some fields, there can be signs of lower tumor cell density or possible areas of altered tumor architecture, e.g., smaller, more uniform nuclei or slightly more stromal presence (orange arrows). Notably, this group also exhibited reduced pleomorphism and morphological features consistent with apoptosis, including cell shrinkage and nuclear fragmentation (green arrows), along with decreased Ki-67 labeling index (Fig. 7C, right lower panel, and 7D), indicative of diminished cellular proliferation. Next, accordingly to the strong inhibition of tumor growth, a significant reduction in phosphorylated ERK 1/2 and Akt – key signaling molecules downstream of CD44 [54] – was observed in the tumors of mice treated with sTN58 (Fig. 7E and F). Consistently with in vivo findings, treatment of Cis-Pt-R cells with sTN58, but not SCR, efficiently inhibits ERK 1/2 and Akt activation (Fig. S11). Notably, a substantial downregulation of PDGFRβ, PD-L1 and Met receptor was observed following sTN58 treatment (Fig. 7E and F). This finding aligns with the established correlation of PDGFRβ and PD-L1 with EMT [6,19,55,56], and previous evidence showing that CD44 acts as a key positive regulator of Met [57] expression in aggressive cancers, including TNBC. No significant change of CD44 expression was observed upon sTN58 treatment (Fig. 7E and F).

The 4T1 tumor exhibits a highly metastatic nature, with lungs being the primary target organ [58,59]. Accordingly, lungs from mice bearing orthotopic breast tumors were collected at the end of the aptamer treatments and subjected to histological analysis. Notably, an obvious difference was observed between the H&E-stained lung sections of the SCR-treated group and the sTN58-treated group (Fig. 7G). In the control group, large metastases were evident, involving and replacing up to 50 % of the healthy lung tissue (Fig. 7I). In contrast, the lungs from the sTN58-treated group exhibited only a few isolated neoplastic cells or small peripheral clusters of neoplastic cells, involving less than 10 % of the lung tissue (Fig. 7I), highlighting the aptamer's efficacy in inhibiting lung metastases in this model.

Altogether, these results confirm the cell culture findings and corroborate that CD44 is a functional binding target of sTN58 in TNBC.

## 4 Discussion

In this study, we identified CD44 as the target of the sTN58 2′F-Py-RNA aptamer, which we originally developed through cell-SELEX against mesenchymal stem-like TNBC cells [6] and subsequently shortened from 84 to 45 nucleotides, maintaining its affinity and specificity toward chemoresistant TNBC cell lines [7,9]. Also, we reported the in vitro and in vivo characterization of the aptamer, thus providing the evidence that sTN58 may be useful for the diagnosis and targeted therapy of CD44-positive TNBC.

The high relevance of oligonucleotide aptamers as first-in-class bioreagents for active cancer targeting is widely recognized, as they have target recognition similar to that of antibodies, but have better tissue penetration, low/absent immunogenicity, higher stability, longer shelf life, and are easy and cheap to produce and scale up without batch-to-batch variations [[60], [61], [62]]. Notably, aptamers targeting cancer cell surface proteins have the potential to serve as therapeutic agents, either as stand-alone antagonists or as delivery vehicles for secondary therapeutics to tumors [4,5,63]. In the first modality, usually the binding of the aptamer to the protein target interferes with its correct functioning by blocking receptor activation induced by natural ligands [27], or its engagement in functional molecular complexes with other cell-surface proteins or component of the TME that are essential in driving cancer growth and progression [26]. In the second case, they are conjugated to the drug or different drug-loaded nano-formulations and act as tumor specific targeting agents [64]. Moreover, beside their use for assessing innovative targeted anticancer strategies, aptamers generated by the SELEX method applied to a specific cell type, with no prior knowledge of cell-surface marker proteins, may lead to the discovery of new actionable biomarkers [4,5].

In this study, we highlight the potential of cell-SELEX for increasing the repertoire, which is currently extremely scarce, of therapeutic targets for chemoresistant TNBC [10,65]. We used sTN58 as a bait in aptamer-mediated purification of cell membrane proteins of cisplatin-resistant TNBC MDA-MB-231 cells, which we generated through chronic exposure of MDA-MB-231 cells to the chemotherapeutic agent. The proteins bound to the aptamer were eluted, subjected to SDS-PAGE and analyzed by LC-MS/MS. Through a filtering method that considers their cellular expression profiles and aptamer's cellular targeting affinity, we recognized CD44 as the target of sTN58 among the proteins detected by LC-MS/MS. Aptamer binding, analyzed using flow cytometry and confocal microscopy on several cell lines expressing CD44 at different extent, and CD44-overexpressing chemoresistant cells, proved that sTN58 selectively binds to CD44; consistently, RNA interference-induced CD44 knockdown disrupts sTN58 binding.

CD44 is a nonkinase transmembrane receptor upregulated in mesenchymal subpopulations of cancer cells and recognized as a molecular marker for CSCs [66,67]. Its involvement in the regulation of several intracellular signaling pathway associated with cell proliferation, adhesion, survival, EMT, invasion and drug resistance [11,54] makes it an attractive target for cancer therapy. CD44 shows alternative splice variants that play a role in cancer development and progression [11]. The shift in the expression from variant isoforms (CD44v) to the standard isoform (CD44s) has been reported as an essential event during the development and progression of recurrent mesenchymal type of human cancers, including BC [13]. Accordingly, the mesenchymal CD44s isoform is mainly upregulated in advanced human breast tumors expressing elevated levels of mesenchymal markers [13]. In TNBC, CD44 overexpression is associated with a more aggressive tumor phenotype [68,69], where it acts as a key regulator of proliferation, invasion, chemoresistance, and tumor relapse.

Several strategies, including CD44-targeting monoclonal antibodies and inhibitory peptides, are in various phases of preclinical and clinical development to deal with chemoresistance, tumor regrowth and metastases of CD44-overexpressing malignancies [11,54,70]. Furthermore, some anti-CD44 aptamers have been developed, utilizing the human recombinant CD44 protein as the target for selection, including 2′FPy RNA Apt1 aptamer [71] and DNA thioaptamers [72]. Even if their antitumor activity in an unconjugated form has not been fully exploited, they have been successfully used as tumor targeting agents in innovative targeted drug delivery approaches both in vitro and in animal models of human cancers, including TNBC [[73], [74], [75]]. Moreover, DNA aptamers generated through SELEX against hepatocellular carcinoma (HCC) cells overexpressing the CD44E and CD44s variants [76], and against CD44 overexpressing CHO-K1 cells [77], have been utilized for delivering 5-Fluoruracil in a HCC xenograft model and for detecting circulating tumor cells in peripheral blood, respectively.

In our current study, the identification of CD44s as the target of sTN58 aptamer on chemoresistant TNBC cells, prompted us to exploit it as an antagonistic agent. Tumorigenicity assays demonstrated that sTN58 significantly inhibits the invasion of both chemoresistant Cis-Pt-R and Dox-R cells. Moreover, it prevents the formation of vessel-like structures induced by HA treatment in these cells, with HA being the primary ligand of CD44. These cell-based experiments, along with molecular docking analysis, suggest that sTN58 and HA may compete for the same binding site on CD44. However, further studies are required to identify the precise binding interface between sTN58 and CD44.

The high stability of sTN58 in serum, due to the modification of all pyrimidines with 2′F-Pys, enabled efficient homing of NIR-labeled sTN58 to CD44-positive tumors following intravenous injection in mice bearing 4T1-derived orthotopic xenografts. In agreement with the efficient tumor penetration proper of small size aptamers [62], sTN58 rapidly targets the tumor and exhibits high tumor retention, which reflects its high binding affinity for target cells and efficient cellular uptake [6,7]. Small aptamers are easily cleared by the kidneys; accordingly, sTN58 exhibits a short circulating half-life in mice (approximately 50 min). From a clinical perspective, this could help to minimize side effects, as the aptamer is quickly excreted if it does not reach the tumor. In this regard, CD44s is overexpressed in mesenchymal stem-like cancer cells, but it is also expressed at various levels in non-cancer cell types [11], which could present a clinical challenge for anti-CD44 therapies due to a potential on-target toxicities in healthy tissues. Our biodistribution studies revealed no accumulation of NIR-sTN58 in healthy organs (e.g. heart and muscle) thus, we expect that sTN58 cytotoxicity will be mostly confined to CD44-overexpressing cancer cells, similar to what occurs with most therapeutic agents targeting proteins that are mainly, but not exclusively, expressed in tumors. Furthermore, evidence have been provided that CD44 exists in two different states, which differ in HA-binding affinity, i.e, active on human breast cancer cells, and inactive on normal cells, and it may shift from inactive to active conformation upon appropriate stimuli within the TME [78]. It can be speculated that factors such as conformation, expression level, distribution, and interactions with other receptors on cancer cells within the TME, may play a crucial role in selectively targeting cancer cells, thereby minimizing nonspecific toxicity to CD44-positive normal cells. However, further investigation using humanized mouse models engrafted with patient-derived xenografts is needed to evaluate this possibility. Notably, in agreement with in vitro results, sTN58 strongly inhibited tumor growth and lung metastases. We found a significant downregulation of phosphorylated ERK1/2 and Akt in tumor samples, providing a mechanistic link to key pathways downstream of CD44 [13,54]. Interestingly, we also observed a decrease in PD-L1 and PDGFRβ expression in tumors from mice treated with sTN58 compared to the control group. In line with the established correlation between PD-L1 expression and EMT in TNBC [6,55,56] and the role of PDGFRβ as a TNBC mesenchymal/stem cell marker [19,23], the reduction of their expression may reflect a decrease in the mesenchymal and stem-like features of 4T1-derived tumors induced by sTN58 treatment. Further highlighting the pleiotropic role of CD44 in regulating different receptors, including Met, to promote cell plasticity and tumor growth [70,79], targeting CD44 with sTN58 led to a downregulation of the Met receptor.

Another key feature of the anti-CD44 sTN58 aptamer is its ability to rapidly internalize into target TNBC cells [6,7], acting as an efficient targeting moiety for delivering doped conjugated polymer nanoparticles to chemoresistant TNBC cells, thereby driving their photo-eradication [9]. The availability of sTN58, in conjunction with highly effective anti-EGFR and anti-PDGFRβ aptamers, which we have validated for TNBC targeting and recently used to generate bispecific aptamer-decorated, light-triggered nanoparticles targeting both tumor and stromal cells [18], offers promising potential for developing multi-targeted therapeutic nanosystems. Such aptamer-armed nanosystems may improve the outcome of heterogeneous cancer [80,81], including recurrent and metastatic TNBC that survive conventional chemotherapy.

With advancements in nucleic acid chemistry and technological methodologies, numerous challenges that have long hindered the practical application of therapeutic aptamers are now being effectively addressed (e.g., optimization of therapeutic efficacy, nuclease resistance, safety profiles, etc.). However, certain limitations remain, such as aptamers rapid clearance by filtering organs and short circulating half-lives within patients, which continue to restrict their clinical applicability [4]. Further studies are needed to optimize aptamers for their effective application in cancer therapy. In this regard, the therapeutic potential of sTN58 alone strongly supports its further characterization. This will be crucial to assess the need to extend its in vivo half-life without compromising tumor penetration, as well as to evaluate its long-term efficacy and potential toxicity, which are critical for translating our preliminary preclinical findings into clinical applications. From a diagnostic point of view, we demonstrated that TN58 can distinguish TNBC subtypes when used for histochemical staining of a TNBC tissue microarray, including 18 TNBC specimens with distinct clinicopathological features [6]. Conversely, TN58 did not bind to TPBC or normal breast tissue [6]. Building on these findings and the data presented in this study, future research will focus on investigating a larger cohort of tumors and correlating CD44 expression with clinical features. This analysis will help to better define the aptamer's binding profile in the context of TNBC heterogeneity.

In conclusion, our results may pave the way for more effective, precision-based therapies that can overcome the limitations of current treatments for aggressive, chemoresistant CD44-overexpressing tumors.

## CRediT authorship contribution statement

**Alessandra Caliendo:** Writing – review & editing, Visualization, Validation, Methodology, Investigation, Formal analysis, Data curation. **Simona Camorani:** Validation, Investigation, Formal analysis, Data curation. **Luis Exequiel Ibarra:** Writing – review & editing. **Gabriella Pinto:** Methodology, Investigation, Formal analysis, Data curation. **Lisa Agnello:** Methodology. **Sandra Albanese:** Methodology, Investigation, Formal analysis. **Antonietta Caianiello:** Methodology. **Anna Illiano:** Methodology. **Rosaria Festa:** Methodology. **Vincenzo Ambrosio:** Methodology. **Giosuè Scognamiglio:** Visualization, Methodology, Investigation, Formal analysis, Data curation. **Monica Cantile:** Visualization, Investigation, Formal analysis, Data curation. **Angela Amoresano:** Visualization, Investigation, Formal analysis, Data curation. **Monica Fedele:** Writing – review & editing, Data curation. **Antonella Zannetti:** Writing – review & editing, Data curation. **Laura Cerchia:** Writing – review & editing, Writing – original draft, Supervision, Resources, Project administration, Funding acquisition, Data curation, Conceptualization.

## Ethics approval and consent to participate

All experimental procedures complied with the European Communities Council directives (2010/63/EU) and the present study was approved by the Italian Ministry of Health (authorization number 932/2018-PR).

## Declaration of competing interest

The authors declare the following financial interests/personal relationships which may be considered as potential competing interests Laura Cerchia reports financial support was provided by AIRC Italian Foundation for Cancer Research. If there are other authors, they declare that they have no known competing financial interests or personal relationships that could have appeared to influence the work reported in this paper.
