# Supplementary material for: A novel CD44-targeting aptamer recognizes chemoresistant mesenchymal stem-like TNBC cells and inhibits tumor growth
Source: Bioact Mater. 2025 Apr 25;50:443–60. doi: 10.1016/j.bioactmat.2025.04.027 (PMC12059597; doi:10.1016/j.bioactmat.2025.04.027)
Supplement: Multimedia component 1 [file mmc1.pdf]

SUPPLEMENTARY MATERIALS

A novel CD44-targeting aptamer recognizes chemoresistant mesenchymal stem-like TNBC cells and inhibits tumor growth

Table S1. List of proteins identified in sTN58 sample by LC-MS/MS analysis. A dataset of 245 proteins was reported considering for each protein the corresponding Uniprot ID, Gene name, Protein name, Intensity, the number of identified peptides, the sequence coverage (%), the molecular weight (kDa) and the identification score.

Fasta headers	Uniprot id	Gene name	Protein name	Intensity sTN58	Peptides	Sequence coverage [%]	Mol. weight [kDa]	Score
sp|P35579|MYH9_HUMAN Myosin-9 OS=Homo sapiens OX=9606 GN=MYH9 PE=1 SV=4	P35579	MYH9	Myosin-9	13072014898	156	65.7	226.53	323.31
sp|P04264|K2C1_HUMAN Keratin, type II cytoskeletal 1 OS=Homo sapiens OX=9606 GN=KRT1 PE=1 SV=6	P04264	KRT1	Keratin, type II cytoskeletal 1	10576003667	64	66.1	66.038	323.31
sp|P13645|K1C10_HUMAN Keratin, type I cytoskeletal 10 OS=Homo sapiens OX=9606 GN=KRT10 PE=1 SV=6;tr|A0A1B0GVI3|A0A1B0GVI3_HUMAN Keratin, type I cytoskeletal 10 OS=Homo sapiens OX=9606 GN=KRT10 PE=1 SV=2	P13645	KRT10	Keratin, type I cytoskeletal 10	5934299786	41	68	58.826	323.31
sp|Q00839|HNRPU_HUMAN Heterogeneous nuclear ribonucleoprotein U OS=Homo sapiens OX=9606 GN=HNRNPU PE=1 SV=6	Q00839	HNRNPU	Heterogeneous nuclear ribonucleoprotein U	5903296869	53	41	90.583	323.31
sp|P46940|IQGA1_HUMAN Ras GTPase-activating-like protein IQGAP1 OS=Homo sapiens OX=9606 GN=IQGAP1 PE=1 SV=1;tr|A0A0J9YXZ5|A0A0J9YXZ5_HUMAN Ras GTPase-activating-like protein IQGAP1 OS=Homo sapiens OX=9606 GN=IQGAP1	P46940	IQGAP1	Ras GTPase-activating-like protein IQGAP1	4509105891	87	55.8	189.25	323.31

PE=1 SV=1;tr|H0YLE8|H0YLE8_HUMAN Ras GTPa								
sp|P07814|SYEP_HUMAN Bifunctional glutamate/proline--tRNA ligase OS=Homo sapiens OX=9606 GN=EPRS1 PE=1 SV=5;tr|V9GYZ6|V9GYZ6_HUMAN Glutamyl-tRNA synthetase (Fragment) OS=Homo sapiens OX=9606 GN=EPRS1 PE=1 SV=1	P07814	EPRS1	Bifunctional glutamate/proline--tRNA ligase	3605601792	75	48.9	170.59	323.31
sp|Q15149|PLEC_HUMAN Plectin OS=Homo sapiens OX=9606 GN=PLEC PE=1 SV=3;tr|H0YDN1|H0YDN1_HUMAN Plectin OS=Homo sapiens OX=9606 GN=PLEC PE=1 SV=9	Q15149	PLEC	Plectin	3121798063	275	55.8	531.78	323.31
sp|P41252|SYIC_HUMAN Isoleucine--tRNA ligase, cytoplasmic OS=Homo sapiens OX=9606 GN=IARS1 PE=1 SV=2;tr|A0A804HJN6|A0A804HJN6_HUMAN Isoleucine--tRNA ligase, cytoplasmic OS=Homo sapiens OX=9606 GN=IARS1 PE=4 SV=1;tr|A0A0A0MSX9|A0A0A0MSX9_HUMAN Isoleucyl-tRN	P41252	IARS1	Isoleucine--tRNA ligase, cytoplasmic	2824300608	59	48.5	144.5	323.31
sp|P35527|K1C9_HUMAN Keratin, type I cytoskeletal 9 OS=Homo sapiens OX=9606 GN=KRT9 PE=1 SV=3;tr|K7EQQ3|K7EQQ3_HUMAN Keratin, type I cytoskeletal 9 OS=Homo sapiens OX=9606 GN=KRT9 PE=1 SV=1	P35527	KRT9	Keratin, type I cytoskeletal 9	2707901225	37	60	62.064	323.31
sp|P35908|K22E_HUMAN Keratin, type II cytoskeletal 2 epidermal OS=Homo sapiens OX=9606 GN=KRT2 PE=1 SV=2	P35908	KRT2	Keratin, type II cytoskeletal 2 epidermal	2674701039	48	72.1	65.432	323.31
sp|Q08211|DHX9_HUMAN ATP-dependent RNA helicase A OS=Homo sapiens OX=9606 GN=DHX9 PE=1 SV=4	Q08211	DHX9	ATP-dependent	1602999587	41	35.4	140.96	323.31

			RNA helicase A					
sp|Q7Z478|DHX29_HUMAN ATP-dependent RNA helicase DHX29 OS=Homo sapiens OX=9606 GN=DHX29 PE=1 SV=2;tr|A0A087WYN9|A0A087WYN9_HUMAN ATP-dependent RNA helicase DHX29 OS=Homo sapiens OX=9606 GN=DHX29 PE=1 SV=1	Q7Z478	DHX29	ATP-dependent RNA helicase DHX29	824989529.1	51	40.5	155.23	323.31
sp|Q9Y5B9|SP16H_HUMAN FACT complex subunit SPT16 OS=Homo sapiens OX=9606 GN=SUPT16H PE=1 SV=1	Q9Y5B9	SUPT16H	FACT complex subunit SPT16	793509920.8	36	35.6	119.91	323.31
sp|P53621|COPA_HUMAN Coatomer subunit alpha OS=Homo sapiens OX=9606 GN=COPA PE=1 SV=2;tr|A0A3B3IT15|A0A3B3IT15_HUMAN Coatomer subunit alpha OS=Homo sapiens OX=9606 GN=COPA PE=1 SV=1;tr|A0A3B3IS84|A0A3B3IS84_HUMAN Coatomer subunit alpha OS=Homo sapiens OX=9	P53621	COPA	Coatomer subunit alpha	514999831.5	41	36.5	138.34	323.31
tr|A0A6I8PIV1|A0A6I8PIV1_HUMAN Leucyl-tRNA synthetase OS=Homo sapiens OX=9606 GN=LARS1 PE=1 SV=1;sp|Q9P2J5|SYLC_HUMAN Leucine--tRNA ligase, cytoplasmic OS=Homo sapiens OX=9606 GN=LARS1 PE=1 SV=2;tr|A0A6I8PRS0|A0A6I8PRS0_HUMAN Leucyl-tRNA synthetase OS=Homo	A0A6I8PIV1	LARS1	Leucyl-tRNA synthetase	384270157.6	37	35.9	134.34	323.31
tr|A0A024R4E5|A0A024R4E5_HUMAN High density lipoprotein binding protein (Vigilin), isoform CRA_a OS=Homo sapiens OX=9606 GN=HDLBP PE=1 SV=1;sp|Q00341|VIGLN_HUMAN Vigilin	A0A024R4E5	HDLBP	High density lipoprotein binding protein (Vigilin), isoform CRA	377949939.9	48	40.5	141.44	323.31

OS=Homo sapiens OX=9606 GN=HDLBP PE=1 SV=2;tr|H0Y394|H0Y394_HUMAN Vigilin (Fragment) O								
sp|Q86UP2|KTN1_HUMAN Kinectin OS=Homo sapiens OX=9606 GN=KTN1 PE=1 SV=1;tr|G3V4Y7|G3V4Y7_HUMAN Kinectin OS=Homo sapiens OX=9606 GN=KTN1 PE=1 SV=1	Q86UP2	KTN1	Kinectin	197300127.9	44	33.2	156.27	323.31
sp|P33176|KINH_HUMAN Kinesin-1 heavy chain OS=Homo sapiens OX=9606 GN=KIF5B PE=1 SV=1	P33176	KIF5B	Kinesin-1 heavy chain	187030059.2	37	46.3	109.68	323.31
tr|A0A804HIN4|A0A804HIN4_HUMAN Leucine-rich PPR motif-containing protein, mitochondrial OS=Homo sapiens OX=9606 GN=LRPPRC PE=4 SV=1;tr|A0A804HK30|A0A804HK30_HUMAN Leucine-rich PPR motif-containing protein, mitochondrial OS=Homo sapiens OX=9606 GN=LRPPRC PE	A0A804HIN4	LRPPRC	Leucine-rich PPR motif-containing protein, mitochondrial	108459929.7	53	39.4	159.09	323.31
tr|A0A7P0T9C4|A0A7P0T9C4_HUMAN Cytoplasmic dynein 1 heavy chain 1 OS=Homo sapiens OX=9606 GN=DYNC1H1 PE=1 SV=1;sp|Q14204|DYHC1_HUMAN Cytoplasmic dynein 1 heavy chain 1 OS=Homo sapiens OX=9606 GN=DYNC1H1 PE=1 SV=5;tr|A0A7P0TA13|A0A7P0TA13_HUMAN Cytoplasmic	A0A7P0T9C4	DYNC1H1	Cytoplasmic dynein 1 heavy chain 1	38955982.57	65	16	535.06	323.31
sp|Q7KZF4|SND1_HUMAN Staphylococcal nuclease domain-containing protein 1 OS=Homo sapiens OX=9606 GN=SND1 PE=1 SV=1	Q7KZF4	SND1	Staphylococcal nuclease domain-containing protein 1	34732013.79	48	53.2	102	323.31
sp|Q00610|CLH1_HUMAN Clathrin heavy chain 1 OS=Homo sapiens OX=9606	Q00610	CLTC	Clathrin heavy chain 1	6616201.729	73	48.2	191.61	323.31

GN=CLTC PE=1 SV=5;tr|A0A087WVQ6|A0A087WVQ6_HUMAN Clathrin heavy chain OS=Homo sapiens OX=9606 GN=CLTC PE=1 SV=1								
sp|Q9NZM1|MYOF_HUMAN Myoferlin OS=Homo sapiens OX=9606 GN=MYOF PE=1 SV=1;tr|H0YD14|H0YD14_HUMAN Myoferlin (Fragment) OS=Homo sapiens OX=9606 GN=MYOF PE=1 SV=1	Q9NZM1	MYOF	Myoferlin	5319697.11	50	30.6	234.71	323.31
sp|Q14152|EIF3A_HUMAN Eukaryotic translation initiation factor 3 subunit A OS=Homo sapiens OX=9606 GN=EIF3A PE=1 SV=1	Q14152	EIF3A	Eukaryotic translation initiation factor 3 subunit A	122340052.9	44	29.8	166.57	309.97
sp|Q9P2E9|RRBP1_HUMAN Ribosome-binding protein 1 OS=Homo sapiens OX=9606 GN=RRBP1 PE=1 SV=5;tr|A0A087WVV2|A0A087WVV2_HUMAN Ribosome-binding protein 1 OS=Homo sapiens OX=9606 GN=RRBP1 PE=1 SV=1;tr|F8W7S5|F8W7S5_HUMAN Ribosome-binding protein 1 OS=Homo sapie	Q9P2E9	RRBP1	Ribosome-binding protein 1	63284966.86	33	41.8	152.45	277.38
sp|P05023|AT1A1_HUMAN Sodium/potassium-transporting ATPase subunit alpha-1 OS=Homo sapiens OX=9606 GN=ATP1A1 PE=1 SV=1	P05023	ATP1A1	Sodium/potassium-transporting ATPase subunit alpha-1	133580088.1	27	29.8	112.89	272.18
sp|Q09666|AHNK_HUMAN Neuroblast differentiation-associated protein AHNAK OS=Homo sapiens OX=9606 GN=AHNAK PE=1 SV=2	Q09666	AHNAK	Neuroblast differentiation-associated protein AHNAK	105329990.7	41	18.9	629.09	263.03

sp|Q14008|CKAP5_HUMAN Cytoskeleton-associated protein 5 OS=Homo sapiens OX=9606 GN=CKAP5 PE=1 SV=3	Q14008	CKAP5	Cytoskeleton-associated protein 5	299980123.7	38	20.9	225.49	262.25
sp|P11498|PYC_HUMAN Pyruvate carboxylase, mitochondrial OS=Homo sapiens OX=9606 GN=PC PE=1 SV=2;tr|A0A494C016|A0A494C016_HUMAN Pyruvate carboxylase, mitochondrial OS=Homo sapiens OX=9606 GN=PC PE=1 SV=1	P11498	PC	Pyruvate carboxylase, mitochondrial	229470146.2	32	34.4	129.63	261.16
sp|P13647|K2C5_HUMAN Keratin, type II cytoskeletal 5 OS=Homo sapiens OX=9606 GN=KRT5 PE=1 SV=3	P13647	KRT5	Keratin, type II cytoskeletal 5	413079792.4	37	46.9	62.378	246.77
sp|Q9UDY2|ZO2_HUMAN Tight junction protein ZO-2 OS=Homo sapiens OX=9606 GN=TJP2 PE=1 SV=2;tr|A0A3B3ITE1|A0A3B3ITE1_HUMAN Tight junction protein ZO-2 (Fragment) OS=Homo sapiens OX=9606 GN=TJP2 PE=1 SV=1;tr|A0A1B0GTW1|A0A1B0GTW1_HUMAN Tight junction protein	Q9UDY2	TJP2	Tight junction protein ZO-2	620820281.5	31	29.8	133.96	241.76
tr|A0A7I2V428|A0A7I2V428_HUMAN Nucleolin OS=Homo sapiens OX=9606 GN=NCL PE=1 SV=1;tr|C9JLB1|C9JLB1_HUMAN Nucleolin OS=Homo sapiens OX=9606 GN=NCL PE=1 SV=2;sp|P19338|NUCL_HUMAN Nucleolin OS=Homo sapiens OX=9606 GN=NCL PE=1 SV=3;tr|H7BY16|H7BY16_HUMAN Nucle	A0A7I2V428	NCL	Nucleolin	86397984.62	31	40.2	74.739	237.88
sp|Q92900|RENT1_HUMAN Regulator of nonsense transcripts 1 OS=Homo sapiens OX=9606 GN=UPF1 PE=1 SV=2	Q92900	UPF1	Regulator of nonsense transcripts 1	176689931.7	30	33.7	124.34	221.69
sp|O95347|SMC2_HUMAN Structural maintenance of chromosomes protein 2	O95347	SMC2	Structural maintenance of	35078021.1	29	25.6	135.65	218.93

OS=Homo sapiens OX=9606 GN=SMC2 PE=1 SV=2			chromosomes protein 2					
sp|P62701|RS4X_HUMAN 40S ribosomal protein S4, X isoform OS=Homo sapiens OX=9606 GN=RPS4X PE=1 SV=2	P62701	RPS4X	40S ribosomal protein S4, X isoform	13562008.81	22	60.1	29.597	215.79
sp|P02533|K1C14_HUMAN Keratin, type I cytoskeletal 14 OS=Homo sapiens OX=9606 GN=KRT14 PE=1 SV=4	P02533	KRT14	Keratin, type I cytoskeletal 14	356960234.5	24	52.5	51.561	214.75
sp|P08670|VIME_HUMAN Vimentin OS=Homo sapiens OX=9606 GN=VIM PE=1 SV=4;tr|B0YJC4|B0YJC4_HUMAN Vimentin OS=Homo sapiens OX=9606 GN=VIM PE=1 SV=1	P08670	VIM	Vimentin	7799204.523	25	52.4	53.651	212.26
sp|Q9Y2L1|RRP44_HUMAN Exosome complex exonuclease RRP44 OS=Homo sapiens OX=9606 GN=DIS3 PE=1 SV=2;tr|G3V1J5|G3V1J5_HUMAN Exosome complex exonuclease RRP44 OS=Homo sapiens OX=9606 GN=DIS3 PE=1 SV=1	Q9Y2L1	DIS3	Exosome complex exonuclease RRP44	247730012.4	25	31.1	109	211.37
sp|P53396|ACLY_HUMAN ATP-citrate synthase OS=Homo sapiens OX=9606 GN=ACLY PE=1 SV=3	P53396	ACLY	ATP-citrate synthase	50012012.74	27	29.7	120.84	207.83
sp|P20930|FILA_HUMAN Filaggrin OS=Homo sapiens OX=9606 GN=FLG PE=1 SV=3	P20930	FLG	Filaggrin	98949969.23	29	18.1	435.16	203.96
sp|Q9HAV4|XPO5_HUMAN Exportin-5 OS=Homo sapiens OX=9606 GN=XPO5 PE=1 SV=1	Q9HAV4	XPO5	Exportin-5	1724999.417	14	14.5	136.31	199.33
tr|H3BQZ7|H3BQZ7_HUMAN HCG2044799 OS=Homo sapiens OX=9606 GN=HNRNPUL2-BSCL2 PE=4 SV=1;sp|Q1KMD3|HNRL2_HUMAN Heterogeneous nuclear ribonucleoprotein U-like protein 2 OS=Homo sapiens OX=9606 GN=HNRNPUL2 PE=1 SV=1	H3BQZ7	HNRNPUL2-BSCL2	HCG2044799	165629980.4	21	29.1	84.69	193.65

sp|P28340|DPOD1_HUMAN DNA polymerase delta catalytic subunit OS=Homo sapiens OX=9606 GN=POLD1 PE=1 SV=2;tr|M0R2B7|M0R2B7_HUMAN DNA polymerase OS=Homo sapiens OX=9606 GN=POLD1 PE=1 SV=1;tr|A0A087WYJ2|A0A087WYJ2_HUMAN DNA polymerase OS=Homo sapiens OX=9606 G	P28340	POLD1	DNA polymerase delta catalytic subunit	86183053.5	24	26.6	123.63	192.54
sp|Q92974|ARHG2_HUMAN Rho guanine nucleotide exchange factor 2 OS=Homo sapiens OX=9606 GN=ARHGEF2 PE=1 SV=4;tr|V9GYM8|V9GYM8_HUMAN Guanine nucleotide exchange factor H1 OS=Homo sapiens OX=9606 GN=ARHGEF2 PE=1 SV=1;tr|A0A5F9ZI21|A0A5F9ZI21_HUMAN Guanine nuc	Q92974	ARHGEF2	Rho guanine nucleotide exchange factor 2	184350027.5	25	31.7	111.54	189.54
sp|Q9Y3F4|STRAP_HUMAN Serine-threonine kinase receptor-associated protein OS=Homo sapiens OX=9606 GN=STRAP PE=1 SV=1	Q9Y3F4	STRAP	Serine-threonine kinase receptor-associated protein	84098947.63	15	57.1	38.438	186.67
sp|P23396|RS3_HUMAN 40S ribosomal protein S3 OS=Homo sapiens OX=9606 GN=RPS3 PE=1 SV=2;tr|E9PL09|E9PL09_HUMAN 40S ribosomal protein S3 OS=Homo sapiens OX=9606 GN=RPS3 PE=1 SV=1;tr|E9PPU1|E9PPU1_HUMAN 40S ribosomal protein S3 OS=Homo sapiens OX=9606 GN=RPS3	P23396	RPS3	40S ribosomal protein S3	20828995.36	22	74.5	26.688	183.78
sp|O60231|DHX16_HUMAN Pre-mRNA-splicing factor ATP-dependent RNA helicase DHX16 OS=Homo sapiens OX=9606	O60231	DHX16	Pre-mRNA-splicing factor ATP-	131079952.2	24	26.1	119.26	182.81

GN=DHX16 PE=1 SV=2;tr|A0A140T947|A0A140T947_HUMAN Pre-mRNA-splicing factor ATP-dependent RNA helicase DHX16 (Fragment) OS=Homo sapiens OX=9606 GN=DHX16			dependent RNA helicase DHX16					
tr|A0A804HJA4|A0A804HJA4_HUMAN Proteasome adapter and scaffold protein ECM29 OS=Homo sapiens OX=9606 GN=ECPAS PE=4 SV=1;sp|Q5VYK3|ECM29_HUMAN Proteasome adapter and scaffold protein ECM29 OS=Homo sapiens OX=9606 GN=ECPAS PE=1 SV=2;tr|J3KN16|J3KN16_HUMAN Pr	A0A804HJA4	ECPAS	Proteasome adapter and scaffold protein ECM29	1203399.884	24	16.2	203.99	179.56
sp|Q86VI3|IQGA3_HUMAN Ras GTPase-activating-like protein IQGAP3 OS=Homo sapiens OX=9606 GN=IQGAP3 PE=1 SV=2;tr|F2Z2E2|F2Z2E2_HUMAN Ras GTPase-activating-like protein IQGAP3 OS=Homo sapiens OX=9606 GN=IQGAP3 PE=1 SV=1	Q86VI3	IQGAP3	Ras GTPase-activating-like protein IQGAP3	64808013.81	26	17.9	184.7	175.72
sp|P17301|ITA2_HUMAN Integrin alpha-2 OS=Homo sapiens OX=9606 GN=ITGA2 PE=1 SV=1	P17301	ITGA2	Integrin alpha-2	66180997.99	24	24.6	129.29	172.27
sp|Q9P0K7|RAI14_HUMAN Ankycorbin OS=Homo sapiens OX=9606 GN=RAI14 PE=1 SV=2	Q9P0K7	RAI14	Ankycorbin	82923015.27	20	23.5	110.04	170.43
tr|H7BXY3|H7BXY3_HUMAN RNA helicase OS=Homo sapiens OX=9606 GN=DHX30 PE=1 SV=1;sp|Q7L2E3|DHX30_HUMAN ATP-dependent RNA helicase DHX30 OS=Homo sapiens OX=9606 GN=DHX30 PE=1 SV=1	H7BXY3	DHX30	RNA helicase	42011021.4	22	21.4	130.55	168.35
sp|Q15029|U5S1_HUMAN 116 kDa U5 small nuclear ribonucleoprotein component OS=Homo sapiens OX=9606 GN=EFTUD2 PE=1 SV=1	Q15029	EFTUD2	116 kDa U5 small nuclear ribonucleoprotein component	64599032.93	21	29.2	109.43	168.07

sp|P06748|NPM_HUMAN Nucleophosmin OS=Homo sapiens OX=9606 GN=NPM1 PE=1 SV=2;tr|A0A7I2V5S2|A0A7I2V5S2_HUMAN Nucleophosmin OS=Homo sapiens OX=9606 GN=NPM1 PE=1 SV=1;tr|A0A7I2YQC0|A0A7I2YQC0_HUMAN Nucleophosmin OS=Homo sapiens OX=9606 GN=NPM1 PE=1 SV=1	P06748	NPM1	Nucleophosmin	5674896842	19	51.4	32.575	160.81
sp|Q13045|FLII_HUMAN Protein flightless-1 homolog OS=Homo sapiens OX=9606 GN=FLII PE=1 SV=2	Q13045	FLII	Protein flightless-1 homolog	33324984.32	20	18.4	144.75	150.7
sp|P27816|MAP4_HUMAN Microtubule-associated protein 4 OS=Homo sapiens OX=9606 GN=MAP4 PE=1 SV=3;tr|A0A804HKE7|A0A804HKE7_HUMAN Microtubule-associated protein 4 OS=Homo sapiens OX=9606 GN=MAP4 PE=4 SV=1;tr|E7EVA0|E7EVA0_HUMAN Microtubule-associated protein	P27816	MAP4	Microtubule-associated protein 4	142949938	19	20.8	121	145.56
tr|E9PD53|E9PD53_HUMAN Structural maintenance of chromosomes protein OS=Homo sapiens OX=9606 GN=SMC4 PE=1 SV=1;sp|Q9NTJ3|SMC4_HUMAN Structural maintenance of chromosomes protein 4 OS=Homo sapiens OX=9606 GN=SMC4 PE=1 SV=2	E9PD53	SMC4	Structural maintenance of chromosomes protein	26066000.37	20	17.8	144.45	143.43
sp|P05198|IF2A_HUMAN Eukaryotic translation initiation factor 2 subunit 1 OS=Homo sapiens OX=9606 GN=EIF2S1 PE=1 SV=3;tr|G3V4T5|G3V4T5_HUMAN Eukaryotic translation initiation factor 2 subunit 1 (Fragment) OS=Homo sapiens OX=9606 GN=EIF2S1 PE=1 SV=1;tr|H0YJ	P05198	EIF2S1	Eukaryotic translation initiation factor 2 subunit 1	268299921.7	16	53.7	36.112	141.54

tr|A0A7I2Y1C3|A0A7I2Y1C3_HUMAN Eukaryotic translation initiation factor 4 gamma 1 OS=Homo sapiens OX=9606 GN=EIF4G1 PE=1 SV=1;sp|Q04637|IF4G1_HUMAN Eukaryotic translation initiation factor 4 gamma 1 OS=Homo sapiens OX=9606 GN=EIF4G1 PE=1 SV=4;tr|E7EX73|E7E	A0A7I2Y1C3	EIF4G1	Eukaryotic translation initiation factor 4 gamma 1	191739981.6	20	15.3	173.11	139.85
sp|Q07157|ZO1_HUMAN Tight junction protein ZO-1 OS=Homo sapiens OX=9606 GN=TJP1 PE=1 SV=3;tr|A0A087X0K9|A0A087X0K9_HUMAN Tight junction protein 1 (Zona occludens 1), isoform CRA_a OS=Homo sapiens OX=9606 GN=TJP1 PE=1 SV=2;tr|G3V1L9|G3V1L9_HUMAN Tight junct	Q07157	TJP1	Tight junction protein ZO-1	127500034.3	21	16	195.46	137.46
tr|A0A804HJG7|A0A804HJG7_HUMAN CLIP-associating protein 2 OS=Homo sapiens OX=9606 GN=CLASP2 PE=4 SV=1;tr|E3W994|E3W994_HUMAN CLIP-associating protein 2 OS=Homo sapiens OX=9606 GN=CLASP2 PE=1 SV=2;tr|E7ERI8|E7ERI8_HUMAN CLIP-associating protein 2 OS=Homo sa	A0A804HJG7	CLASP2	CLIP-associating protein 2	89894057.45	16	14.1	164.88	137.33
sp|Q86YZ3|HORN_HUMAN Hornerin OS=Homo sapiens OX=9606 GN=HRNR PE=1 SV=2	Q86YZ3	HRNR	Hornerin	83447052.58	18	19.1	282.39	137.12
sp|Q14444|CAPR1_HUMAN Caprin-1 OS=Homo sapiens OX=9606 GN=CAPRIN1 PE=1 SV=2;tr|G3V153|G3V153_HUMAN Caprin-1 OS=Homo sapiens OX=9606 GN=CAPRIN1 PE=1 SV=1;tr|E9PLA9|E9PLA9_HUMAN Caprin-1 (Fragment) OS=Homo sapiens OX=9606 GN=CAPRIN1 PE=1 SV=1	Q14444	CAPRIN1	Caprin-1	29027987.91	17	23.7	78.365	135.72

tr|H7C4K3|H7C4K3_HUMAN Integrin beta OS=Homo sapiens OX=9606 GN=ITGB1 PE=1 SV=2;tr|A0A7I2V2F9|A0A7I2V2F9_HUMAN Integrin beta OS=Homo sapiens OX=9606 GN=ITGB1 PE=1 SV=1;sp|P05556|ITB1_HUMAN Integrin beta-1 OS=Homo sapiens OX=9606 GN=ITGB1 PE=1 SV=2;tr|E7EUI	H7C4K3	ITGB1	Integrin beta	50683025.54	18	24.3	82.23	135.35
sp|P20020|AT2B1_HUMAN Plasma membrane calcium-transporting ATPase 1 OS=Homo sapiens OX=9606 GN=ATP2B1 PE=1 SV=4;tr|E7ERY9|E7ERY9_HUMAN Calcium-transporting ATPase OS=Homo sapiens OX=9606 GN=ATP2B1 PE=1 SV=2	P20020	ATP2B1	Plasma membrane calcium-transporting ATPase 1	35096993.9	10	10.8	134.68	132.99
sp|P78527|PRKDC_HUMAN DNA-dependent protein kinase catalytic subunit OS=Homo sapiens OX=9606 GN=PRKDC PE=1 SV=3	P78527	PRKDC	DNA-dependent protein kinase catalytic subunit	30307997.34	19	5.5	469.08	131.27
sp|Q15021|CND1_HUMAN Condensin complex subunit 1 OS=Homo sapiens OX=9606 GN=NCAPD2 PE=1 SV=3	Q15021	NCAPD2	Condensin complex subunit 1	29383990.37	17	15.3	157.18	131.16
sp|O00203|AP3B1_HUMAN AP-3 complex subunit beta-1 OS=Homo sapiens OX=9606 GN=AP3B1 PE=1 SV=3	O00203	AP3B1	AP-3 complex subunit beta-1	3194499.894	19	21	121.32	129.57
sp|P04406|G3P_HUMAN Glyceraldehyde-3-phosphate dehydrogenase OS=Homo sapiens OX=9606 GN=GAPDH PE=1 SV=3;tr|E7EUT5|E7EUT5_HUMAN Glyceraldehyde-3-phosphate dehydrogenase OS=Homo sapiens OX=9606 GN=GAPDH PE=1 SV=1	P04406	GAPDH	Glyceraldehyde-3-phosphate dehydrogenase	43305981.57	15	54.9	36.053	129.13
tr|H0YNH8|H0YNH8_HUMAN Uveal autoantigen with coiled-coil domains and	H0YNH8	UACA	Uveal autoantigen	77509023.8	15	12.8	161.31	127.62

ankyrin repeats OS=Homo sapiens OX=9606 GN=UACA PE=1 SV=1;sp|Q9BZF9|UACA_HUMAN Uveal autoantigen with coiled-coil domains and ankyrin repeats OS=Homo sapiens OX=9606 GN=UACA PE=1 SV=2;tr			with coiled-coil domains and ankyrin repeats					
sp|Q8N163|CCAR2_HUMAN Cell cycle and apoptosis regulator protein 2 OS=Homo sapiens OX=9606 GN=CCAR2 PE=1 SV=2;tr|H0YB24|H0YB24_HUMAN Cell cycle and apoptosis regulator protein 2 (Fragment) OS=Homo sapiens OX=9606 GN=CCAR2 PE=1 SV=1;tr|G3V119|G3V119_HUMAN C	Q8N163	CCAR2	Cell cycle and apoptosis regulator protein 2	33890993.06	17	22.9	102.9	122.42
tr|E7EPN9|E7EPN9_HUMAN Protein PRRC2C OS=Homo sapiens OX=9606 GN=PRRC2C PE=1 SV=1;sp|Q9Y520|PRC2C_HUMAN Protein PRRC2C OS=Homo sapiens OX=9606 GN=PRRC2C PE=1 SV=4	E7EPN9	PRRC2C	Protein PRRC2C	67027022.76	18	6.6	308.77	122.4
sp|Q9Y613|FHOD1_HUMAN FH1/FH2 domain-containing protein 1 OS=Homo sapiens OX=9606 GN=FHOD1 PE=1 SV=3	Q9Y613	FHOD1	FH1/FH2 domain-containing protein 1	55348033.83	14	17.3	126.55	120.46
sp|O75643|U520_HUMAN U5 small nuclear ribonucleoprotein 200 kDa helicase OS=Homo sapiens OX=9606 GN=SNRNP200 PE=1 SV=2;tr|A0A494C1A5|A0A494C1A5_HUMAN U5 small nuclear ribonucleoprotein 200 kDa helicase (Fragment) OS=Homo sapiens OX=9606 GN=SNRNP200 PE=1 SV	O75643	SNRNP200	U5 small nuclear ribonucleoprotein 200 kDa helicase	83940991.12	17	9.2	244.5	119.9
tr|H0YMW4|H0YMW4_HUMAN Annexin OS=Homo sapiens OX=9606 GN=ANXA2 PE=1 SV=2;sp|P07355|ANXA2_HUMAN Annexin A2 OS=Homo sapiens OX=9606 GN=ANXA2 PE=1	H0YMW4	ANXA2	Annexin	10571993.07	7	25.6	41.899	119.02

SV=2;tr|H0YN42|H0YN42_HUMAN Annexin (Fragment) OS=Homo sapiens OX=9606 GN=ANXA2 PE=1 SV=1;tr|H0YMU9|H0YMU9_HUMA								
sp|P26006|ITA3_HUMAN Integrin alpha-3 OS=Homo sapiens OX=9606 GN=ITGA3 PE=1 SV=5	P26006	ITGA3	Integrin alpha-3	76816043.77	13	12.7	116.61	118.08
sp|P55884|EIF3B_HUMAN Eukaryotic translation initiation factor 3 subunit B OS=Homo sapiens OX=9606 GN=EIF3B PE=1 SV=3	P55884	EIF3B	Eukaryotic translation initiation factor 3 subunit B	60369030.56	17	21.7	92.48	117.7
tr|E9PDF6|E9PDF6_HUMAN Unconventional myosin-Ib OS=Homo sapiens OX=9606 GN=MYO1B PE=1 SV=1;sp|O43795|MYO1B_HUMAN Unconventional myosin-Ib OS=Homo sapiens OX=9606 GN=MYO1B PE=1 SV=3	E9PDF6	MYO1B	Unconventional myosin-Ib	4094001.673	16	16.6	128.48	114.52
sp|Q14847|LASP1_HUMAN LIM and SH3 domain protein 1 OS=Homo sapiens OX=9606 GN=LASP1 PE=1 SV=2;tr|C9J9W2|C9J9W2_HUMAN LIM and SH3 domain protein 1 (Fragment) OS=Homo sapiens OX=9606 GN=LASP1 PE=1 SV=1	Q14847	LASP1	LIM and SH3 domain protein 1	27936005.38	13	41.4	29.717	112.14
sp|Q6Y7W6|GGYF2_HUMAN GRB10-interacting GYF protein 2 OS=Homo sapiens OX=9606 GN=GIGYF2 PE=1 SV=1;tr|A0A7I2V3H7|A0A7I2V3H7_HUMAN GRB10-interacting GYF protein 2 OS=Homo sapiens OX=9606 GN=GIGYF2 PE=1 SV=1;tr|A0A7I2V361|A0A7I2V361_HUMAN GRB10-interacting GY	Q6Y7W6	GIGYF2	GRB10-interacting GYF protein 2	36057022.5	11	9.5	150.07	103.52

tr|F8W726|F8W726_HUMAN Ubiquitin-associated protein 2-like OS=Homo sapiens OX=9606 GN=UBAP2L PE=1 SV=2;sp|Q14157|UBP2L_HUMAN Ubiquitin-associated protein 2-like OS=Homo sapiens OX=9606 GN=UBAP2L PE=1 SV=2	F8W726	UBAP2L	Ubiquitin-associated protein 2-like	58073017.76	13	17.2	113.63	102.93
sp|P62753|RS6_HUMAN 40S ribosomal protein S6 OS=Homo sapiens OX=9606 GN=RPS6 PE=1 SV=1;tr|A2A3R5|A2A3R5_HUMAN 40S ribosomal protein S6 OS=Homo sapiens OX=9606 GN=RPS6 PE=1 SV=1	P62753	RPS6	40S ribosomal protein S6	31157001.35	13	45	28.68	102.59
sp|P52701|MSH6_HUMAN DNA mismatch repair protein Msh6 OS=Homo sapiens OX=9606 GN=MSH6 PE=1 SV=2;tr|A0A494C0M1|A0A494C0M1_HUMAN DNA mismatch repair protein OS=Homo sapiens OX=9606 GN=MSH6 PE=1 SV=1;tr|A0A087WWJ1|A0A087WWJ1_HUMAN DNA mismatch repair protein	P52701	MSH6	DNA mismatch repair protein Msh6	7763896.92	14	12	152.78	100.84
sp|P62136|PP1A_HUMAN Serine/threonine-protein phosphatase PP1-alpha catalytic subunit OS=Homo sapiens OX=9606 GN=PPP1CA PE=1 SV=1;tr|E9PMD7|E9PMD7_HUMAN Serine/threonine-protein phosphatase OS=Homo sapiens OX=9606 GN=PPP1CA PE=1 SV=2;tr|A0A7I2V4F7|A0A7I2V4	P62136	PPP1CA	Serine/threonine-protein phosphatase PP1-alpha catalytic subunit	90537047.79	13	47.3	37.512	99.699
sp|P02538|K2C6A_HUMAN Keratin, type II cytoskeletal 6A OS=Homo sapiens OX=9606 GN=KRT6A PE=1 SV=3	P02538	KRT6A	Keratin, type II cytoskeletal 6A	85943997.06	29	42	60.044	99.048
sp|Q12797|ASPH_HUMAN Aspartyl/asparaginyl beta-hydroxylase OS=Homo sapiens OX=9606 GN=ASPH PE=1 SV=3	Q12797	ASPH	Aspartyl/asparaginyl beta-hydroxylase	43120990.39	12	19.8	85.862	98.718

sp|Q9HAU0|PKHA5_HUMAN Pleckstrin homology domain-containing family A member 5 OS=Homo sapiens OX=9606 GN=PLEKHA5 PE=1 SV=1;tr|A0JP02|A0JP02_HUMAN PLEKHA5 protein OS=Homo sapiens OX=9606 GN=PLEKHA5 PE=1 SV=1;tr|B4DJX4|B4DJX4_HUMAN Pleckstrin homology domain	Q9HAU0	PLEKHA5	Pleckstrin homology domain-containing family A member 5	45852972.69	9	11.8	127.46	96.881
sp|O14974|MYPT1_HUMAN Protein phosphatase 1 regulatory subunit 12A OS=Homo sapiens OX=9606 GN=PPP1R12A PE=1 SV=1;tr|F8VZN8|F8VZN8_HUMAN Protein phosphatase 1 regulatory subunit 12A (Fragment) OS=Homo sapiens OX=9606 GN=PPP1R12A PE=1 SV=1	O14974	PPP1R12A	Protein phosphatase 1 regulatory subunit 12A	125530060.2	14	16.4	115.28	96.37
sp|Q6PKG0|LARP1_HUMAN La-related protein 1 OS=Homo sapiens OX=9606 GN=LARP1 PE=1 SV=2;tr|A0A0B4J210|A0A0B4J210_HUMAN La-related protein 1 (Fragment) OS=Homo sapiens OX=9606 GN=LARP1 PE=1 SV=5	Q6PKG0	LARP1	La-related protein 1	97144978.85	13	14.1	123.51	96.287
sp|P08754|GNAI3_HUMAN Guanine nucleotide-binding protein G(i) subunit alpha-3 OS=Homo sapiens OX=9606 GN=GNAI3 PE=1 SV=3	P08754	GNAI3	Guanine nucleotide-binding protein G(i) subunit alpha-3	15634002.34	13	42.9	40.532	92.415
sp|Q6P1N0|C2D1A_HUMAN Coiled-coil and C2 domain-containing protein 1A OS=Homo sapiens OX=9606 GN=CC2D1A PE=1 SV=1;tr|A0A7P0Z4M5|A0A7P0Z4M5_HUMAN Coiled-coil and C2 domain-containing protein 1A (Fragment) OS=Homo sapiens OX=9606 GN=CC2D1A PE=1 SV=1;tr|K7EMP	Q6P1N0	CC2D1A	Coiled-coil and C2 domain-containing protein 1A	50289024.56	14	18.9	104.06	90.727

sp|O75533|SF3B1_HUMAN Splicing factor 3B subunit 1 OS=Homo sapiens OX=9606 GN=SF3B1 PE=1 SV=3	O75533	SF3B1	Splicing factor 3B subunit 1	25688006.48	13	12.2	145.83	89.957
sp|O60343|TBCD4_HUMAN TBC1 domain family member 4 OS=Homo sapiens OX=9606 GN=TBC1D4 PE=1 SV=2;tr|A0A3B3IRT3|A0A3B3IRT3_HUMAN TBC1 domain family member 4 OS=Homo sapiens OX=9606 GN=TBC1D4 PE=1 SV=1	O60343	TBC1D4	TBC1 domain family member 4	41770992.21	11	9	146.56	89.839
sp|P61247|RS3A_HUMAN 40S ribosomal protein S3a OS=Homo sapiens OX=9606 GN=RPS3A PE=1 SV=2;tr|D6RG13|D6RG13_HUMAN 40S ribosomal protein S3a (Fragment) OS=Homo sapiens OX=9606 GN=RPS3A PE=1 SV=1;tr|D6RB09|D6RB09_HUMAN 40S ribosomal protein S3a (Fragment) OS=	P61247	RPS3A	40S ribosomal protein S3a	7131196.553	13	46.2	29.945	87.519
sp|P08779|K1C16_HUMAN Keratin, type I cytoskeletal 16 OS=Homo sapiens OX=9606 GN=KRT16 PE=1 SV=4	P08779	KRT16	Keratin, type I cytoskeletal 16	57866013.48	21	46.3	51.267	87.408
sp|Q70E73|RAPH1_HUMAN Ras-associated and pleckstrin homology domains-containing protein 1 OS=Homo sapiens OX=9606 GN=RAPH1 PE=1 SV=3;tr|C9K0J5|C9K0J5_HUMAN Ras association (RalGDS/AF-6) and pleckstrin homology domains 1, isoform CRA_b OS=Homo sapiens OX=96	Q70E73	RAPH1	Ras-associated and pleckstrin homology domains-containing protein 1	43668012.9	13	12	135.25	87.297
sp|O15056|SYNJ2_HUMAN Synaptojanin-2 OS=Homo sapiens OX=9606 GN=SYNJ2 PE=1 SV=3;tr|A0A1W2PR85|A0A1W2PR85_HUMAN Phosphoinositide 5-phosphatase OS=Homo sapiens OX=9606 GN=SYNJ2 PE=1 SV=1;tr|E7ER60|E7ER60_HUMAN	O15056	SYNJ2	Synaptojanin-2	34703005.56	10	8.2	165.54	86.785

Synaptojanin-2 OS=Homo sapiens OX=9606 GN=SYNJ2								
tr|F5H658|F5H658_HUMAN RNA helicase OS=Homo sapiens OX=9606 GN=DHX8 PE=1 SV=1;sp|Q14562|DHX8_HUMAN ATP-dependent RNA helicase DHX8 OS=Homo sapiens OX=9606 GN=DHX8 PE=1 SV=1	F5H658	DHX8	RNA helicase	75740043.68	12	13.2	134	84.922
tr|A0A0C4DGA2|A0A0C4DGA2_HUMAN Enoyl-CoA delta isomerase 2, mitochondrial OS=Homo sapiens OX=9606 GN=ECI2 PE=1 SV=1;sp|O75521|ECI2_HUMAN Enoyl-CoA delta isomerase 2 OS=Homo sapiens OX=9606 GN=ECI2 PE=1 SV=4	A0A0C4DGA2	ECI2	Enoyl-CoA delta isomerase 2, mitochondrial	101229984.9	11	33.8	40.183	84.214
tr|A0A7I2YQF8|A0A7I2YQF8_HUMAN Eukaryotic translation initiation factor 3 subunit I OS=Homo sapiens OX=9606 GN=EIF3I PE=1 SV=1;sp|Q13347|EIF3I_HUMAN Eukaryotic translation initiation factor 3 subunit I OS=Homo sapiens OX=9606 GN=EIF3I PE=1 SV=1;tr|A0A7I2YQ	A0A7I2YQF8	EIF3I	Eukaryotic translation initiation factor 3 subunit I	146489947.4	12	38.1	40.489	83.666
sp|P29317|EPHA2_HUMAN Ephrin type-A receptor 2 OS=Homo sapiens OX=9606 GN=EPHA2 PE=1 SV=2	P29317	EPHA2	Ephrin type-A receptor 2	24255985.55	11	13.2	108.27	83.278
tr|A0A0A0MRA5|A0A0A0MRA5_HUMAN Heterogeneous nuclear ribonucleoprotein U-like protein 1 OS=Homo sapiens OX=9606 GN=HNRNPUL1 PE=1 SV=1;tr|B7Z4B8|B7Z4B8_HUMAN Heterogeneous nuclear ribonucleoprotein U-like protein 1 OS=Homo sapiens OX=9606 GN=HNRNPUL1 PE=1 S	A0A0A0MRA5	HNRNPUL1	Heterogeneous nuclear ribonucleoprotein U-like protein 1	14677997.23	12	20.2	85.939	82.903
sp|Q15024|EXOS7_HUMAN Exosome complex component RRP42 OS=Homo sapiens OX=9606 GN=EXOSC7 PE=1 SV=3	Q15024	EXOSC7	Exosome complex	99957002.72	5	25.4	31.821	82.223

			component RRP42					
sp|Q9NZB2|F120A_HUMAN Constitutive coactivator of PPAR-gamma-like protein 1 OS=Homo sapiens OX=9606 GN=FAM120A PE=1 SV=2;tr|A0A0C4DG79|A0A0C4DG79_HUMAN Constitutive coactivator of PPAR-gamma-like protein 1 (Fragment) OS=Homo sapiens OX=9606 GN=FAM120A PE=1	Q9NZB2	FAM120A	Constitutive coactivator of PPAR-gamma-like protein 1	58676992.04	10	11.6	121.89	81.398
sp|Q9BXP5|SRRT_HUMAN Serrate RNA effector molecule homolog OS=Homo sapiens OX=9606 GN=SRRT PE=1 SV=1;tr|H7C3A1|H7C3A1_HUMAN Arsenite-resistance protein 2 (Fragment) OS=Homo sapiens OX=9606 GN=SRRT PE=1 SV=1	Q9BXP5	SRRT	Serrate RNA effector molecule homolog	22381004.99	11	12.7	100.67	78.197
sp|Q6WCQ1|MPRIP_HUMAN Myosin phosphatase Rho-interacting protein OS=Homo sapiens OX=9606 GN=MPRIP PE=1 SV=3;tr|A0A494BZV2|A0A494BZV2_HUMAN Myosin phosphatase Rho-interacting protein OS=Homo sapiens OX=9606 GN=MPRIP PE=1 SV=1;tr|J3KSW8|J3KSW8_HUMAN Myosin p	Q6WCQ1	MPRIP	Myosin phosphatase Rho-interacting protein	50614992.46	9	10.5	116.53	77.61
tr|A0A0C4DGB6|A0A0C4DGB6_HUMAN Albumin OS=Homo sapiens OX=9606 GN=ALB PE=1 SV=1;sp|P02768|ALBU_HUMAN Albumin OS=Homo sapiens OX=9606 GN=ALB PE=1 SV=2;tr|B7WNR0|B7WNR0_HUMAN Albumin OS=Homo sapiens OX=9606 GN=ALB PE=1 SV=1;tr|C9JKR2|C9JKR2_HUMAN Albumin OS=	A0A0C4DGB6	ALB	Albumin	100630054.4	11	17.7	69.226	77.189

sp|O75717|WDHD1_HUMAN WD repeat and HMG-box DNA-binding protein 1 OS=Homo sapiens OX=9606 GN=WDHD1 PE=1 SV=1	O75717	WDHD 1	WD repeat and HMG-box DNA-binding protein 1	26492987. 59	10	10.8	125.97	76.515
sp|Q9BPX3|CND3_HUMAN Condensin complex subunit 3 OS=Homo sapiens OX=9606 GN=NCAPG PE=1 SV=1	Q9BPX3	NCAP G	Condensin complex subunit 3	19423012. 67	9	10.9	114.33	75.488
sp|O75477|ERLN1_HUMAN Erlin-1 OS=Homo sapiens OX=9606 GN=ERLIN1 PE=1 SV=2;tr|B0QZ43|B0QZ43_HUMAN Erlin-1 (Fragment) OS=Homo sapiens OX=9606 GN=ERLIN1 PE=1 SV=1	O75477	ERLIN 1	Erlin-1	46095977. 07	10	35.3	39.171	74.342
sp|Q13155|AIMP2_HUMAN Aminoacyl tRNA synthase complex-interacting multifunctional protein 2 OS=Homo sapiens OX=9606 GN=AIMP2 PE=1 SV=2;tr|A8MU58|A8MU58_HUMAN Aminoacyl tRNA synthase complex-interacting multifunctional protein 2 OS=Homo sapiens OX=9606 GN=A	Q13155	AIMP2	Aminoacyl tRNA synthase complex-interacting multifunctional protein 2	70386998 1.6	11	40	35.348	74.211
sp|Q13136|LIPA1_HUMAN Liprin-alpha-1 OS=Homo sapiens OX=9606 GN=PPFIA1 PE=1 SV=1;tr|A0A2R8Y7R9|A0A2R8Y7R9_HUMA N Liprin-alpha-1 (Fragment) OS=Homo sapiens OX=9606 GN=PPFIA1 PE=1 SV=1;tr|H0YDW2|H0YDW2_HUMAN Liprin-alpha-1 (Fragment) OS=Homo sapiens OX=9606 G	Q13136	PPFIA1	Liprin-alpha-1	12423007. 59	3	4.1	135.78	70.796
sp|Q9HAU5|RENT2_HUMAN Regulator of nonsense transcripts 2 OS=Homo sapiens OX=9606 GN=UPF2 PE=1 SV=1	Q9HAU 5	UPF2	Regulator of nonsense transcripts 2	29918997. 24	10	8.6	147.81	70.03
sp|O95466|FMNL1_HUMAN Formin-like protein 1 OS=Homo sapiens OX=9606	O95466	FMNL 1	Formin-like protein 1	14317994. 2	10	10.3	121.85	69.628

GN=FMNL1 PE=1 SV=3;tr|K7EJE6|K7EJE6_HUMAN Formin-like protein 1 (Fragment) OS=Homo sapiens OX=9606 GN=FMNL1 PE=1 SV=1;tr|K7EK60|K7EK60_HUMAN Formin-like protein 1 OS=Homo sapiens OX=9606 G								
sp|Q15365|PCBP1_HUMAN Poly(rC)-binding protein 1 OS=Homo sapiens OX=9606 GN=PCBP1 PE=1 SV=2	Q15365	PCBP1	Poly(rC)-binding protein 1	16555008.19	8	29.8	37.497	69.365
sp|Q8WWM7|ATX2L_HUMAN Ataxin-2-like protein OS=Homo sapiens OX=9606 GN=ATXN2L PE=1 SV=2;tr|H3BUF6|H3BUF6_HUMAN Ataxin-2-like protein OS=Homo sapiens OX=9606 GN=ATXN2L PE=1 SV=1	Q8WWM7	ATXN2L	Ataxin-2-like protein	9339197.602	11	12.2	113.37	69.217
tr|A0A7I2V3H3|A0A7I2V3H3_HUMAN Elongation factor 1-alpha OS=Homo sapiens OX=9606 GN=EEF1A1 PE=1 SV=1;tr|A0A7I2V5N4|A0A7I2V5N4_HUMAN Elongation factor 1-alpha OS=Homo sapiens OX=9606 GN=EEF1A1 PE=1 SV=1;tr|A0A7I2V659|A0A7I2V659_HUMAN Elongation factor 1-alp	A0A7I2V3H3	EEF1A1	Elongation factor 1-alpha	156939953.4	7	25.5	40.853	69.129
sp|Q9H0D6|XRN2_HUMAN 5-3 exoribonuclease 2 OS=Homo sapiens OX=9606 GN=XRN2 PE=1 SV=1	Q9H0D6	XRN2	5-3 exoribonuclease 2	21216005.96	11	15.7	108.58	68.548
sp|Q14694|UBP10_HUMAN Ubiquitin carboxyl-terminal hydrolase 10 OS=Homo sapiens OX=9606 GN=USP10 PE=1 SV=2	Q14694	USP10	Ubiquitin carboxyl-terminal hydrolase 10	17634991.7	10	17.2	87.133	67.744
sp|Q6P2Q9|PRP8_HUMAN Pre-mRNA-processing-splicing factor 8 OS=Homo sapiens OX=9606 GN=PRPF8 PE=1 SV=2	Q6P2Q9	PRPF8	Pre-mRNA-processing-splicing factor 8	34719985.28	10	4.5	273.6	64.401

sp|Q70UQ0|IKIP_HUMAN Inhibitor of nuclear factor kappa-B kinase-interacting protein OS=Homo sapiens OX=9606 GN=IKBIP PE=1 SV=1	Q70UQ0	IKBIP	Inhibitor of nuclear factor kappa-B kinase-interacting protein	48186995.1	6	16.6	39.309	63.04
sp|Q96QR8|PURB_HUMAN Transcriptional activator protein Pur-beta OS=Homo sapiens OX=9606 GN=PURB PE=1 SV=3	Q96QR8	PURB	Transcriptional activator protein Pur-beta	63834017.49	8	24.4	33.24	62.512
sp|Q86W92|LIPB1_HUMAN Liprin-beta-1 OS=Homo sapiens OX=9606 GN=PPFIBP1 PE=1 SV=2;tr|F5GZP6|F5GZP6_HUMAN Liprin-beta-1 (Fragment) OS=Homo sapiens OX=9606 GN=PPFIBP1 PE=1 SV=1;tr|H0YFE4|H0YFE4_HUMAN Liprin-beta-1 (Fragment) OS=Homo sapiens OX=9606 GN=PPFIBP1	Q86W92	PPFIBP1	Liprin-beta-1	31820987.36	9	12.4	114.02	61.857
sp|Q9BZH6|WDR11_HUMAN WD repeat-containing protein 11 OS=Homo sapiens OX=9606 GN=WDR11 PE=1 SV=1	Q9BZH6	WDR11	WD repeat-containing protein 11	18239990.65	7	8.6	136.68	61.499
sp|Q66K74|MAP1S_HUMAN Microtubule-associated protein 1S OS=Homo sapiens OX=9606 GN=MAP1S PE=1 SV=2	Q66K74	MAP1S	Microtubule-associated protein 1S	26469005.86	10	11.9	112.21	61.352
sp|Q9NSV4|DIAP3_HUMAN Protein diaphanous homolog 3 OS=Homo sapiens OX=9606 GN=DIAPH3 PE=1 SV=4	Q9NSV4	DIAPH3	Protein diaphanous homolog 3	15423999.28	5	4.8	136.92	61.352
tr|A0A384DVU0|A0A384DVU0_HUMAN Patatin-like phospholipase domain-containing protein 6 OS=Homo sapiens OX=9606 GN=PNPLA6 PE=1 SV=1;sp|Q8IY17|PLPL6_HUMAN Patatin-like phospholipase domain-containing protein 6	A0A384DVU0	PNPLA6	Patatin-like phospholipase domain-containing protein 6	9694598.844	6	5.9	149.92	60.976

OS=Homo sapiens OX=9606 GN=PNPLA6 PE=1 SV=3								
sp|Q16706|MA2A1_HUMAN Alpha-mannosidase 2 OS=Homo sapiens OX=9606 GN=MAN2A1 PE=1 SV=2	Q16706	MAN2A1	Alpha-mannosidase 2	13365004.76	9	9.1	131.14	60.955
sp|P42285|MTREX_HUMAN Exosome RNA helicase MTR4 OS=Homo sapiens OX=9606 GN=MTREX PE=1 SV=3	P42285	MTREX	Exosome RNA helicase MTR4	15894998.56	8	8.6	117.8	60.652
tr|F8VWS0|F8VWS0_HUMAN 60S acidic ribosomal protein P0 OS=Homo sapiens OX=9606 GN=RPLP0 PE=1 SV=1;sp|P05388|RLA0_HUMAN 60S acidic ribosomal protein P0 OS=Homo sapiens OX=9606 GN=RPLP0 PE=1 SV=1;tr|G3V210|G3V210_HUMAN 60S acidic ribosomal protein P0 OS=Homo	F8VWS0	RPLP0	60S acidic ribosomal protein P0	131000086.5	7	27.4	30.456	60.32
sp|Q15393|SF3B3_HUMAN Splicing factor 3B subunit 3 OS=Homo sapiens OX=9606 GN=SF3B3 PE=1 SV=4	Q15393	SF3B3	Splicing factor 3B subunit 3	18331008.47	6	7.4	135.58	57.777
tr|E7ENQ1|E7ENQ1_HUMAN Mitogen-activated protein kinase kinase kinase kinase 4 OS=Homo sapiens OX=9606 GN=MAP4K4 PE=1 SV=1;tr|H7C360|H7C360_HUMAN Mitogen-activated protein kinase kinase kinase kinase 4 (Fragment) OS=Homo sapiens OX=9606 GN=MAP4K4 PE=1 SV=1	E7ENQ1	MAP4K4	Mitogen-activated protein kinase kinase kinase kinase 4	36207017.51	9	9.3	132.16	56.345
tr|A0A6I8PIX7|A0A6I8PIX7_HUMAN Supervillin OS=Homo sapiens OX=9606 GN=SVIL PE=1 SV=1;sp|O95425|SVIL_HUMAN Supervillin OS=Homo sapiens OX=9606 GN=SVIL PE=1 SV=2	A0A6I8PIX7	SVIL	Supervillin	48952006.31	7	6	213.63	55.975

sp|P15924|DESP_HUMAN Desmoplakin OS=Homo sapiens OX=9606 GN=DSP PE=1 SV=3	P15924	DSP	Desmoplakin	21106003	9	2.9	331.77	55.806
tr|A0A3B3ITZ9|A0A3B3ITZ9_HUMAN Thyroid hormone receptor-associated protein 3 OS=Homo sapiens OX=9606 GN=THRAP3 PE=1 SV=1;sp|Q9Y2W1|TR150_HUMAN Thyroid hormone receptor-associated protein 3 OS=Homo sapiens OX=9606 GN=THRAP3 PE=1 SV=2	A0A3B3ITZ9	THRAP3	Thyroid hormone receptor-associated protein 3	34778008.4	8	9.2	103.52	55.757
sp|Q92616|GCN1_HUMAN eIF-2-alpha kinase activator GCN1 OS=Homo sapiens OX=9606 GN=GCN1 PE=1 SV=6	Q92616	GCN1	eIF-2-alpha kinase activator GCN1	8703599.219	8	4.2	292.75	53.881
sp|Q69YQ0|CYTSA_HUMAN Cytospin-A OS=Homo sapiens OX=9606 GN=SPECC1L PE=1 SV=2;tr|A0A494C1J1|A0A494C1J1_HUMAN Cytospin-A OS=Homo sapiens OX=9606 GN=SPECC1L PE=1 SV=1;tr|F8WAN1|F8WAN1_HUMAN Cytospin-A OS=Homo sapiens OX=9606 GN=SPECC1L-ADORA2A PE=3 SV=2;tr|C	Q69YQ0	SPECC1L	Cytospin-A	41656008.07	7	8.6	124.6	53.77
sp|Q96N67|DOCK7_HUMAN Dedicator of cytokinesis protein 7 OS=Homo sapiens OX=9606 GN=DOCK7 PE=1 SV=4;tr|A0A1B0GWE0|A0A1B0GWE0_HUMAN Dedicator of cytokinesis protein 7 (Fragment) OS=Homo sapiens OX=9606 GN=DOCK7 PE=1 SV=1	Q96N67	DOCK7	Dedicator of cytokinesis protein 7	33890007.33	8	4.7	242.56	52.38
sp|Q13435|SF3B2_HUMAN Splicing factor 3B subunit 2 OS=Homo sapiens OX=9606 GN=SF3B2 PE=1 SV=2;tr|E9PPJ0|E9PPJ0_HUMAN Splicing	Q13435	SF3B2	Splicing factor 3B subunit 2	23847988.9	6	7.9	100.23	52.295

factor 3B subunit 2 OS=Homo sapiens OX=9606 GN=SF3B2 PE=1 SV=1;tr|H0YCG1|H0YCG1_HUMAN Splicing factor 3B subunit 2 (Fragment) OS=H								
tr|F8VZJ2|F8VZJ2_HUMAN Nascent polypeptide-associated complex subunit alpha OS=Homo sapiens OX=9606 GN=NACA PE=1 SV=1;tr|F8VNW4|F8VNW4_HUMAN Nascent polypeptide-associated complex subunit alpha (Fragment) OS=Homo sapiens OX=9606 GN=NACA PE=1 SV=1;tr|F8W0W4	F8VZJ2	NACA	Nascent polypeptide-associated complex subunit alpha	39875008.09	2	21.3	15.016	52.254
sp|Q9Y2A7|NCKP1_HUMAN Nck-associated protein 1 OS=Homo sapiens OX=9606 GN=NCKAP1 PE=1 SV=1	Q9Y2A7	NCKAP1	Nck-associated protein 1	8241905.164	8	7.6	128.79	50.637
tr|A0A804HJ47|A0A804HJ47_HUMAN Aminoacyl tRNA synthase complex-interacting multifunctional protein 1 OS=Homo sapiens OX=9606 GN=AIMP1 PE=4 SV=1;tr|A0A5F9ZHC5|A0A5F9ZHC5_HUMAN Aminoacyl tRNA synthase complex-interacting multifunctional protein 1 OS=Homo sap	A0A804HJ47	AIMP1	Aminoacyl tRNA synthase complex-interacting multifunctional protein 1	52927024.76	7	25.9	28.828	50.607
tr|H3BPE1|H3BPE1_HUMAN Microtubule-actin cross-linking factor 1, isoforms 1/2/3/5 OS=Homo sapiens OX=9606 GN=MACF1 PE=1 SV=1;tr|H3BQK9|H3BQK9_HUMAN Microtubule-actin cross-linking factor 1, isoforms 1/2/3/5 OS=Homo sapiens OX=9606 GN=MACF1 PE=1 SV=1;tr|A0A	H3BPE1	MACF1	Microtubule-actin cross-linking factor 1, isoforms 1/2/3/5	11638998.66	8	1.2	856.87	48.291
tr|A0A7I2V5M7|A0A7I2V5M7_HUMAN Ras GTPase-activating protein-binding protein 1 OS=Homo sapiens OX=9606 GN=G3BP1 PE=1 SV=1;sp|Q13283|G3BP1_HUMAN Ras GTPase-activating protein-binding protein 1	A0A7I2V5M7	G3BP1	Ras GTPase-activating protein-binding protein 1	7866702.813	6	17	53.329	48.231

OS=Homo sapiens OX=9606 GN=G3BP1 PE=1 SV=1;tr|A0A7I2YQN9|A0A7I2Y								
sp|Q7Z460|CLAP1_HUMAN CLIP-associating protein 1 OS=Homo sapiens OX=9606 GN=CLASP1 PE=1 SV=1;tr|F8WA11|F8WA11_HUMAN CLIP-associating protein 1 OS=Homo sapiens OX=9606 GN=CLASP1 PE=1 SV=2;tr|H0Y5T1|H0Y5T1_HUMAN CLIP-associating protein 1 (Fragment) OS=Homo	Q7Z460	CLASP 1	CLIP-associating protein 1	25814995.47	9	7.9	169.45	46.075
sp|Q3ZCQ8|TIM50_HUMAN Mitochondrial import inner membrane translocase subunit TIM50 OS=Homo sapiens OX=9606 GN=TIMM50 PE=1 SV=2;tr|M0R0C3|M0R0C3_HUMAN Mitochondrial import inner membrane translocase subunit TIM50 (Fragment) OS=Homo sapiens OX=9606 GN=TIMM5	Q3ZCQ8	TIMM5 0	Mitochondrial import inner membrane translocase subunit TIM50	24025999.42	6	21.2	39.646	45.428
sp|P16070|CD44_HUMAN CD44 antigen OS=Homo sapiens OX=9606 GN=CD44 PE=1 SV=3;tr|H0YD13|H0YD13_HUMAN CD44 antigen OS=Homo sapiens OX=9606 GN=CD44 PE=1 SV=2;tr|H0Y2P0|H0Y2P0_HUMAN CD44 antigen (Fragment) OS=Homo sapiens OX=9606 GN=CD44 PE=1 SV=1;tr|H0YDW7|H0Y	P16070	CD44	CD44 antigen	18510003.52	7	10.2	81.537	44.815
sp|P81605|DCD_HUMAN Dermcidin OS=Homo sapiens OX=9606 GN=DCD PE=1 SV=2	P81605	DCD	Dermcidin	141220078.2	6	40.9	11.284	44.747
sp|Q02413|DSG1_HUMAN Desmoglein-1 OS=Homo sapiens OX=9606 GN=DSG1 PE=1 SV=2	Q02413	DSG1	Desmoglein-1	17826010.75	5	7.8	113.75	43.185

sp|P51398|RT29_HUMAN 28S ribosomal protein S29, mitochondrial OS=Homo sapiens OX=9606 GN=DAP3 PE=1 SV=1;tr|V9GYL9|V9GYL9_HUMAN 28S ribosomal protein S29, mitochondrial (Fragment) OS=Homo sapiens OX=9606 GN=DAP3 PE=1 SV=1;tr|V9GZ03|V9GZ03_HUMAN 28S ribosoma	P51398	DAP3	28S ribosomal protein S29, mitochondrial	12271993.36	6	19.3	45.566	42.94
sp|P01040|CYTA_HUMAN Cystatin-A OS=Homo sapiens OX=9606 GN=CSTA PE=1 SV=1;tr|C9J0E4|C9J0E4_HUMAN Cystatin-A OS=Homo sapiens OX=9606 GN=CSTA PE=1 SV=1	P01040	CSTA	Cystatin-A	19925988.56	6	68.4	11.006	42.388
sp|Q3KQU3|MA7D1_HUMAN MAP7 domain-containing protein 1 OS=Homo sapiens OX=9606 GN=MAP7D1 PE=1 SV=1;tr|D3DPS3|D3DPS3_HUMAN Arginine/proline rich coiled-coil 1, isoform CRA_b OS=Homo sapiens OX=9606 GN=MAP7D1 PE=3 SV=1	Q3KQU3	MAP7D1	MAP7 domain-containing protein 1	3048501.947	6	8.7	92.819	41.609
tr|D6R9P3|D6R9P3_HUMAN Heterogeneous nuclear ribonucleoprotein A/B OS=Homo sapiens OX=9606 GN=HNRNPAB PE=1 SV=1;tr|D6RD18|D6RD18_HUMAN Heterogeneous nuclear ribonucleoprotein A/B OS=Homo sapiens OX=9606 GN=HNRNPAB PE=1 SV=1;tr|D6RBZ0|D6RBZ0_HUMAN Heterogen	D6R9P3	HNRNPAB	Heterogeneous nuclear ribonucleoprotein A/B	67603980.49	6	18.6	30.302	41.299
sp|P28749|RBL1_HUMAN Retinoblastoma-like protein 1 OS=Homo sapiens OX=9606 GN=RBL1 PE=1 SV=3;tr|H0YE05|H0YE05_HUMAN Retinoblastoma-like protein 1 (Fragment)	P28749	RBL1	Retinoblastoma-like protein 1	13664993.14	6	5.4	120.85	40.119

OS=Homo sapiens OX=9606 GN=RBL1 PE=1 SV=8								
tr|A0A7I2V4N1|A0A7I2V4N1_HUMAN Heterogeneous nuclear ribonucleoproteins A2/B1 OS=Homo sapiens OX=9606 GN=HNRNPA2B1 PE=1 SV=1;tr|A0A024RA28|A0A024RA28_HUMAN Heterogeneous nuclear ribonucleoprotein A2/B1, isoform CRA_d OS=Homo sapiens OX=9606 GN=HNRNPA2B1 PE	A0A7I2 V4N1	HNRN PA2B1	Heterogeneous nuclear ribonucleoprot eins A2/B1	29099995. 81	6	24.3	32.487	39.23
sp|P98175|RBM10_HUMAN RNA-binding protein 10 OS=Homo sapiens OX=9606 GN=RBM10 PE=1 SV=3	P98175	RBM10	RNA-binding protein 10	25348013. 25	6	7.1	103.53	39.199
tr|A0A7I2V451|A0A7I2V451_HUMAN Cytospin-B OS=Homo sapiens OX=9606 GN=SPECC1 PE=1 SV=1;tr|A0A7I2YQJ3|A0A7I2YQJ3_HUMAN Cytospin-B OS=Homo sapiens OX=9606 GN=SPECC1 PE=1 SV=1;tr|A0A7P0TBP2|A0A7P0TBP2_HUMAN Cytospin-B OS=Homo sapiens OX=9606 GN=SPECC1 PE=1 SV=	A0A7I2 V451	SPECC 1	Cytospin-B	16486992. 38	6	6	112.04	39.019
sp|Q12906|ILF3_HUMAN Interleukin enhancer-binding factor 3 OS=Homo sapiens OX=9606 GN=ILF3 PE=1 SV=3	Q12906	ILF3	Interleukin enhancer-binding factor 3	15271999. 29	6	7.3	95.337	38.284
sp|Q9BYX2|TBD2A_HUMAN TBC1 domain family member 2A OS=Homo sapiens OX=9606 GN=TBC1D2 PE=1 SV=3	Q9BYX 2	TBC1D 2	TBC1 domain family member 2A	10232000. 15	5	6.9	105.41	37.825
tr|D6RF44|D6RF44_HUMAN Heterogeneous nuclear ribonucleoprotein D0 (Fragment) OS=Homo sapiens OX=9606 GN=HNRNPD PE=1 SV=8;tr|H0YA96|H0YA96_HUMAN Heterogeneous nuclear ribonucleoprotein D0	D6RF44	HNRN PD	Heterogeneous nuclear ribonucleoprot ein D0 (Fragment)	40815013. 2	4	35.1	12.553	37.739

(Fragment) OS=Homo sapiens OX=9606 GN=HNRNPD PE=1 SV=1;tr|D6RAF8|D6RA								
sp|O94851|MICA2_HUMAN [F-actin]-monooxygenase MICAL2 OS=Homo sapiens OX=9606 GN=MICAL2 PE=1 SV=1;tr|A0A2R8YFA9|A0A2R8YFA9_HUMAN F-actin monooxygenase OS=Homo sapiens OX=9606 GN=MICAL2 PE=1 SV=1	O94851	MICAL2	[F-actin]-monooxygenase MICAL2	6733896.429	5	5.2	126.69	37.639
sp|Q7L7X3|TAOK1_HUMAN Serine/threonine-protein kinase TAO1 OS=Homo sapiens OX=9606 GN=TAOK1 PE=1 SV=1	Q7L7X3	TAOK1	Serine/threonine-protein kinase TAO1	18421993.37	6	6.7	116.07	37.217
sp|Q12965|MYO1E_HUMAN Unconventional myosin-Ie OS=Homo sapiens OX=9606 GN=MYO1E PE=1 SV=2	Q12965	MYO1E	Unconventional myosin-Ie	8631898.166	5	5.3	127.06	36.123
sp|Q6VY07|PACS1_HUMAN Phosphofurin acidic cluster sorting protein 1 OS=Homo sapiens OX=9606 GN=PACS1 PE=1 SV=2;tr|B4DF77|B4DF77_HUMAN Phosphofurin acidic cluster sorting protein 1 OS=Homo sapiens OX=9606 GN=PACS1 PE=1 SV=1	Q6VY07	PACS1	Phosphofurin acidic cluster sorting protein 1	6249997.553	5	6.2	104.9	35.394
tr|A0A2R8Y6J3|A0A2R8Y6J3_HUMAN 60S ribosomal protein L5 (Fragment) OS=Homo sapiens OX=9606 GN=RPL5 PE=1 SV=1;sp|P46777|RL5_HUMAN 60S ribosomal protein L5 OS=Homo sapiens OX=9606 GN=RPL5 PE=1 SV=3;tr|Q5T7N0|Q5T7N0_HUMAN 60S ribosomal protein L5 (Fragment) O	A0A2R8Y6J3	RPL5	60S ribosomal protein L5 (Fragment)	50366007.47	5	29.8	27.045	34.897
tr|J3QQV2|J3QQV2_HUMAN Monocarboxylate transporter 4 OS=Homo sapiens OX=9606 GN=SLC16A3 PE=1 SV=9;tr|J3KTM6|J3KTM6_HUMAN Monocarboxylate transporter 4 OS=Homo	J3QQV2	SLC16A3	Monocarboxylate transporter 4	26347994.24	5	12.5	44.478	34.52

sapiens OX=9606 GN=SLC16A3 PE=1 SV=2;sp|O15427|MOT4_HUMAN Monocarboxylate transporter 4 OS=Homo s								
tr|F8VV32|F8VV32_HUMAN 1,4-beta-N-acetylmuramidase C OS=Homo sapiens OX=9606 GN=LYZ PE=1 SV=1;tr|A0A0B4J259|A0A0B4J259_HUMAN 1,4-beta-N-acetylmuramidase C OS=Homo sapiens OX=9606 GN=LYZ PE=1 SV=1;sp|P61626|LYSC_HUMAN Lysozyme C OS=Homo sapiens OX=9606 GN=L	F8VV32	LYZ	1,4-beta-N-acetylmuramidase C	20663003.61	2	20.2	11.488	33.124
sp|Q53EP0|FND3B_HUMAN Fibronectin type III domain-containing protein 3B OS=Homo sapiens OX=9606 GN=FNDC3B PE=1 SV=2	Q53EP0	FNDC3B	Fibronectin type III domain-containing protein 3B	14630995.18	5	5.8	132.89	32.999
sp|P05109|S10A8_HUMAN Protein S100-A8 OS=Homo sapiens OX=9606 GN=S100A8 PE=1 SV=1	P05109	S100A8	Protein S100-A8	27275985.85	3	31.2	10.834	32.586
sp|O75165|DJC13_HUMAN DnaJ homolog subfamily C member 13 OS=Homo sapiens OX=9606 GN=DNAJC13 PE=1 SV=5	O75165	DNAJC13	DnaJ homolog subfamily C member 13	8256901.344	5	2.9	254.41	32.314
sp|Q9NQW6|ANLN_HUMAN Anillin OS=Homo sapiens OX=9606 GN=ANLN PE=1 SV=2	Q9NQW6	ANLN	Anillin	20897013.55	5	5	124.2	32.313
sp|P61619|S61A1_HUMAN Protein transport protein Sec61 subunit alpha isoform 1 OS=Homo sapiens OX=9606 GN=SEC61A1 PE=1 SV=2;tr|B4DR61|B4DR61_HUMAN Protein transport protein Sec61 subunit alpha isoform 1 OS=Homo sapiens OX=9606 GN=SEC61A1 PE=1 SV=1	P61619	SEC61A1	Protein transport protein Sec61 subunit alpha isoform 1	32398009.79	5	10.1	52.264	31.504
sp|P0CG48|UBC_HUMAN Polyubiquitin-C OS=Homo sapiens OX=9606 GN=UBC PE=1	P0CG48	UBC	Polyubiquitin-C	49178004.4	3	44.7	77.038	31.108

SV=3;tr|B4DV12|B4DV12_HUMAN Polyubiquitin-B OS=Homo sapiens OX=9606 GN=UBB PE=1 SV=1;tr|Q96C32|Q96C32_HUMAN Polyubiquitin-C OS=Homo sapiens OX=9606 GN=UBC PE=1 SV=1;sp|P0CG47|UBB_HUMAN								
sp|Q5T749|KPRP_HUMAN Keratinocyte proline-rich protein OS=Homo sapiens OX=9606 GN=KPRP PE=1 SV=1	Q5T749	KPRP	Keratinocyte proline-rich protein	20546005.26	5	10	64.135	30.715
tr|A0A1W2PRZ0|A0A1W2PRZ0_HUMAN HLA class I histocompatibility antigen, C alpha chain OS=Homo sapiens OX=9606 GN=HLA-C PE=1 SV=1;tr|A0A1W2PRX1|A0A1W2PRX1_HUMAN HLA class I histocompatibility antigen, C alpha chain OS=Homo sapiens OX=9606 GN=HLA-C PE=1 SV=1;	A0A1W2PRZ0	HLA-C	HLA class I histocompatibility antigen, C alpha chain	2434800.414	4	18.8	36.725	27.586
tr|Q5QPR4|Q5QPR4_HUMAN Cyclin-dependent kinase 11A OS=Homo sapiens OX=9606 GN=CDK11A PE=1 SV=1;tr|Q5QPR3|Q5QPR3_HUMAN Cyclin-dependent kinase 11A OS=Homo sapiens OX=9606 GN=CDK11A PE=1 SV=1;sp|Q9UQ88|CD11A_HUMAN Cyclin-dependent kinase 11A OS=Homo sapiens	Q5QPR4	CDK11A	Cyclin-dependent kinase 11A	5437599.338	4	6.4	86.823	27.465
sp|Q9P2E3|ZNFX1_HUMAN NFX1-type zinc finger-containing protein 1 OS=Homo sapiens OX=9606 GN=ZNFX1 PE=2 SV=2;tr|Q5JXR5|Q5JXR5_HUMAN NFX1-type zinc finger-containing protein 1 (Fragment) OS=Homo sapiens OX=9606 GN=ZNFX1 PE=1 SV=1;tr|Q5JXR6|Q5JXR6_HUMAN NFX1-	Q9P2E3	ZNFX1	NFX1-type zinc finger-containing protein 1	5274401.416	4	2.5	220.22	27.433

sp|P31151|S10A7_HUMAN Protein S100-A7 OS=Homo sapiens OX=9606 GN=S100A7 PE=1 SV=4;sp|Q86SG5|S1A7A_HUMAN Protein S100-A7A OS=Homo sapiens OX=9606 GN=S100A7A PE=1 SV=3	P31151	S100A7	Protein S100-A7	10461001.82	4	36.6	11.471	27.144
sp|Q5JU85|IQEC2_HUMAN IQ motif and SEC7 domain-containing protein 2 OS=Homo sapiens OX=9606 GN=IQSEC2 PE=1 SV=2;tr|A0A6Q8PFR7|A0A6Q8PFR7_HUMAN IQ motif and SEC7 domain-containing protein 2 OS=Homo sapiens OX=9606 GN=IQSEC2 PE=1 SV=1;tr|A0A1W2PR28|A0A1W2PR2	Q5JU85	IQSEC2	IQ motif and SEC7 domain-containing protein 2	9292297.986	4	2.6	162.78	26.977
sp|Q96EY1|DNJA3_HUMAN DnaJ homolog subfamily A member 3, mitochondrial OS=Homo sapiens OX=9606 GN=DNAJA3 PE=1 SV=2	Q96EY1	DNAJA3	DnaJ homolog subfamily A member 3, mitochondrial	19293997.01	4	11.7	52.488	26.187
sp|Q9BZ29|DOCK9_HUMAN Dedicator of cytokinesis protein 9 OS=Homo sapiens OX=9606 GN=DOCK9 PE=1 SV=2;tr|A0A088AWN3|A0A088AWN3_HUMAN Dedicator of cytokinesis protein 9 OS=Homo sapiens OX=9606 GN=DOCK9 PE=1 SV=2;tr|A0A804HIE8|A0A804HIE8_HUMAN Dedicator of cyt	Q9BZ29	DOCK9	Dedicator of cytokinesis protein 9	21281010.99	4	2.4	236.44	25.977
sp|Q86Y46|K2C73_HUMAN Keratin, type II cytoskeletal 73 OS=Homo sapiens OX=9606 GN=KRT73 PE=1 SV=1;sp|Q7RTS7|K2C74_HUMAN Keratin, type II cytoskeletal 74 OS=Homo sapiens OX=9606 GN=KRT74 PE=1 SV=2;tr|F8W1S1|F8W1S1_HUMAN Keratin, type II cytoskeletal 74 OS=H	Q86Y46	KRT73	Keratin, type II cytoskeletal 73	18012001.15	9	12.2	58.923	24.889

sp|Q08554|DSC1_HUMAN Desmocollin-1 OS=Homo sapiens OX=9606 GN=DSC1 PE=1 SV=2	Q08554	DSC1	Desmocollin-1	11609998.43	4	5.5	99.986	24.18
sp|P14923|PLAK_HUMAN Junction plakoglobin OS=Homo sapiens OX=9606 GN=JUP PE=1 SV=3;tr|C9JTX4|C9JTX4_HUMAN Junction plakoglobin (Fragment) OS=Homo sapiens OX=9606 GN=JUP PE=1 SV=1;tr|C9J826|C9J826_HUMAN Junction plakoglobin (Fragment) OS=Homo sapiens OX=960	P14923	JUP	Junction plakoglobin	7108097.818	4	4.8	81.744	24.01
sp|Q92620|PRP16_HUMAN Pre-mRNA-splicing factor ATP-dependent RNA helicase PRP16 OS=Homo sapiens OX=9606 GN=DHX38 PE=1 SV=2	Q92620	DHX38	Pre-mRNA-splicing factor ATP-dependent RNA helicase PRP16	2141900.984	3	2.9	140.5	23.742
tr|A6NML8|A6NML8_HUMAN Diaphanous homolog 2 (Drosophila), isoform CRA_c OS=Homo sapiens OX=9606 GN=DIAPH2 PE=1 SV=3;tr|K4DI95|K4DI95_HUMAN Diaphanous homolog 2 (Drosophila), isoform CRA_a OS=Homo sapiens OX=9606 GN=DIAPH2 PE=1 SV=1;sp|O60879|DIAP2_HUMAN Pr	A6NML8	DIAPH2	Diaphanous homolog 2 (Drosophila), isoform CRA	4711699.703	4	3.7	124.85	23.55
sp|Q13085|ACACA_HUMAN Acetyl-CoA carboxylase 1 OS=Homo sapiens OX=9606 GN=ACACA PE=1 SV=2;tr|Q59FY4|Q59FY4_HUMAN Acetyl-CoA carboxylase 1 (Fragment) OS=Homo sapiens OX=9606 GN=ACACA PE=1 SV=1;sp|O00763|ACACB_HUMAN Acetyl-CoA carboxylase 2 OS=Homo sapiens O	Q13085	ACACA	Acetyl-CoA carboxylase 1	5900098.72	4	2.2	265.55	23.437

tr|A0A6Q8PFN8|A0A6Q8PFN8_HUMAN IkappaB kinase complex-associated protein OS=Homo sapiens OX=9606 GN=ELP1 PE=1 SV=1;tr|F5H2T0|F5H2T0_HUMAN IkappaB kinase complex-associated protein OS=Homo sapiens OX=9606 GN=ELP1 PE=1 SV=1;tr|A0A6Q8PH48|A0A6Q8PH48_HUMAN Ika	A0A6Q8PFN8	ELP1	IkappaB kinase complex-associated protein	4978600.184	3	4.5	103.97	23.08
sp|Q5T6F2|UBAP2_HUMAN Ubiquitin-associated protein 2 OS=Homo sapiens OX=9606 GN=UBAP2 PE=1 SV=1	Q5T6F2	UBAP2	Ubiquitin-associated protein 2	11678998.08	3	4.4	117.11	22.789
tr|A0A6Q8KRG2|A0A6Q8KRG2_HUMAN Golgin subfamily A member 2 OS=Homo sapiens OX=9606 GN=GOLGA2 PE=1 SV=1;sp|Q08379|GOGA2_HUMAN Golgin subfamily A member 2 OS=Homo sapiens OX=9606 GN=GOLGA2 PE=1 SV=3	A0A6Q8KRG2	GOLGA2	Golgin subfamily A member 2	11835992.89	3	3.3	111.66	22.716
sp|Q2NL82|TSR1_HUMAN Pre-rRNA-processing protein TSR1 homolog OS=Homo sapiens OX=9606 GN=TSR1 PE=1 SV=1;tr|I3L1Q5|I3L1Q5_HUMAN Pre-rRNA-processing protein TSR1 homolog OS=Homo sapiens OX=9606 GN=TSR1 PE=1 SV=2	Q2NL82	TSR1	Pre-rRNA-processing protein TSR1 homolog	5105999.544	3	5	91.809	22.342
sp|P53814|SMTN_HUMAN Smoothelin OS=Homo sapiens OX=9606 GN=SMTN PE=1 SV=7;tr|A0A087WVP4|A0A087WVP4_HUMAN Smoothelin OS=Homo sapiens OX=9606 GN=SMTN PE=1 SV=1;tr|A0A087X1R1|A0A087X1R1_HUMAN Smoothelin OS=Homo sapiens OX=9606 GN=SMTN PE=1 SV=1	P53814	SMTN	Smoothelin	5805298.532	3	3.8	99.058	21.779
sp|Q3SY84|K2C71_HUMAN Keratin, type II cytoskeletal 71 OS=Homo sapiens OX=9606 GN=KRT71 PE=1 SV=3	Q3SY84	KRT71	Keratin, type II cytoskeletal 71	12910006.48	8	13.2	57.291	21.693

sp|Q29RF7|PDS5A_HUMAN Sister chromatid cohesion protein PDS5 homolog A OS=Homo sapiens OX=9606 GN=PDS5A PE=1 SV=1;tr|H0Y9L9|H0Y9L9_HUMAN Sister chromatid cohesion protein PDS5 homolog A (Fragment) OS=Homo sapiens OX=9606 GN=PDS5A PE=1 SV=1;sp|Q9NTI5|PDS5B_	Q29RF7	PDS5A	Sister chromatid cohesion protein PDS5 homolog A	1484100.911	2	1.9	150.83	20.795
tr|Q5QPM0|Q5QPM0_HUMAN RNA-binding protein Raly (Fragment) OS=Homo sapiens OX=9606 GN=RALY PE=1 SV=8;tr|Q5QPL9|Q5QPL9_HUMAN RNA-binding protein Raly (Fragment) OS=Homo sapiens OX=9606 GN=RALY PE=1 SV=1;sp|Q9UKM9|RALY_HUMAN RNA-binding protein Raly OS=Homo	Q5QPM0	RALY	RNA-binding protein Raly (Fragment)	9044194.34	3	17.5	18.66	20.641
sp|Q9H4L5|OSBL3_HUMAN Oxysterol-binding protein-related protein 3 OS=Homo sapiens OX=9606 GN=OSBPL3 PE=1 SV=1	Q9H4L5	OSBPL3	Oxysterol-binding protein-related protein 3	4269198.096	3	4.8	101.22	20.174
sp|Q9UH62|ARMX3_HUMAN Armadillo repeat-containing X-linked protein 3 OS=Homo sapiens OX=9606 GN=ARMCX3 PE=1 SV=1	Q9UH62	ARMCX3	Armadillo repeat-containing X-linked protein 3	5085700.726	3	9	42.5	19.998
sp|Q9NX05|F120C_HUMAN Constitutive coactivator of PPAR-gamma-like protein 2 OS=Homo sapiens OX=9606 GN=FAM120C PE=1 SV=3;tr|F8W881|F8W881_HUMAN Constitutive coactivator of PPAR-gamma-like protein 2 OS=Homo sapiens OX=9606 GN=FAM120C PE=1 SV=2	Q9NX05	FAM120C	Constitutive coactivator of PPAR-gamma-like protein 2	1149999.337	3	2.7	120.59	19.785
tr|A0A087WUK2|A0A087WUK2_HUMAN Heterogeneous nuclear ribonucleoprotein D-like OS=Homo sapiens OX=9606 GN=HNRNPDL	A0A087WUK2	HNRNPDL	Heterogeneous nuclear	11856995.53	4	12.4	40.04	19.5

PE=1 SV=1;sp|O14979|HNRDL_HUMAN Heterogeneous nuclear ribonucleoprotein D-like OS=Homo sapiens OX=9606 GN=HNRNPDL PE=1 SV=3			ribonucleoprotein D-like					
sp|P04156|PRIO_HUMAN Major prion protein OS=Homo sapiens OX=9606 GN=PRNP PE=1 SV=1;tr|A2A2V1|A2A2V1_HUMAN Major prion protein (Fragment) OS=Homo sapiens OX=9606 GN=PRNP PE=1 SV=1	P04156	PRNP	Major prion protein	49015017.58	3	12.3	27.661	19.209
sp|Q9UDT6|CLIP2_HUMAN CAP-Gly domain-containing linker protein 2 OS=Homo sapiens OX=9606 GN=CLIP2 PE=1 SV=1	Q9UDT6	CLIP2	CAP-Gly domain-containing linker protein 2	6205602.127	3	3.3	115.84	18.992
sp|P12273|PIP_HUMAN Prolactin-inducible protein OS=Homo sapiens OX=9606 GN=PIP PE=1 SV=1	P12273	PIP	Prolactin-inducible protein	6423598.006	3	25.3	16.572	18.76
sp|Q71RC2|LARP4_HUMAN La-related protein 4 OS=Homo sapiens OX=9606 GN=LARP4 PE=1 SV=3;tr|Q96J85|Q96J85_HUMAN C-Mpl binding protein OS=Homo sapiens OX=9606 GN=LARP4 PE=1 SV=1;tr|X6RLN4|X6RLN4_HUMAN La-related protein 4 (Fragment) OS=Homo sapiens OX=9606 GN=	Q71RC2	LARP4	La-related protein 4	9090899.336	3	4.3	80.595	18.62
tr|F8VUA7|F8VUA7_HUMAN Oxysterol-binding protein (Fragment) OS=Homo sapiens OX=9606 GN=OSBPL8 PE=1 SV=8;tr|F8VQX7|F8VQX7_HUMAN Oxysterol-binding protein (Fragment) OS=Homo sapiens OX=9606 GN=OSBPL8 PE=1 SV=1;sp|Q9BZF1|OSBL8_HUMAN Oxysterol-binding protein-	F8VUA7	OSBPL8	Oxysterol-binding protein (Fragment)	2887698.568	2	4.3	79.052	18.435
sp|Q7Z406|MYH14_HUMAN Myosin-14 OS=Homo sapiens OX=9606 GN=MYH14	Q7Z406	MYH14	Myosin-14	124509923.2	12	5.4	227.87	18.421

PE=1 SV=2;tr|M0QY43|M0QY43_HUMAN Myosin-14 (Fragment) OS=Homo sapiens OX=9606 GN=MYH14 PE=1 SV=8								
sp|Q04695|K1C17_HUMAN Keratin, type I cytoskeletal 17 OS=Homo sapiens OX=9606 GN=KRT17 PE=1 SV=2;tr|F5GWP8|F5GWP8_HUMAN Keratin, type I cytoskeletal 17 OS=Homo sapiens OX=9606 GN=KRT17 PE=1 SV=2;tr|K7EPJ9|K7EPJ9_HUMAN Keratin, type I cytoskeletal 17 (Fragm	Q04695	KRT17	Keratin, type I cytoskeletal 17	8875600.303	14	28.9	48.105	17.76
sp|Q8TF46|DI3L1_HUMAN DIS3-like exonuclease 1 OS=Homo sapiens OX=9606 GN=DIS3L PE=1 SV=2	Q8TF46	DIS3L	DIS3-like exonuclease 1	3251399.487	3	2.9	120.79	17.554
sp|Q01081|U2AF1_HUMAN Splicing factor U2AF 35 kDa subunit OS=Homo sapiens OX=9606 GN=U2AF1 PE=1 SV=3	Q01081	U2AF1	Splicing factor U2AF 35 kDa subunit	112140057.3	2	8.8	27.872	17.438
tr|A0A087WTV6|A0A087WTV6_HUMAN Pyrroline-5-carboxylate reductase OS=Homo sapiens OX=9606 GN=PYCR2 PE=1 SV=1;sp|Q96C36|P5CR2_HUMAN Pyrroline-5-carboxylate reductase 2 OS=Homo sapiens OX=9606 GN=PYCR2 PE=1 SV=1;tr|J3KR12|J3KR12_HUMAN Pyrroline-5-carboxylate	A0A087WTV6	PYCR2	Pyrroline-5-carboxylate reductase	10930997.84	2	12.2	25.868	17.342
sp|Q5D862|FILA2_HUMAN Filaggrin-2 OS=Homo sapiens OX=9606 GN=FLG2 PE=1 SV=1	Q5D862	FLG2	Filaggrin-2	14273994.22	2	1	248.07	17.151
tr|V9GYN7|V9GYN7_HUMAN DnaJ homolog subfamily B member 12 (Fragment) OS=Homo sapiens OX=9606 GN=DNAJB12 PE=1 SV=1;sp|Q9NXW2|DJB12_HUMAN DnaJ homolog subfamily B member 12 OS=Homo sapiens OX=9606 GN=DNAJB12 PE=1	V9GYN7	DNAJB12	DnaJ homolog subfamily B member 12 (Fragment)	7865600.452	2	9.1	31.12	17.133

SV=5;tr|J3KPS0|J3KPS0_HUMAN DnaJ (Hsp40) homo								
sp|Q7Z3Y8|K1C27_HUMAN Keratin, type I cytoskeletal 27 OS=Homo sapiens OX=9606 GN=KRT27 PE=1 SV=2;sp|Q7Z3Z0|K1C25_HUMAN Keratin, type I cytoskeletal 25 OS=Homo sapiens OX=9606 GN=KRT25 PE=1 SV=1	Q7Z3Y8	KRT27	Keratin, type I cytoskeletal 27	12177993.67	5	12.2	49.822	17.132
tr|Q5VWU8|Q5VWU8_HUMAN Partitioning defective 3 homolog OS=Homo sapiens OX=9606 GN=PARD3 PE=1 SV=1;tr|Q5VWV2|Q5VWV2_HUMAN Partitioning defective 3 homolog OS=Homo sapiens OX=9606 GN=PARD3 PE=1 SV=1;sp|Q8TEW0|PARD3_HUMAN Partitioning defective 3 homolog OS=	Q5VWU8	PARD3	Partitioning defective 3 homolog	8402804.563	2	2.4	110	17.034
sp|P62995|TRA2B_HUMAN Transformer-2 protein homolog beta OS=Homo sapiens OX=9606 GN=TRA2B PE=1 SV=1;tr|H7C2L4|H7C2L4_HUMAN Transformer-2 protein homolog beta (Fragment) OS=Homo sapiens OX=9606 GN=TRA2B PE=1 SV=1	P62995	TRA2B	Transformer-2 protein homolog beta	30780982.9	2	9	33.665	16.706
sp|P31944|CASPE_HUMAN Caspase-14 OS=Homo sapiens OX=9606 GN=CASP14 PE=1 SV=2	P31944	CASP14	Caspase-14	1896800.965	2	9.9	27.679	15.3
tr|H0YH87|H0YH87_HUMAN Ataxin-2 OS=Homo sapiens OX=9606 GN=ATXN2 PE=1 SV=2;tr|A0A2R8Y7P6|A0A2R8Y7P6_HUMAN Ataxin-2 OS=Homo sapiens OX=9606 GN=ATXN2 PE=1 SV=1;tr|A0A2R8YDM9|A0A2R8YDM9_HUM	H0YH87	ATXN2	Ataxin-2	6620296.641	2	3.8	90.166	14.578

AN Ataxin-2 OS=Homo sapiens OX=9606 GN=ATXN2 PE=1 SV=1;tr|A0A5F9ZHH4|A								
tr|C9J8R7|C9J8R7_HUMAN GTP-binding protein 10 (Fragment) OS=Homo sapiens OX=9606 GN=GTPBP10 PE=1 SV=1;tr|C9JNI1|C9JNI1_HUMAN GTP-binding protein 10 (Fragment) OS=Homo sapiens OX=9606 GN=GTPBP10 PE=1 SV=1;sp|A4D1E9|GTPBA_HUMAN GTP-binding protein 10 OS=Homo	C9J8R7	GTPBP 10	GTP-binding protein 10 (Fragment)	3717101.2	2	11.4	22.475	14.135
sp|P35249|RFC4_HUMAN Replication factor C subunit 4 OS=Homo sapiens OX=9606 GN=RFC4 PE=1 SV=2;tr|H7C1P0|H7C1P0_HUMAN Replication factor C subunit 4 (Fragment) OS=Homo sapiens OX=9606 GN=RFC4 PE=1 SV=1;tr|C9JZI1|C9JZI1_HUMAN Replication factor C subunit 4 O	P35249	RFC4	Replication factor C subunit 4	6654801.1 46	2	6.6	39.681	13.904
sp|P55042|RAD_HUMAN GTP-binding protein RAD OS=Homo sapiens OX=9606 GN=RRAD PE=1 SV=2;tr|J3KRG9|J3KRG9_HUMAN GTP-binding protein RAD (Fragment) OS=Homo sapiens OX=9606 GN=RRAD PE=1 SV=1;tr|J3KSM6|J3KSM6_HUMAN GTP-binding protein RAD (Fragment) OS=Homo sapi	P55042	RRAD	GTP-binding protein RAD	20353994. 17	2	7.8	33.245	13.816
sp|P25311|ZA2G_HUMAN Zinc-alpha-2-glycoprotein OS=Homo sapiens OX=9606 GN=AZGP1 PE=1 SV=2;tr|C9JEV0|C9JEV0_HUMAN Zinc-alpha-2-glycoprotein OS=Homo sapiens OX=9606 GN=AZGP1 PE=1 SV=1	P25311	AZGP1	Zinc-alpha-2-glycoprotein	11033999. 61	2	8.7	34.258	13.756
sp|O15042|SR140_HUMAN U2 snRNP-associated SURP motif-containing protein OS=Homo sapiens OX=9606 GN=U2SURP	O15042	U2SUR P	U2 snRNP-associated SURP motif-	4587401.5 25	2	1.9	118.29	13.683

PE=1 SV=2;tr|E7ET15|E7ET15_HUMAN U2 snRNP-associated SURP motif-containing protein OS=Homo sapiens OX=9606 GN=U2SURP PE=1 SV=1			containing protein					
sp|Q13751|LAMB3_HUMAN Laminin subunit beta-3 OS=Homo sapiens OX=9606 GN=LAMB3 PE=1 SV=1;tr|X1WI29|X1WI29_HUMAN Laminin subunit beta-3 (Fragment) OS=Homo sapiens OX=9606 GN=LAMB3 PE=1 SV=7	Q13751	LAMB 3	Laminin subunit beta-3	4659497.4 64	2	2	129.57	13.445
tr|F5GX09|F5GX09_HUMAN Protein FAM76B OS=Homo sapiens OX=9606 GN=FAM76B PE=1 SV=1;sp|Q5HYJ3|FA76B_HUMAN Protein FAM76B OS=Homo sapiens OX=9606 GN=FAM76B PE=1 SV=3	F5GX09	FAM76 B	Protein FAM76B	12825999. 33	2	5.6	38.62	13.426
tr|A0A075B7B5|A0A075B7B5_HUMAN SLIT-ROBO Rho GTPase-activating protein 2 OS=Homo sapiens OX=9606 GN=SRGAP2 PE=1 SV=1;tr|G5EA48|G5EA48_HUMAN SLIT-ROBO Rho GTPase activating protein 1, isoform CRA_a OS=Homo sapiens OX=9606 GN=SRGAP1 PE=1 SV=1;tr|B7ZM87|B7ZM8	A0A075 B7B5	SRGAP 2	SLIT-R	4611798.4 74	2	2.8	90.908	13.346
sp|Q15678|PTN14_HUMAN Tyrosine-protein phosphatase non-receptor type 14 OS=Homo sapiens OX=9606 GN=PTPN14 PE=1 SV=2	Q15678	PTPN1 4	Tyrosine-protein phosphatase non-receptor type 14	3772401.0 28	2	1.7	135.26	13.263
tr|A8MYV2|A8MYV2_HUMAN LUC7-like (S. cerevisiae) OS=Homo sapiens OX=9606 GN=LUC7L PE=1 SV=1;sp|Q9NQ29|LUC7L_HUMAN Putative RNA-binding protein Luc7-like 1 OS=Homo sapiens OX=9606 GN=LUC7L PE=1	A8MYV 2	LUC7L	LUC7-like (S. cerevisiae)	8752004.3 35	2	6.6	32.447	13.199

SV=1;sp|Q9Y383|LC7L2_HUMAN Putative RNA-binding protein Luc7-li								
tr|K7EQG1|K7EQG1_HUMAN Glutaminyl-peptide cyclotransferase OS=Homo sapiens OX=9606 GN=QPCTL PE=1 SV=1;tr|A0A7I2V5X1|A0A7I2V5X1_HUMAN Glutaminyl-peptide cyclotransferase OS=Homo sapiens OX=9606 GN=QPCTL PE=1 SV=1;sp|Q9NXS2|QPCTL_HUMAN Glutaminyl-peptide cyc	K7EQG1	QPCTL	Glutaminyl-peptide cyclotransferase	14072004.75	2	18.2	13.98	13.196
tr|H0Y9H2|H0Y9H2_HUMAN Alpha-adducin (Fragment) OS=Homo sapiens OX=9606 GN=ADD1 PE=1 SV=1;tr|E7EV99|E7EV99_HUMAN Alpha-adducin OS=Homo sapiens OX=9606 GN=ADD1 PE=1 SV=1;tr|E7ENY0|E7ENY0_HUMAN Alpha-adducin OS=Homo sapiens OX=9606 GN=ADD1 PE=1 SV=1;sp|P3561	H0Y9H2	ADD1	Alpha-adducin (Fragment)	4573001.885	2	6.8	48.407	13.175
tr|H0YGW7|H0YGW7_HUMAN ATP-binding cassette sub-family F member 1 (Fragment) OS=Homo sapiens OX=9606 GN=ABCF1 PE=1 SV=1;sp|Q8NE71|ABCF1_HUMAN ATP-binding cassette sub-family F member 1 OS=Homo sapiens OX=9606 GN=ABCF1 PE=1 SV=2;tr|Q5STZ8|Q5STZ8_HUMAN ATP-b	H0YGW7	ABCF1	ATP-binding cassette sub-family F member 1 (Fragment)	2397199.882	2	3.9	67.55	13.162
sp|Q8N3C0|ASCC3_HUMAN Activating signal cointegrator 1 complex subunit 3 OS=Homo sapiens OX=9606 GN=ASCC3 PE=1 SV=3	Q8N3C0	ASCC3	Activating signal cointegrator 1 complex subunit 3	6848295.565	2	1.1	251.46	12.814
sp|Q8N1N4|K2C78_HUMAN Keratin, type II cytoskeletal 78 OS=Homo sapiens OX=9606 GN=KRT78 PE=1 SV=2	Q8N1N4	KRT78	Keratin, type II cytoskeletal 78	2731900.562	3	5.2	56.865	12.559

tr|H7C1W1|H7C1W1_HUMAN Peroxidasin homolog (Fragment) OS=Homo sapiens OX=9606 GN=PXDN PE=1 SV=1;sp|Q92626|PXDN_HUMAN Peroxidasin homolog OS=Homo sapiens OX=9606 GN=PXDN PE=1 SV=2	H7C1W1	PXDN	Peroxidasin homolog (Fragment)	1178300.296	2	4	79.755	12.532
tr|D6R9T3|D6R9T3_HUMAN Abasic site processing protein HMCES (Fragment) OS=Homo sapiens OX=9606 GN=HMCES PE=1 SV=1;tr|E7EMP6|E7EMP6_HUMAN Abasic site processing protein HMCES OS=Homo sapiens OX=9606 GN=HMCES PE=1 SV=1;sp|Q96FZ2|HMCES_HUMAN Abasic site proce	D6R9T3	HMCES	Abasic site processing protein HMCES (Fragment)	7804702.287	2	9.9	32.132	12.23
sp|Q96DI7|SNR40_HUMAN U5 small nuclear ribonucleoprotein 40 kDa protein OS=Homo sapiens OX=9606 GN=SNRNP40 PE=1 SV=1;tr|Q9NSS8|Q9NSS8_HUMAN U5 small nuclear ribonucleoprotein 40 kDa protein OS=Homo sapiens OX=9606 GN=SNRNP40 PE=1 SV=1	Q96DI7	SNRNP40	U5 small nuclear ribonucleoprotein 40 kDa protein	8063794.8	2	7.3	39.31	11.954
sp|P06702|S10A9_HUMAN Protein S100-A9 OS=Homo sapiens OX=9606 GN=S100A9 PE=1 SV=1	P06702	S100A9	Protein S100-A9	3732297.648	2	17.5	13.242	11.932
sp|Q96P63|SPB12_HUMAN Serpin B12 OS=Homo sapiens OX=9606 GN=SERPINB12 PE=1 SV=1	Q96P63	SERPINB12	Serpin B12	12907992.62	2	5.9	46.276	11.749
sp|Q86X10|RLGPB_HUMAN Ral GTPase-activating protein subunit beta OS=Homo sapiens OX=9606 GN=RALGAPB PE=1 SV=1;tr|A0A0J9YW54|A0A0J9YW54_HUMAN Ral GTPase-activating protein subunit beta (Fragment) OS=Homo sapiens OX=9606	Q86X10	RALGAPB	Ral GTPase-activating protein subunit beta	2504700.222	2	1.7	166.8	11.72

GN=RALGAPB PE=1 SV=1;tr|A2A2F0|A2A2F0								
sp|Q14525|KT33B_HUMAN Keratin, type I cuticular Ha3-II OS=Homo sapiens OX=9606 GN=KRT33B PE=1 SV=3;sp|Q15323|K1H1_HUMAN Keratin, type I cuticular Ha1 OS=Homo sapiens OX=9606 GN=KRT31 PE=1 SV=3;sp|Q14532|K1H2_HUMAN Keratin, type I cuticular Ha2 OS=Homo sapi	Q14525	KRT33 B	Keratin, type I cuticular Ha3-II	9475294.0 72	3	6.7	46.213	11.66
tr|E7EXA6|E7EXA6_HUMAN Chromosome transmission fidelity protein 18 homolog OS=Homo sapiens OX=9606 GN=CHTF18 PE=1 SV=2;sp|Q8WVB6|CTF18_HUMAN Chromosome transmission fidelity protein 18 homolog OS=Homo sapiens OX=9606 GN=CHTF18 PE=1 SV=1;tr|A0A0D9SF58|A0A0D	E7EXA6	CHTF1 8	Chromosome transmission fidelity protein 18 homolog	3975599.3 32	2	2.5	107.43	11.608
sp|Q5T5Y3|CAMP1_HUMAN Calmodulin-regulated spectrin-associated protein 1 OS=Homo sapiens OX=9606 GN=CAMSAP1 PE=1 SV=2	Q5T5Y3	CAMS AP1	Calmodulin-regulated spectrin-associated protein 1	2998500.9 78	2	2.2	177.97	11.387
sp|Q8N9T8|KRI1_HUMAN Protein KRI1 homolog OS=Homo sapiens OX=9606 GN=KRI1 PE=1 SV=3;tr|A0A494C108|A0A494C108_HUMAN Protein KRI1 homolog OS=Homo sapiens OX=9606 GN=KRI1 PE=1 SV=1	Q8N9T8	KRI1	Protein KRI1 homolog	3429802.1 1	2	3.4	82.597	11.283
sp|Q8N4C8|MINK1_HUMAN Misshapen-like kinase 1 OS=Homo sapiens OX=9606 GN=MINK1 PE=1 SV=2;tr|A0A590UJE1|A0A590UJE1_HUMAN Misshapen-like kinase 1 (Fragment) OS=Homo	Q8N4C8	MINK1	Misshapen-like kinase 1	1295399.7 79	4	3.3	149.82	6.7898

sapiens OX=9606 GN=MINK1 PE=1 SV=1;tr|I3L2I2|I3L2I2_HUMAN Misshapen-like kinase 1 (Fragment)								
sp|Q14CN4|K2C72_HUMAN Keratin, type II cytoskeletal 72 OS=Homo sapiens OX=9606 GN=KRT72 PE=1 SV=2	Q14CN4	KRT72	Keratin, type II cytoskeletal 72	13086991.74	6	8.6	55.877	6.2774
sp|Q9H2K8|TAOK3_HUMAN Serine/threonine-protein kinase TAO3 OS=Homo sapiens OX=9606 GN=TAOK3 PE=1 SV=2	Q9H2K8	TAOK3	Serine/threonine-protein kinase TAO3	2168201.027	3	3.2	105.4	6.2597
tr|E5RI98|E5RI98_HUMAN Nucleophosmin OS=Homo sapiens OX=9606 GN=NPM1 PE=1 SV=2;tr|A0A7I2V5J8|A0A7I2V5J8_HUMAN Nucleophosmin OS=Homo sapiens OX=9606 GN=NPM1 PE=1 SV=1	E5RI98	NPM1	Nucleophosmin	6302696.514	15	47.8	25.294	6.1644

Table S2. List of the seventeen proteins identified by LC-MS/MS possible survived to filtering process for further analysis validation.

Gene name	Protein name	Peptides	Sequence coverage [%]	Mol. weight [kDa]	Score
CLTC	Clathrin heavy chain 1	73	48.2	191.61	323.3
MYOF	Myoferlin	50	30.6	234.71	323.3
ATP1A1	Sodium/potassium-transporting ATPase subunit alpha-1	27	29.8	112.89	272.2
TJP2	Tight junction protein ZO-2	31	29.8	133.96	241.8
ITGA2	Integrin alpha-2	24	24.6	129.29	172.3
TJP1	Tight junction protein ZO-1	21	16	195.46	137.5
ITGB1	Integrin beta-1	18	24.3	82.23	135.4
ATP2B1	Plasma membrane calcium-transporting ATPase 1	10	10.8	134.68	133.0
ITGA3	Integrin alpha-3	13	12.7	116.61	118.1
MYO1B	Unconventional myosin-Ib	16	16.6	128.48	114.5
PPP1R12A	Protein phosphatase 1 regulatory subunit 12A	14	16.4	115.28	96.4
CC2D1A	Coiled-coil and C2 domain-containing protein 1A	14	18.9	104.06	90.7
RAPH1	Ras-associated and pleckstrin homology domains-containing protein 1	13	12	135.25	87.3
EPHA2	Ephrin type-A receptor 2	11	13.2	108.27	83.3
FMNL1	Formin-like protein 1	10	10.3	121.85	69.6
PPFIBP1	Liprin-beta-1	9	12.4	114.02	61.9
CD44	CD44 antigen	7	10.2	81.537	44.8

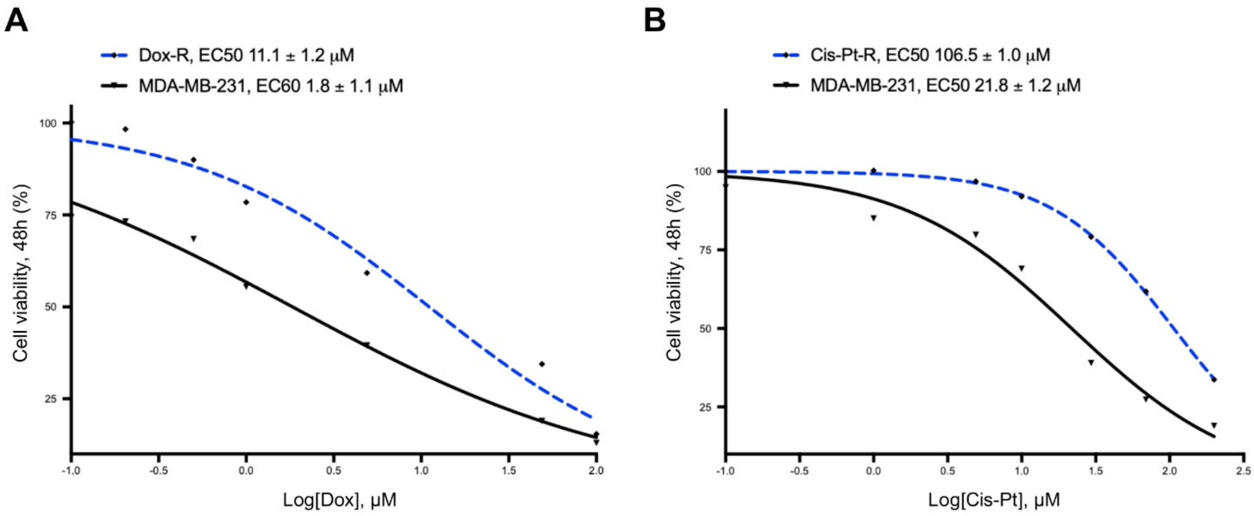

Fig. S1. Dox-R and Cis-Pt-R cells exhibit increased in EC50 concentration with respect to parental MDA-MB-231 cells. Following 48 h of Dox (A) or Cis-Pt (B) treatment, cell viability was assessed and the EC50 value for each treatment was calculated as described in Methods. Data are representative of three independent experiments.

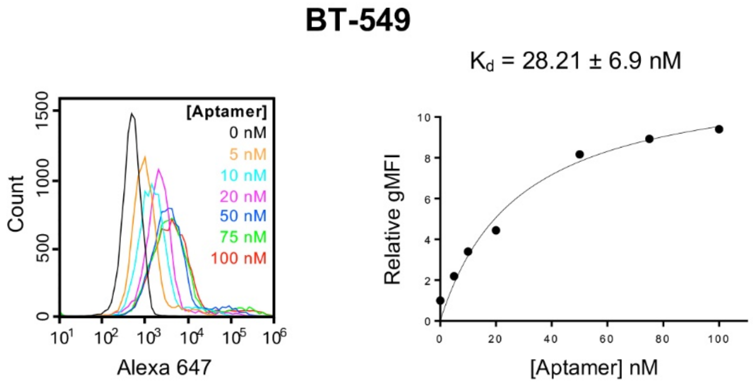

Fig. S2. Binding assay of Alexa 647-sTN58 to TNBC MES BT-549 cells using flow cytometry. *Left*, representative binding profiles of increasing concentrations of Alexa 647-sTN58 incubated with BT-549 cells. *Right*, apparent dissociation curve of aptamer-cell interaction and Kd value of sTN58 is shown.

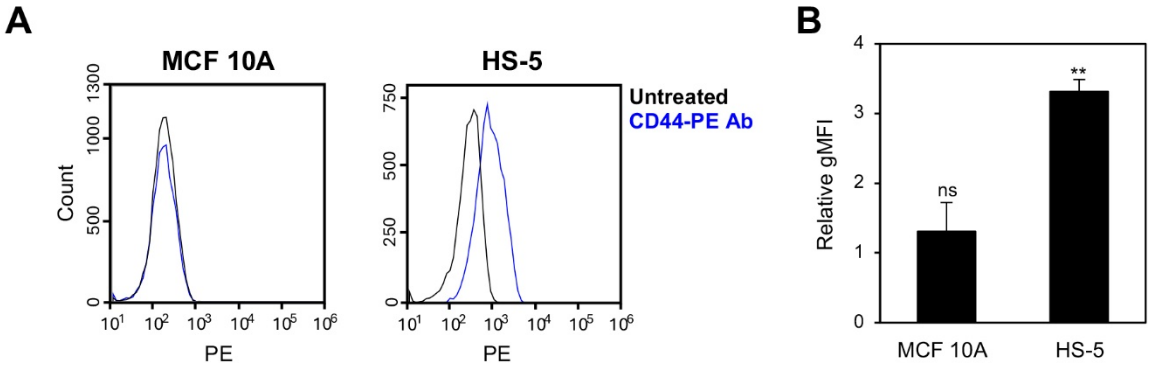

Fig. S3. CD44 expression in MCF 10A and HS-5 cells by flow cytometry. (A) Flow cytometry analyses of MCF 10A and HS-5 cells treated with CD44-PE Ab. (B) Quantification of the gMFI of CD44-PE Ab-treated cells normalized to the gMFI of the untreated cells. Bars depict mean ± SD of two independent experiments. ***P*<0.01, relative to untreated cells; ns, no significant.

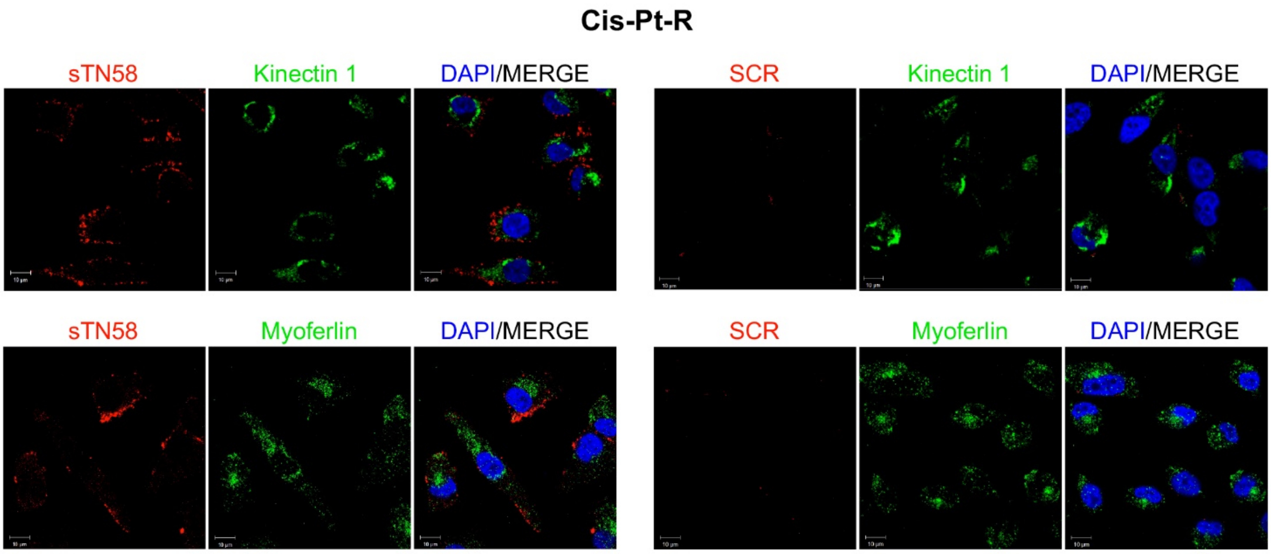

Fig. S4. sTN58 shows a different staining pattern with anti-kinectin 1 and anti-myoferlin antibodies. Confocal microscopy analysis of Cis-Pt-R cells incubated with 2 μM Alexa647-sTN58 or Alexa647-SCR, and then fixed and stained with the indicated antibodies. Aptamers, antibodies and nuclei are visualized in red, green and blue, respectively. All digital images were captured at the same setting to allow direct comparison of staining patterns. Magnification 63×, 1.0× digital zoom, scale bar = 10 μm.

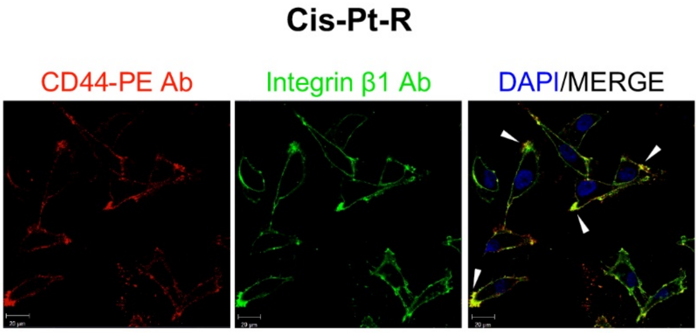

Fig. S5. CD44 and integrin β1 are associated on Cis-Pt-R cell surface. Confocal microscopy analyses of Cis-Pt-R cells stained with CD44-PE and integrin β1 antibodies. All digital images were captured at the same setting to allow direct comparison of staining patterns. Magnification 63×, 0.5× digital zoom, scale bar = 20 μm. Co-localization results appear yellow in the merged images. Arrowheads indicate some co-localization points between integrin β1 Ab and CD44-PE Ab (Overlap Coefficient, 0.72).

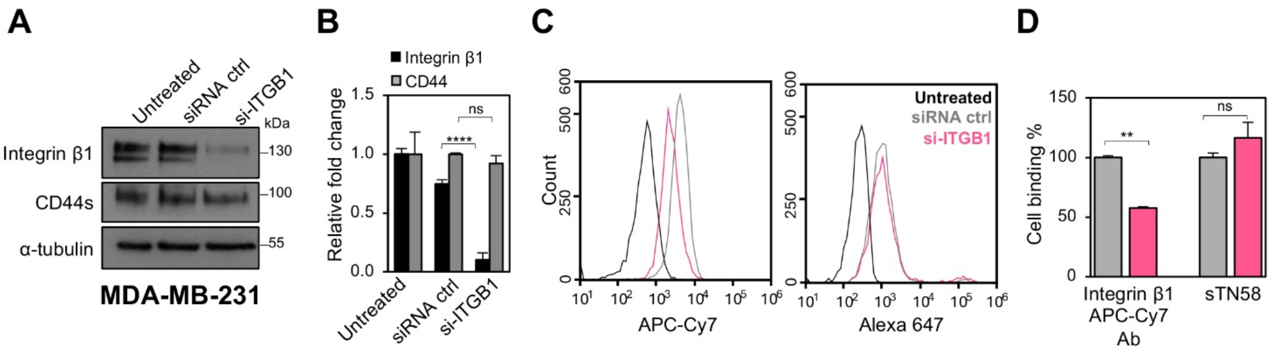

Fig. S6. Integrin β1 silencing does not affect sTN58 cell binding. (A) Cell lysates from MDA-MB-231 cells left untreated or treated for 48h with 100nM siRNA ctrl or si-ITGB1 were analyzed by immunoblotting with integrin β1 Ab, CD44 Ab and anti-α-tubulin antibody (used as a loading control). Molecular weights of protein markers are reported. (B) The histogram indicates the protein expression/α-tubulin ratio of the densitometric signals. Values are shown relative to untreated, arbitrarily set to 1. (C) Binding of integrin β1-APC-Cy7 Ab (*left*) and Alexa647-sTN58 (*right*) to MDA-MB-231 cells following 48 h transfection with siRNA ctrl (gray) and si-ITGB1 (pink). (D) The histogram shows gMFI of si-ITGB1-transfected cells treated with sTN58 aptamer or integrin β1-APC-Cy7 Ab, normalized to the gMFI of untreated cells, and expressed as percentage with respect to siRNA ctrl-transfected cells. (B, D) Bars depict mean ± SD of two independent experiments. ***P*<0.001, *****P*<0.0001; ns, no significant.

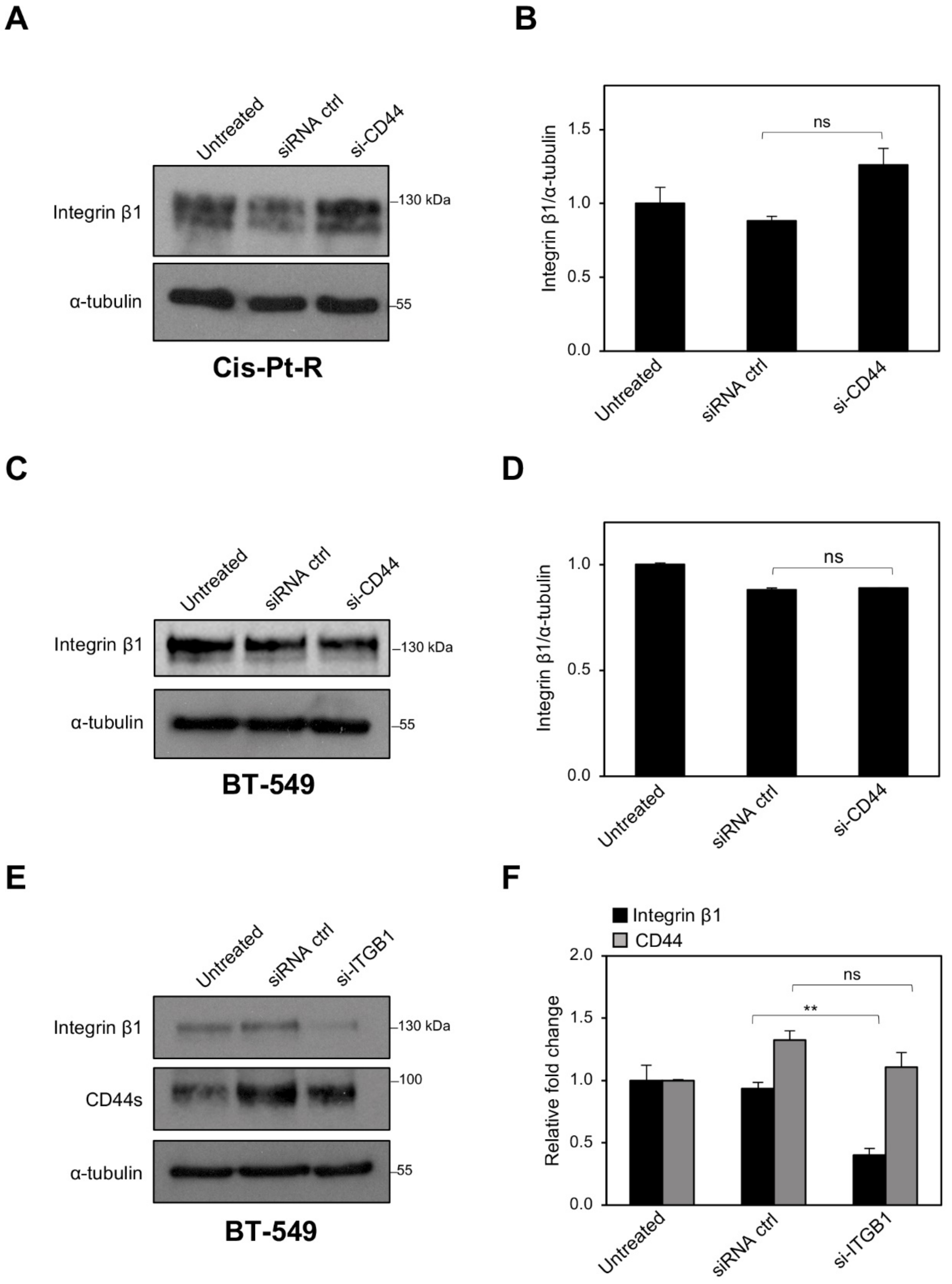

Fig. S7. Silencing of CD44 or integrin β1 does not affect the expression of integrin β1 or CD44, respectively. (A, C) Integrin β1 expression in si-CD44 transfected Cis-Pt-R (A) and BT-549 (C) cells. Filters immunoblotted with CD44 Ab, as shown in Fig. 3A (Cis-Pt-R) and E (BT-549) were stripped and immunoblotted with integrin β1 Ab. α-tubulin blots are reused since derived from the same gels shown in Fig. 3A, E. (E) Integrin β1 silencing on BT-549 cells. BT-549 cells were left untreated or

transfected with si-ITGB1 or siRNA ctrl. At 48 h post-transfection, cells were harvested, and cell lysates prepared and immunoblotted with CD44 Ab or integrin β1 Ab. Equal loading was confirmed by immunoblot with anti-α-tubulin antibody. (A, C, E) Molecular weights of protein markers are reported. (B, D, F) The histogram shows the protein expression/α-tubulin ratio of the densitometric signals. Values are shown relative to untreated, arbitrarily set to 1. ** $P<0.0$; ns, no significant.

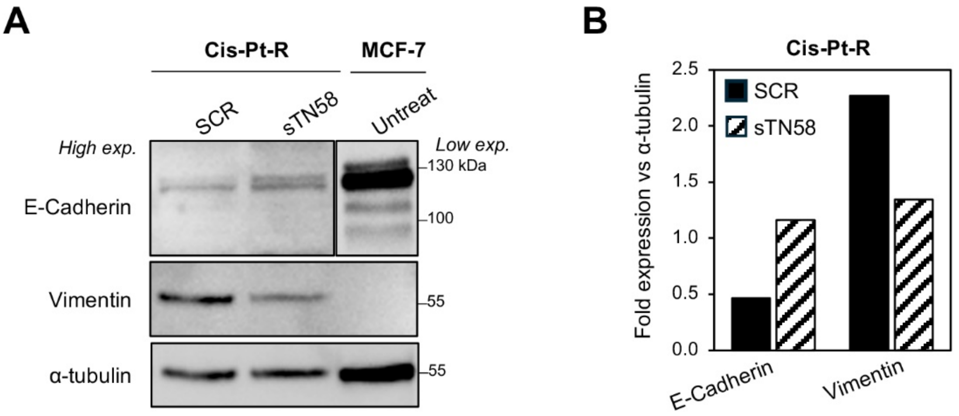

Fig. S8. Inhibition of EMT by sTN58. (A) Cis-Pt cells were grown in Matrigel for 2 days in the presence of sTN58 or SCR, as reported in the legend to Figure 5A. Cells were harvested from Matrigel and cell lysates were immunoblotted with E-Cadherin and vimentin antibodies. Molecular weights of protein markers are reported. MCF-7 cells, left untreated, were used as a control for epithelial phenotype. The black line delineates high and low exposure of the filter blotted with E-Cadherin antibody. (B) The expression of the housekeeping protein α-tubulin was used to estimate the relative fold of expression of E-Cadherin and vimentin.

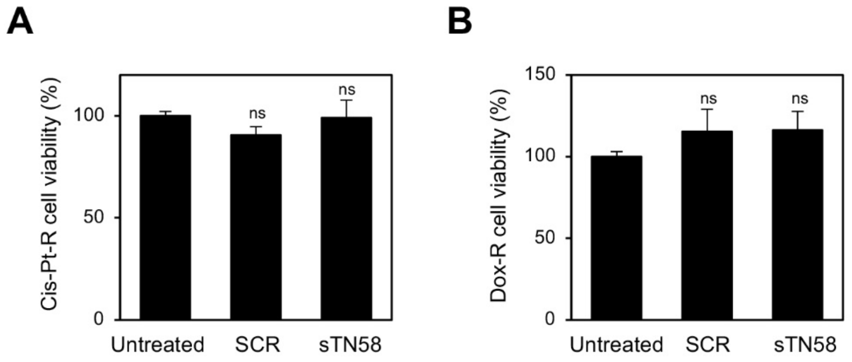

Fig. S9. sTN58 treatment does not affect cell viability. Cis-Pt-R (A) or Dox-R (B) cells were left untreated or treated for 72 h with 500 nM sTN58 or SCR. Cell viability was analyzed and expressed as percent of viable treated cells with respect to untreated cells. Each determination represents the average of two independent experiments and error bars represent SD; ns, no significant relative to untreated cells.

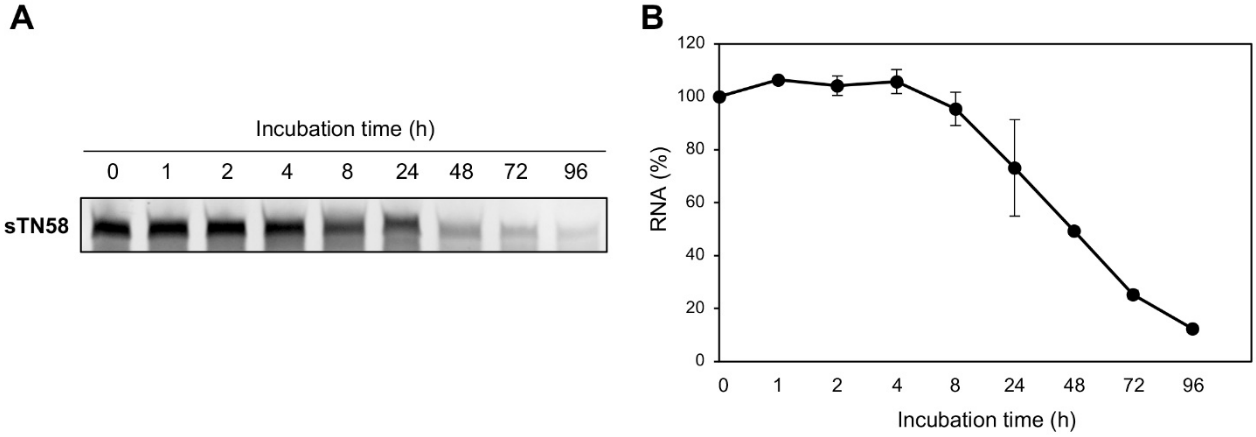

Fig. S10. In vitro serum stability analysis of sTN58 aptamer. (A) Denaturing PAGE of sTN58 following incubation with 80 % human serum at the indicated times. Depicted results represent one of two typical experiments performed. sTN58 aptamer contains 2′F-Py in the entire sequence to increase nuclease resistance. (B) Band intensity was quantified using ImageJ (v1.46r) at each time point and expressed as a percentage with respect to time 0 RNA. Note that time 0 was taken after 1 h incubation with proteinase K. Data shown are mean ± SD of two independent experiments.

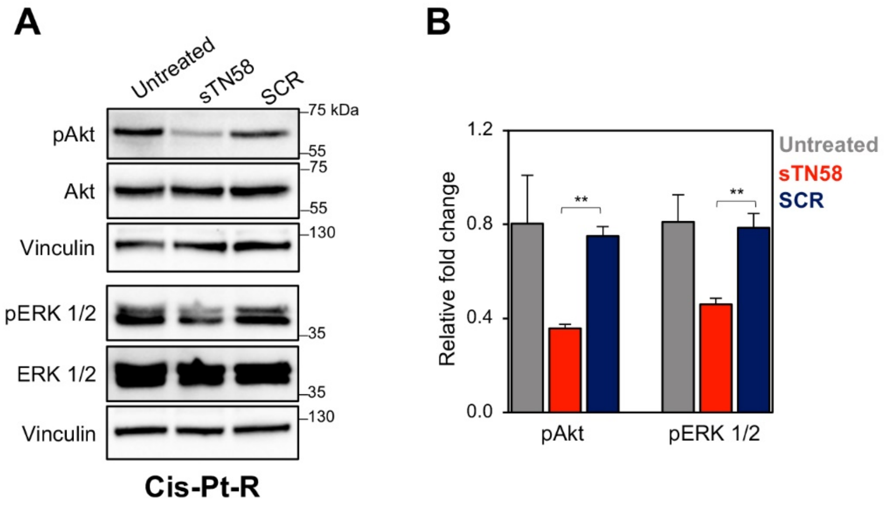

Fig. S11. sTN58 inhibits ERK 1/2 and Akt activation in Cis-Pt-R cells. (A) Cis-Pt-R cells were left untreated or treated with 500 nM sTN58 or SCR aptamers for 1 h. Cell lysates were immunoblotted with the indicated antibodies. Equal loading was confirmed by immunoblot with anti-vinculin antibody. Molecular weights of protein markers are reported. (B) The histogram shows the protein expression/vinculin ratio based on the densitometric signals. Bars depict mean ± SD of at least two independent experiments.
